# *N*-Glycome Profile of the Spike Protein S1: Systemic and Comparative Analysis from Eleven Variants of SARS-CoV-2

**DOI:** 10.3390/biom13091421

**Published:** 2023-09-20

**Authors:** Cristian D. Gutierrez Reyes, Sherifdeen Onigbinde, Akeem Sanni, Andrew I. Bennett, Peilin Jiang, Oluwatosin Daramola, Parisa Ahmadi, Mojibola Fowowe, Mojgan Atashi, Vishal Sandilya, Md Abdul Hakim, Yehia Mechref

**Affiliations:** Department of Chemistry and Biochemistry, Texas Tech University, Lubbock, TX 79409, USA; cristian.d.gutierrez-reyes@ttu.edu (C.D.G.R.); sonigbin@ttu.edu (S.O.); aksanni@ttu.edu (A.S.); andy.bennett@ttu.edu (A.I.B.); peilin.jiang@ttu.edu (P.J.); odaramol@ttu.edu (O.D.); pahmadi@ttu.edu (P.A.); mfowowe@ttu.edu (M.F.); mojgan.atashi@ttu.edu (M.A.); vishal.sandilya@ttu.edu (V.S.); md-abdul.hakim@ttu.edu (M.A.H.)

**Keywords:** SARS-CoV-2, *N*-glycans, isomers, spike protein

## Abstract

The SARS-CoV-2 virus rapidly spread worldwide, threatening public health. Since it emerged, the scientific community has been engaged in the development of effective therapeutics and vaccines. The subunit S1 in the spike protein of SARS-CoV-2 mediates the viral entry into the host and is therefore one of the major research targets. The S1 protein is extensively glycosylated, and there is compelling evidence that glycans protect the virus’ active site from the human defense system. Therefore, investigation of the S1 protein glycome alterations in the different virus variants will provide a view of the glycan evolution and its relationship with the virus pathogenesis. In this study, we explored the *N*-glycosylation expression of the S1 protein for eleven SARS-CoV-2 variants: five variants of concern (VOC), including alpha, beta, gamma, delta, and omicron, and six variants of interest (VOI), including epsilon, eta, iota, lambda, kappa, and mu. The results showed significant differences in the *N*-glycome abundance of all variants. The *N*-glycome of the VOC showed a large increase in the abundance of sialofucosylated glycans, with the greatest abundance in the omicron variant. In contrast, the results showed a large abundance of fucosylated glycans for most of the VOI. Two glycan compositions, GlcNAc_4_,Hex_5_,Fuc,NeuAc (4-5-1-1) and GlcNAc_6_,Hex_8_,Fuc,NeuAc (6-8-1-1), were the most abundant structures across all variants. We believe that our data will contribute to understanding the S1 protein’s structural differences between SARS-CoV-2 mutations.

## 1. Introduction

The COVID-19 pandemic, caused by the severe acute respiratory syndrome coronavirus 2 (SARS-CoV-2), spread rapidly across the globe as early as fall 2019 [1]. By August 2023, the World Health Organization (WHO) had recorded approximately 770 million cumulative cases of COVID-19 worldwide, and 6.96 million deaths. The large number of deadly cases is alarming, especially considering the possibility of unreported cases [2]. Since the SARS-CoV-2 virus appeared, scientists have shifted attention to combating the infection by revealing the structure of the virus, understanding its mechanisms, and developing potent vaccines and treatments [3]. Following the emergence of the wild type, the SARS-CoV-2 virus has rapidly mutated into multiple variants of concern, with each variant adopting different biological characteristics that cause them to escape the activities of the immune system [4,5,6]. The alpha, beta, gamma, delta, and omicron variants are characterized by greater infectivity, transmissibility, and immune escapism, and are thus referred to as VOC. Other variants, such as epsilon, eta, iota, lambda, kappa, and mu, also have some specific biological attributes, sharing similar mutations with the VOC, but they are not as virulent and widely spread as the VOC [7,8]. These other types of SARS-CoV-2 variants are referred to as VOI. The rushed vaccine development against the SARS-CoV-2 virus produced vaccines that have demonstrated reduced efficacy for protection against the different variants of concern, thereby complicating the fight against the pandemic [9,10,11,12].

The pathogenesis of the SARS-CoV-2 virus begins with its replication in the host’s body and the incorporation of the viral transmembrane spike (S) glycoprotein into the host’s cell through the binding of angiotensin-converting enzyme 2 receptor (ACE2) on the surface of the cell and the receptor-binding domain (RBD) on the viral spike glycoprotein [13,14,15]. This allows viral entry into the target cell. For this, the surface unit S1 region on the spike glycoprotein interacts with and binds to ACE2, while the surface unit S2 region is involved in the fusion of the viral membrane with the membrane of the host cell [15,16]. Glycans that decorate the spike protein surface are important for critical activities, including viral attachment, membrane fusion, and the replication of the virus. A large number of putative *N*- and *O*-glycosylation sites have been identified in the S protein: 22 *N*-sites and 58 *O*-sites [17,18]. The S1 glycoprotein is more densely glycosylated, carrying thirteen of the total S protein sites, but the glycan heterogeneity of both subunits is similar; these differences were clearly addressed by Watanabe et al. [19]. In the subunit S1, the sites N17, N74, N149, N165, N282, N331, N343, N616, and N657 contain a large abundance of complex glycans with high sialylation and fucosylation. Similarly, for the subunit S2, these glycan types are largely observed in the sites N1098, N1134, N1158, N1173, and N1194 [17,19]. The high mannose glycan types were common in the N61, N122, and N603 sites located in the subunit S1, and the N717, N801, and N1074 sites from the subunit S2 [17,19]. In addition, glycans play vital roles in immune recognition and response and direct the design of vaccines against SARS-CoV-2 virus mutations. Thus, the viral S protein plays a pivotal role in the pathogenesis of the virus [16,20]. Most viral fusion proteins employ a thick coating of *N*-glycans on their surface during pathogenesis and this has been reported to be similar in the case of SARS-CoV-2, although a different glycosylation pattern was reported [21,22]. At this time, the development of therapeutics for COVID-19 is the overarching goal of the scientific community, and owing to the ultimate role of the S protein in the pathogenesis of the COVID-19 virus, especially the S1 region, designing therapeutics that target its RBD domain is necessary [23]. SARS-CoV-2 has previously been reported to have an interaction with sialylated glycans on the surfaces of host cells [24,25]. Moreover, distributive glycan isomers were previously reported in a study that compared *N*-glycan isomers in MERS-CoV, SARS-CoV-1, and SARS-CoV-2 [26]. These studies indicated the glyco-connection between the S1 region of the SARS-CoV-2 S protein and the target host.

The completion of an *N*-glycan profile is challenging due to the structural heterogeneity and complexity of glycans and their isomers, including positional, linkage, and branching isomeric structures [27]. LC-MS/MS analysis has proven to be an excellent tool to achieve important glycan characterization [28,29]. Derivatization reactions such as permethylation facilitate glycan analysis [30,31,32,33], and the permethylation technique confers several advantages in glycomics studies. For example, it is possible to use reversed-phase liquid chromatography (RPLC) with the produced hydrophobic glycan ethers. Glycan permethylation also reduces fucose unit rearrangement in glycan structures [34].

In this study, we utilized an LC-MS/MS approach to investigate and profile *N*-glycosylation patterns, including a brief glycan isomeric description of the S1 region of the spike S protein, from eleven variants of SARS-CoV-2, including the alpha, beta, gamma, delta, epsilon, eta, iota, lambda, mu, and omicron variants. Although *N*-glycomics studies have been conducted on some of the VOC [35], neither complete characterization nor isomeric profiling have ever been investigated. Moreover, the *N*-glycomics studies were extended to the VOI that have been previously ignored, providing information that could offer insights into the evolution of the SARS-CoV-2 virus. A profound understanding of the *N*-glycan conformations in different variants of SARS-CoV-2 would help to elucidate the pathogenesis of the virus and improve the development of vaccine candidates that could elicit biologically relevant immune responses [36]. In the future, we believe it would be interesting to explore the specific roles that different glycan isomers play in each variant.

## 2. Materials and Methods

### 2.1. Chemicals and Reagents

Acetic acid, ammonium bicarbonate (ABC), borane–ammonia, formic acid (FA), and sodium hydroxide (NaOH) beads were acquired from Sigma Aldrich (St. Louis, MO, USA). PNGase F enzyme was purchased from New England Biolabs (Ipswich, MA, USA). HPLC-grade acetonitrile (MeCN), methanol (MeOH), and water were purchased from Fisher Sci. (Fair Lawn, NJ, USA). Micro-columns were acquired from HA (Holliston, MA, USA), and the cartridges Isolute^®^ C18 (EC) were acquired from Biotage (Charlotte, NC, USA). SARS-CoV-2 (2019-nCoV) spike S1 protein variants were obtained from Sino Biologicals US Inc., Wayne, PA, USA (see Table 1).

### 2.2. N-Glycan Preparation

As previously described, the *N*-glycans were enzymatically digested using PNGase F [37]. The samples were prepared in triplicate. Briefly, 10 μg of protein was diluted to 50 μL with 50 mM ABC buffer and subjected to denaturization at 90 °C for 15 min. Then, 1000 U of PNGase F were added to the samples and incubated at 37 °C overnight. The digested samples were dried and reconstituted in 300 μL of 5% acetic acid. Meanwhile, the SPE C18 cartridges were washed with 3 mL of MeOH and equilibrated with 3 mL of an aqueous solution of 5% acetic acid. The reconstituted samples were loaded to the cartridges and washed with 300 μL of 5% acetic acid, 3 times. The flow-through was recovered in 1.5 mL tubes and dried using a SpeedVac. The released and purified *N*-glycans were reduced using ammonium–borane followed by incubation at 60 °C for 1 h. The residual borane was removed by the addition of MeOH, and the evaporation of the methyl borate complex formed. The reduced *N*-glycans were permethylated as follows: the samples were reconstituted in 30 μL of DMSO, 1.2 μL of water, and 20 μL of iodomethane. Then, the sample solution was loaded into a micro-spin column packed with NaOH beads and incubated for 25 min in darkness at room temperature. A second incubation period of 15 min was followed by the addition of 20 μL of iodomethane. The permethylated *N*-glycans were recovered by centrifugation at 1800 rpm, dried, and reconstituted in an aqueous solution containing 20% acetonitrile and 0.1% of FA prior to LC-MS analysis.

### 2.3. N-Glycan Profiling

A previously developed in-house methodology [38] was used to complete the *N*-glycan profile of the SARS-CoV-2 spike protein S1 of the variants alpha, beta, gamma, delta, epsilon, eta, iota, kappa, lambda, mu, and omicron. Two micrograms of the permethylated *N*-glycan sample were introduced into the LC-MS system. The samples were separated on an UltiMate 3000 nanoUHPLC system (Thermo Sci., San Jose, CA, USA) using a 50 cm C18 column. For loading and online purification, an Acclaim PepMap 100 C18 trap column was used (75 mm × 2 cm, 3 mm particle size, Thermo Sci.) with a flow rate of 3 mL/min of mobile phase A (MPA) by 10 min. The composition of MPA was 98% HPLC water with 2% acetonitrile and 0.1% of FA; mobile phase B was 98% acetonitrile with 2% water and 0.1% of FA. A chromatographic gradient with a column temperature of 55 °C and flow rate of 0.2 mL/min was used. Initially, MPB was 20% over 35 min and increased to 42% in 5 min. Then, it was gradually increased to 55% in 125 min, increased to 90% in 3 min, and kept constant for 17 min. Finally, it was decreased to 20% in 5 min and we equilibrated the column for 10 min. The nanoUHPLC system was coupled to a QExactive HF (Thermo Sci., San Jose, CA, USA) and operated in positive ion mode. The spray voltage was set at 1.6 kV with a capillary temperature of 275 °C. The full scan was performed with a 120 K resolution, AGC target of 1e5, FWHM of 30 s, Max IT of 100 ms, and a scan range of 600 to 1800 *m*/*z*. The MS/MS spectra were acquired in data-dependent mode for the top 20 most intense ions with a 15 K resolution. Stepped high-energy collision dissociation (sHCD) was used as a fragmentation technique with N(CE) values of 15, 25, and 35. The AGC target was set at 1e5 with a Max IT of 50 ms, isolation window of 4 *m*/*z*, and a dynamic exclusion of 20 s.

### 2.4. Protein Verification of the SARS-CoV-2 S1 Variants

Proteomics samples were prepared in triplicate. The equivalent of 10 μg of protein was denatured at 90 °C for 15 min and alkylated using iodoacetamide. Briefly, the denatured protein sample was reduced using 1.25 μL of 200 mM DTT and incubated in a water bath for 45 min at 60 °C. Then, the reduced protein was alkylated using 5.0 μL of 200 mM IAA and incubated in a water bath for 45 min at 37 °C. The excess of IAA was quenched with a second addition of 1.25 μL of 200 mM DTT and further incubated for 30 min at 37 °C. The alkylated protein was digested using trypsin at a concentration ratio of enzyme to protein of 1:25 and incubated overnight at 37 °C. After incubation, the digestion was stopped at 90 °C for 15 min, and the samples were dried in a SpeedVac.

The dried tryptic digests were reconstituted in an aqueous solution of 2% acetonitrile with 0.1% FA. The volume of sample equivalent to 1 μg was injected onto a C18 trap column (75 μm × 2 cm, 2 μm, 100 Å; Thermo Sci., Pittsburgh, PA, USA) for loading and online purification lasting 10 min. Thereafter, the samples were directed to an Aclaim PepMap C18 capillary column (75 μm × 15 cm, 2 μm, 100 Å; Thermo Scientific, Pittsburgh, PA, USA) using an UltiMate 3000 nanoUHPLC system (Dionex, Sunnyvale, CA, USA). The flow rate was set to 0.3 μL/min with a temperature of 30 °C. MPA was an aqueous mixture with 2% of acetonitrile and 0.1% of FA, while MPB was a mixture of acetonitrile with 2% of water and 0.1% of FA. The analytical gradient was as follows: 2% MPB was maintained for the first 10 min; then, the percentage of MPB was increased to 30% from 11 to 95 min, and increased to 50% at 110 min. MPB was then increased from 50% to 90% from 110 to 113 min. The gradient was kept constant at 90% MPB for the next 5 min, and then dropped to 2% MPB from 118 to 119 min. Finally, 2% MPB was maintained from 119 to 130 min. The nanoUHPLC system was interfaced to an Orbitrap Fusion Lumos Tribrid mass spectrometer (Thermo Sci., San Jose, CA, USA) and operated in positive ion mode. The spray voltage was set at 1.6 kV with a capillary temperature of 275 °C. The full scan was isolated in the quadrupole and detected in the Orbitrap^®^ mass analyzer with a resolution of 120 K, standard AGC, an LC peak width of 35 s, and a scan range of 400 to 1800 *m*/*z*. The MS/MS spectra were acquired with a duty cycle of 3 s and a resolution of 60 K. High-energy collision dissociation (HCD) was used as a peptide dissociation technique with an NCE value of 25. The AGC target was set as standard, and the injection time mode was set as auto with one microscan.

### 2.5. Data Analysis

For the *N*-glycomics analysis, the raw data were revised using the Xcalibur 4.2 (Thermo Scientific) software. The retention time, MS, and MS/MS spectra were confirmed with a mass tolerance of 5 ppm. The peak areas were computed manually and used to normalize the abundance of the identified *N*-glycans (Supplementary Tables S1 and S2, respectively). Supplementary Table S3 shows the peak areas for the isomeric *N*-glycans. Descriptive statistics were used to quantitatively describe the *N*-glycosylation differences across the investigated SARS-CoV-2 protein S1 variants. For the proteomics analysis, the specific FASTA files were adjusted for each SARS-CoV-2 S1 protein variant in accordance with the mutations reported by the vendor (Table 1). Then, the Proteome Discoverer 2.5 software (Thermo Sci.) was used to calculate the percent protein coverage, as shown in Supplementary Figure S1.

## 3. Results

### 3.1. Analytical Workflow

In this work, the *N*-glycan profiles derived from the S1 proteins of eleven different variants of SARS-CoV-2 were investigated. We analyzed the VOC alpha (H12), beta (H23), gamma (H15), delta (H17), and omicron (H41), as well as the VOI epsilon (H14), eta (H29), iota (H28), lambda (H32), kappa (H1-B), and mu (H38). According to the WHO, the variants originated worldwide: Africa (gamma, eta, and omicron); Asia (beta and kappa); Europe (alpha and eta); North America (delta and iota); and South America (epsilon, lambda, and mu). Additionally, proteomics experiments were completed on the eleven SARS-CoV-2 S1 protein variants. The results are described in Supplementary Figure S1. For the proteomics analysis, the recombinant glycoproteins were tryptic-digested prior to LC-MS analysis. The average protein coverage of the variants was 87% +/− 6% (Supplementary Figure S1). Full characterization of the SARS-CoV-2 S1 protein can be achieved by adding a subsequent digestion with the enzyme Glu-C or chymotrypsin [39]. The protein coverage was analyzed utilizing the Proteome Discoverer 2.5 (Thermo Sci.) software with a 95% confidence level.

**Figure 1:**
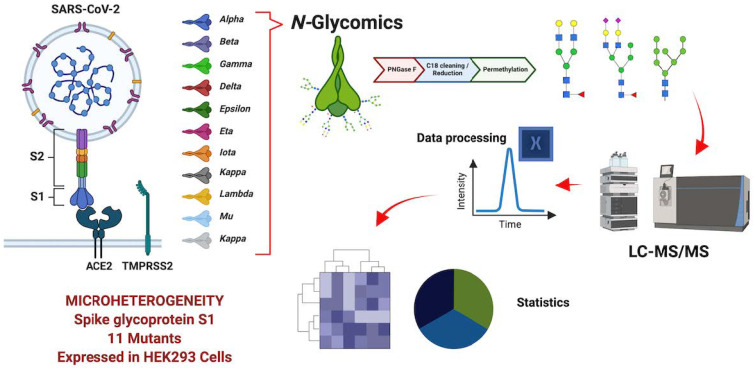
Workflow for the LC-MS/MS analysis. A four-digit *N*-glycan nomenclature was used in the following order: *N*-acetylglucosamine, hexose, fucose, *N*-acetylneuraminic acid (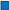 HexNAc, 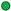 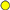 Hex, 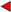 Fuc, 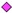 Neu5Ac). The S1 protein derived from eleven SARS-CoV-2 variants was subjected to *N*-glycomics analysis. The PNGase F released glycans were reduced and permethylated prior to LC-MS/MS analysis. Then, the data were normalized based on the total abundance of the expression of the *N*-glycans across the variants.

The experimental workflow of this approach is shown in Figure 1. A permethylation reaction was completed to enhance the ionization efficiency and structural stability of the *N*-glycans during the LC-MS analysis [37]. For the *N*-glycan identification, the theoretical *m*/*z* values of the most abundant ionic molecular species were selected with a mass tolerance of 5 ppm. The identified peaks were manually integrated and the peak areas computed; then, the peak signals were normalized using the total glycan abundance. Thereafter, the *N*-glycan profiles of the eleven S1 protein variants were compared. A four-digit nomenclature was used to simplify the annotation of the *N*-glycan structures, “1-1-1-1”, where the monosaccharides associated are represented for each numeric digit following the order *N*-acetylglucosamine, hexose, fucose, *N*-acetylneuraminic acid (HexNAc, Hex, Fuc, NeuAc).

### 3.2. N-Glycan Profile of SARS-CoV-2 Spike S1 Protein Variants

The S1 subunit has thirteen putative *N*-glycosylation sites, specifically in the amino acid positions N17, N61, N74, N122, N149, N165, N234, N282, N331, N343, N603, N616, and N657 [17]. The *N*-site modifications that could affect the *N*-glycosylation of the variants, due to its mutation in the amino acid asparagine “N,” are alpha (N501Y); epsilon (T20N, N581Y); gamma (K417N); iota (S477N); lambda (D523N); mu (Y145N, N501Y); and omicron (K417N, S477N, N440K, N501Y, N679K), as shown in Table 1. The putative *N*-site modifications of the mutants are described in Table 2; due to amino acid substitutions, deletions, or additions, the glycosylation positions changed with respect to the native S1 protein [17].

A total of 108 unique *N*-glycan compositions were identified across the analyzed SARS-CoV-2 S1 protein variants (Supplementary Tables S1 and S2). Table 2 compares the total number of *N*-glycans identified for the eleven variants. Table 2 describes the number of *N*-glycan compositions and the number of isoforms observed in the SARS-CoV-2 variants. The distribution of fucose, sialylated, sialofucosylated, *N*-acetylglucosamine, and mannose *N*-glycan types and the expected *N*-glycosylation sites are also described in Table 2. Supplementary Figure S2 shows bar plots of the relative abundance of all the *N*-glycans observed across the variants. Supplementary Figure S3 shows the bar plots of the total *N*-glycan abundance (peak area).

### 3.3. N-Glycosylation Differences between SARS-CoV-2 Spike S1 Protein Variants

**Figure 2:**
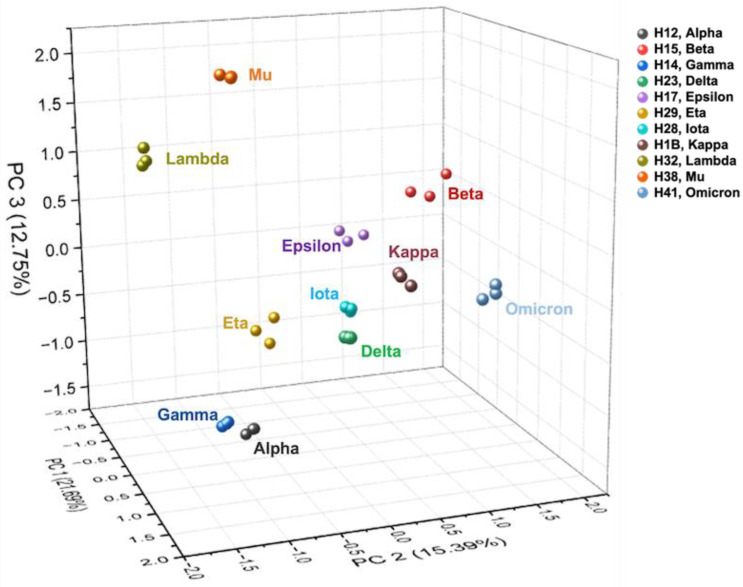
Three-dimensional principal component analysis (PCA), *N*-glycans of SARS-CoV-2 S1 protein variants: H12 alpha, H15 beta, H14 gamma, H17 delta, H14 epsilon, H29 eta, H28 iota, H1B kappa, H32 lambda, H38 mu, and H41 omicron. The VOC are all located in the lower section of the plot, which shows their glycosylation similarities.

**Figure 3:**
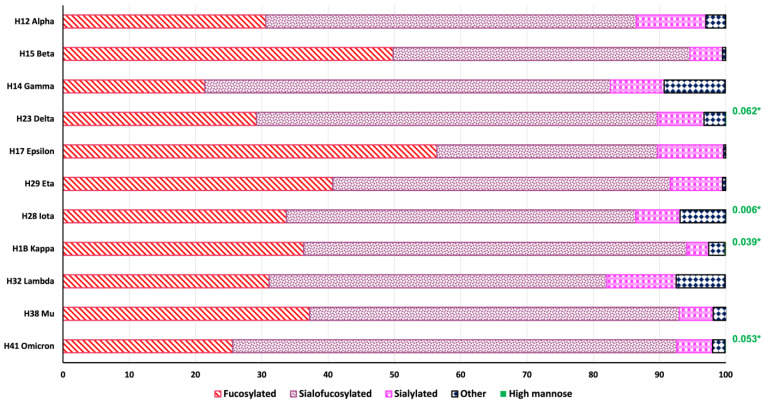
Bar plots of the relative abundance by *N*-glycan type. SARS-CoV-2 S1 protein variants: H12 alpha, H15 beta, H14 gamma, H23 delta, H17 epsilon, H29 eta, H28 iota, H1B kappa, H32 lambda, H38 mu, and H41 omicron. We observe that the primary contributor to the total protein glycosylation comes from the fucosylated and sialo-fucosylated *N*-glycan types. * High mannose abundances.

Principal component analysis (PCA) was used to visualize the data differences between the investigated variants. Figure 2 shows the PCA plot derived from the *N*-glycan quantitative data (Supplementary Table S1). Supplementary Figure S4 shows the PCA plot derived from the isomeric *N*-glycan quantification data in Supplementary Table S3. The *N*-glycosylation differences were assessed by *N*-glycan groups: sialylated, sialofucosylated, fucosylated, high mannose, and other types (galactosylated and *N*-acetylglucosamine). Figure 3 shows the combined distribution of the *N*-glycan types for each variant. Supplementary Figure S5 shows the relative abundance of the defined *N*-glycan types for each of the S1 protein variants. To better visualize the changes, a pattern color code was assigned to each variant. Additionally, the changes in *N*-glycan types were investigated in sub-groups using bar graphs. Supplementary Figure S6 shows the glycome modifications of the mono-, di-, tri-, and tetra-sialylated *N*-glycans between the studied SARS-CoV-2 S1 protein variants.

**Figure 4:**
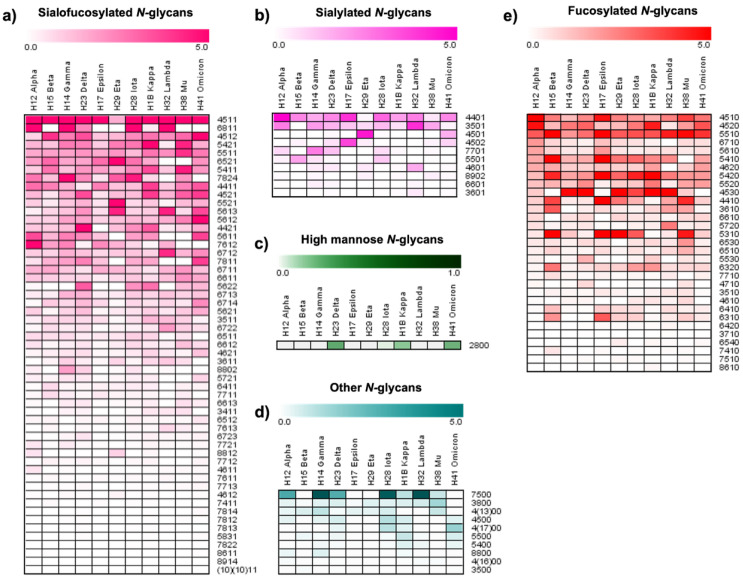
Heat maps of the relative abundance by type of *N*-glycans between the SARS-CoV-2 S1 protein variants: H12, H14, H15, H17, H23, H28, H29, H32, H38, H41, and H1B. (**a**) Fucosylated, (**b**) sialylated, (**c**) sialofucosylated, (**d**) high mannose, and (**e**) other *N*-glycan types. The glycan nomenclature is as described in Figure 1.

Heat maps were used to visualize the single *N*-glycan modifications across the analyzed variants, shown in Figure 4. The heat map of the sialofucosylated *N*-glycan type (Figure 4a) depicts a large abundance of the structures 4-5-1-1, 4-5-1-2, 4-4-1-1, 4-5-2-1, 5-6-1-3, 5-6-1-2, and 5-6-1-1, where most of them are present in the omicron variant. For the sialylated *N*-glycan types, the largest relative abundances were observed for the lambda, alpha, and epsilon variants, with 10.5, 10.5, and 10.0%, respectively. The variant with the lowest relative abundance of this glycan type was kappa with 3.3% (Supplementary Figure S5b). The sialylated N-glycan 4-4-0-1 was the most abundant structure across the variants, as shown in Figure 4b. The high mannose and the other *N*-glycan types were found to have significantly low relative abundance (Supplementary Figure S5d,e), where the glycans 2-8-0-0 and 7-5-0-0 were the most abundant structures. Additionally, the single changes in abundance of the mono-sialylated, di-sialylated, tri-sialylated, and tetra-sialylated *N*-glycan types can be observed in the heat maps described in Supplementary Figure S7.

### 3.4. Heterogeneity of the SARS-CoV-2 Spike S1 Protein Variants

**Figure 5:**
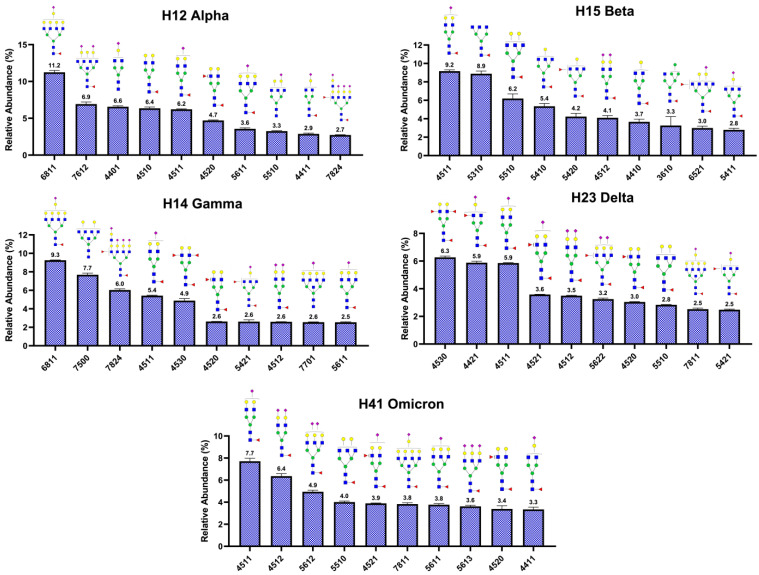
Top ten *N*-glycans, relative abundance. SARS-CoV-2 S1 protein variants of concern: H12 alpha, H14 gamma, H15 beta, H23 delta, and H41 omicron. Notice that the most abundant types of glycans observed in the S1 protein derived from the VOC are mono-fucosylated and mono-sialylated. The glycan nomenclature is described in Figure 1.

The top ten *N*-glycans were used to describe the glycome heterogeneity of the S1 protein variants. Figure 5 shows the ten most abundant *N*-glycans with their relative abundance for the S1 proteins derived from the VOC: H12 alpha, H15 beta, H14 gamma, H23 delta, and H41 omicron. Supplementary Figure S8 shows the ten most abundant *N*-glycans with their relative abundance for the S1 proteins derived from the VOI: H17 epsilon, H29 eta, H28 iota, H1B kappa, H32 lambda, and H38 mu. Interestingly, the structures represented approximately 50% of the total *N*-glycan abundance in all the investigated variants. Figure 5 shows the top ten structures for the variants of concern, where 64% of the structures were sialofucosylated, 24% fucosylated, and 12% sialylated. Among the sialylated and sialofucosylated, the mono-sialylated glycans were the most abundant glycan conformations. Supplementary Figure S8 shows the top ten structures of the variants of interest, where 40% of the structures were sialofucosylated, 40% fucosylated, and 20% sialylated.

**Figure 6:**
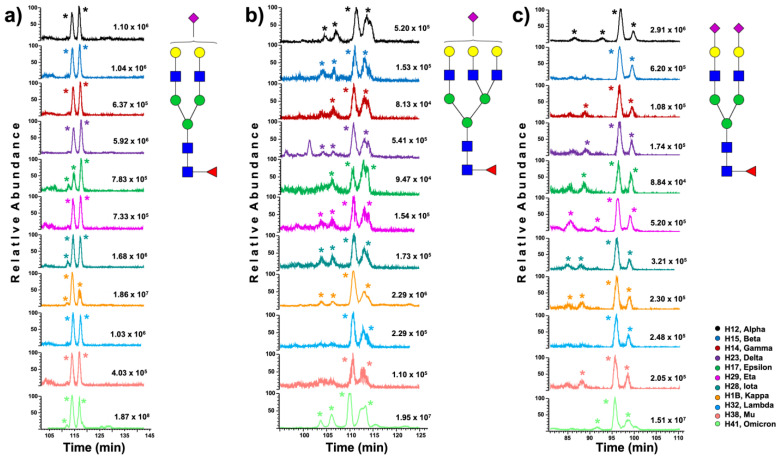
Extracted ion chromatograms (EICs) showing the isomeric expression of the *N*-glycans across SARS-CoV-2 S1 protein variants: (**a**) GlcNAc_4_, Hex_5_, Fuc, NeuAc; (**b**) GlcNAc_5_, Hex_6_, Fuc, NeuAc; and (**c**) GlcNAc_4_, Hex_5_, Fuc, NeuAc_2_. The stars (*) show the identified isoforms and glycan nomenclature as in Figure 1.

Understanding *N*-glycan diversity can be further complicated when microheterogeneity is considered, specifically for the variety of possible isoforms that can be observed in the glycan structures. Table 2 describes the total number of isoforms identified for each variant: 289 (H12), 231 (H15), 239 (H14), 299 (H23), 229 (H17), 143 (H29), 288 (H28), 306 (H1B), 147 (H32), 136 (H38), and 337 (H41). PCA was used to visualize the variant differences, shown in Supplementary Figure S4. Figure 6 shows the extracted ion chromatogram (EIC) differences in expression of three isomeric *N*-glycans across the studied S1 protein variants: GlcNAc_4_, Hex_4_, Fuc, NeuAc; GlcNAc_3_, Hex_6_, Fuc; and GlcNAc_7_, Hex_7_, Fuc_2_, NeuAc. Similar EICs for the isomeric *N*-glycans can be observed in Supplementary Figure S9: GlcNAc_4_, Hex_5_, Fuc, NeuAc; GlcNAc_5_, Hex_6_, Fuc, NeuAc; and GlcNAc_4_, Hex_5_, Fuc, NeuAc_2_.

## 4. Discussion

The SARS-CoV-2 S1 protein subunit is responsible for host cell receptor binding; it contains the receptor-binding domain (RBD) and the receptor-binding motif (RBM) that mediates the contact with the angiotensin-covering enzyme 2 (ACE2) receptor [17,23,40]. In this approach, the *N*-glycan profiles derived from the S1 proteins of eleven different variants of SARS-CoV-2 were identified, quantified, and compared. We investigated the VOC, variants that have been demonstrated to be more contagious, causing more severe disease, or to have less susceptibility to vaccines and diagnostic tests. We also investigated the VOI, variants that have been shown to possess markers that are predicted to affect transmission, or to be responsible for an increased proportion of cases. To avoid bias in the glycosylation expression, all the recombinant S1 proteins acquired were expressed in the same host, the cell line HEK293. It has been reported that the glycosylation expression of viral proteins changes significantly when different cell systems are used to produce the virus glycoprotein [41]. Wang et al. [42] found large glycosylation differences in the SARS-CoV-2 S1 protein when the protein was expressed in HEK293 and baculovirus insect cells. They reported glycosylation mainly composed for complex glycan types in the S1 protein derived from HEK293 cells. Conversely, the S1 protein derived from baculovirus insect cells had a large abundance of mannose glycan types across the protein glycosylation sites. A similar comparison was carried out by Huang et al. [43] using human lung epithelial cells BEAS-2B and the HEK293T cells to produce the SARS-CoV-2 S protein. The results showed a total abundance of 61% of complex glycans and 23% of hybrid glycans when the S protein was derived from HEK293T cells. In comparison, the total abundance of complex glycans was 78% and 1% for the hybrid glycans when the BEAS-2B cells were used to recombinantly produce the SARS-CoV-2 S protein. Recombinant viral proteins are used as a base to generate non-mRNA vaccines [44,45]; therefore, it is crucial to know the glycosylation profiles of the recombinant viral proteins produced in different cell systems.

To validate the authenticity of the recombinant glycoproteins used in our experiments, bottom-up proteomics experiments were completed on the eleven SARS-CoV-2 protein S1 variants. The glycomics experiment was performed with sequential PNGase F digestion, C18 SPE cleaning, glycan reduction–permethylation, and LC-MS/MS analysis. This protocol allowed us to have a reproducible and sensitive method [37]. Figure 1 describes the general workflow, and Table 2 describes the number of *N*-glycans identified in each variant. The largest number of *N*-glycans was observed for the omicron variant, with 91 compositions and 337 isomeric structures. The lowest number of *N*-glycans was observed for the lambda variant, with 73 compositions and 147 isomeric structures. It is worth noting that the absolute abundance (peak area) of the *N*-glycan signals observed from the S1 protein derived from the omicron variant was significantly larger than the absolute abundances observed for the rest of the variants (Supplementary Figure S3). The PCA plot (Figure 2) also differentiated the *N*-glycome of the S1 protein of the omicron variant from the other studied S1 proteins. The omicron signals can be observed in the positive PC2 quadrant of the 3D plot. Additionally, the PCA plot showed different similitudes between the *N*-glycomes derived from the S1 proteins and the other studied variants. The signals of the mu and lambda variants were observed in the positive PC3 quadrant. The signals of the gamma and alpha variants were observed in the negative PC1 quadrant. The signals for the epsilon, eta, iota, beta, kappa, and delta variants were clustered together in the center of the 3D plot.

The changes in the relative abundance of different *N*-glycan types derived from the S1 proteins were revised. Fucosylated and sialofucosylated *N*-glycans were the most abundant types of glycosylation observed in the S1 protein variants (Supplementary Figure S5a,c, respectively). The top three variants with a high abundance of fucosylated *N*-glycans were epsilon, beta, and eta, with 56.4, 49.8, and 40.7% respectively. Conversely, gamma was the variant with the lowest abundance of this *N*-glycan type, at 21.4% (Supplementary Figure S5a). In our previous glycomics studies of the native S1 protein, we reported 86% relative abundance of fucosylation, and 49% of sialylation [26]. In comparison, the results obtained from the S1 protein variants in this study showed significant changes in their *N*-glycosylation. These results indicate significant increases in fucosylation for the omicron, beta, mu, and kappa variants, with 92.6, 94.6, 92.9, and 94.1%, respectively. On the other hand, the abundance of fucosylated glycans in variants lambda and gamma decreased, with 82.0 and 82.6%, respectively. Regarding sialylated glycans, the epsilon and beta variants were observed to decrease in abundance, with 43.3 and 49.7%, respectively. In the remainder of the variants, the abundance of the sialylated glycans significantly increased up to 60% in comparison with our previous findings [26]. The most common fucosylated glycans across the native and the variants were 4-5-1-0, 4-5-2-0, and 5-5-1-0, as can be observed in the heat map in Figure 4e.

The omicron variant showed the largest relative abundance of sialofucosylated *N*-glycans with 67.0%, followed by the variants gamma, delta, and kappa with 61.2, 60.5, and 57.8%, respectively. The epsilon variant showed the lowest relative abundance of this glycan type with 33.3%, contrary to the large abundance observed for the fucosylated glycans in these variants (Supplementary Figure S5c). The *N*-glycans 4-5-1-1 and 5-4-2-1 were the most abundant structures in all the variants, as well as in the native S1 protein previously investigated [26]. The high mannose *N*-glycans were found to have significantly low relative abundance (Supplementary Figure S5d,e), where the glycans 2-8-0-0 and 7-5-0-0 were the most common structures. These results indicate a significant decrease in the high mannose glycosylation of the S1 protein variants with respect to the native S1 protein [26]. Other S1 protein glycomes derived from different coronaviruses have also shown large abundance of high mannose *N*-glycans [21]. This information suggests that the virality of the native virus may increase with the reduction of the initially large levels of high mannose glycans observed in the initial coronaviruses [46].

According to our results, the variants of concern (omicron, alpha, beta, gamma, and delta) show a large abundance of sialofucosylated *N*-glycans in the S1 protein glycome, shown in Supplementary Figure S5c. However, the fucosylated *N*-glycans are significantly low in the S1 protein glycomes of the mentioned variants (Supplementary Figure S5a). Furthermore, we studied the *N*-glycan differences in sialylation and branching among the sialylated and sialofucosylated *N*-glycans. Supplementary Figure S6 shows bar plots of the mono-, di-, tri-, and tetra-sialylated *N*-glycans. The mono-sialofucosylated *N*-glycans showed the largest number of glycan structures, and this was the most abundant type of sialylation across the S1 protein variants, with an average of 37% of the total glycosylation (Supplementary Figure S6a). There was no single mono-sialylated glycan with common expression across the variants. For example, according to the heat maps in Supplementary Figure S7a and Figure 5, the most abundant mono-sialylated glycan structures by variant were as follows: alpha (6-8-1-1); beta (4-4-1-1); gamma, delta, and iota (4-4-2-1); epsilon (4-5-1-1); eta (5-5-2-1); kappa and mu (3-4-1-1); lambda (6-4-1-1); and omicron (6-5-2-1). As for the di-sialofucosylated *N*-glycans, the omicron variant showed a relative abundance of 17.2%, significantly larger than the results observed for the other variants (Supplementary Figure S7c). The most common and abundant di-sialofucosylated glycans were 6-7-1-2 and 6-7-2-2, which showed particularly high abundance in the lambda variant (Supplementary Figure S7b). In comparison with the mono- and di-, the tri- and tetra-sialofucosylated glycans showed lower abundances (Supplementary Figure S7b–d).

Glycans are highly heterogeneous in nature; their structures vary in the composition of individual monosaccharide building blocks, their linkage, and stereochemistry [47]. The top ten *N*-glycans were used to describe the glycome heterogeneity of the S1 protein variants. In comparison, the S1 proteins derived from the SARS-CoV-2 variants of concern (Figure 5) show a significantly large number of sialofucosylated *N*-glycan compositions. The S1 proteins derived from the variants of interest (Supplementary Figure S8), however, show a significantly large number of fucosylated *N*-glycan conformations. Similar results are observed and discussed in Figure 3. Zheng et al. [48] described the functions of some glycan epitopes based on ELISA analyses. In their results, the omicron variant decreased in binding affinity to the ACE2 when the fucose moieties of the glycans were removed from the S1 protein. Although our results correlated with Zheng et al.’s findings, validating the importance of the fucosylated glycans in the binding affinity of the omicron variant with the cell ACE2, the generated data suggest that the fucose unit may be part of a sialyl Lewis X epitope due to the large abundance of sialofucosylated *N*-glycans in comparison with the less abundant fucosylated *N*-glycans. This hypothesis also correlates with previous reports indicating that sialyl Lewis X glycan receptors facilitate viral infection [49,50,51,52].

*N*-Glycan microheterogeneity is another factor that adds complexity to the study of *N*-glycosylation. The *N*-glycans can present isomeric modifications in different structural moieties, such as the sialic acid, mannose, or fucose units [28,53]. Thus, as a glycan increases in size, its structural complexity also increases. In addition to the *N*-glycan profiles of the S1 protein variants, we briefly investigated the differences in the isomeric expression across variants. The *N*-glycan isomeric separation was achieved using our previously developed methodology [38]. Supplementary Table S3 shows the relative abundance of the identified isomeric *N*-glycans. The PCA in Supplementary Figure S4 depicts clear differences in the isomeric *N*-glycan profiling. Furthermore, to describe the differences in expression of the isomeric *N*-glycans across variants, we extracted the EICs of the most representative glycoforms. Figure 6a shows the EICs of the sialofucosylated *N*-glycan HexNac_4_,Hex_4_,Fuc,NeuAc. The omicron and beta variants showed no isomeric peaks, contrary to the four isomers observed in the alpha variant. Similarly, Figure 6b shows the EICs of the hybrid glycan HexNAc_3_,Hex_6_,Fuc, where the omicron variant showed the largest number of isomeric peaks. The sialofucosylated *N*-glycan HexNAc_7_,Hex_6_,Fuc_2_,NeuAc depicted in Figure 6c presented five isomeric peaks for almost all the variants. The EICs for *N*-glycan structures HexNAc_4_,Hex_5_,Fuc,NeuAc, HexNAc_5_,Hex_6_,Fuc,NeuAc, and HexNAc_4_,Hex_5_,Fuc,NeuAc_2_ are described in Supplementary Figure S9.

The S1 protein in the spike protein from the SARS-CoV-2 virus recognizes and binds the ACE2 receptor as the primary host cell infection route. The S1 protein is highly glycosylated; accordingly, several researchers have investigated the role of the protein glycosylation and its effect on virus infectivity and transmissibility [13,15,17,19,22,23,39]. In other viruses, researchers have found that glycans are a viral strategy to escape the host immune response, where the *N*-glycans mask the virus binding domains [54,55]. In a similar way, the S1 protein of the SARS-CoV-2 virus limits antibody access to protein neutralizing epitopes [54,56]. Therefore, a detailed glycome of the S1 protein derived from the virus’s mutations can provide insights into the pathobiology of the virus as well as vaccine development [57]. 

## Figures and Tables

**Table 1:** SARS-CoV-2 S1 protein mutations, generic names, and the countries where they were first detected.

Vendor ID *	Variant Name	Lineage	Country	Mutations/Deletions/Insertions
40591-V08H12	Alpha	B.1.1.7	England	HV69-70 deletion, Y144 deletion, N501Y, A570D, D614G, P681H
40591-V08H15	Beta	B.1.351	South Africa	L18F, D80A, D215G, LAL242-244 deletion, R246I, K417N, E484K, N501Y, D614G
40591-V08H14	Gamma	P.1	Brazil	L18F, T20N, P26S, D138Y, R190S, K417T, E484K, N501Y, D614G, H655Y
40591-V08H23	Delta	B.1.617.2	India	T19R, G142D, E156G, 157-158 deletion, L452R, T478K, D614G, P681R
40591-V08H17	Epsilon	B.1.427	USA	W152C, L452R, D614G
40591-V08H29	Eta	B.1.525	UK, Nigeria	Q52R, A67V, 69-70 deletion, 144 deletion, E484K, D614G, Q677H
40591-V08H28	Iota	B.1.526	USA	L5F, T95I, D253G, S477N, E484K, D614G
40591-V08H1-B	Kappa	B.1.617.1	India	T95I, G142D, E154K, L452R, E484Q, D614G, P681R
40591-V08H32	Lambda	C.37	Peru	G75V, T76I, RSYLTPG256-252 deletion, D523N, L452Q, F490S, D614G
40591-V08H38	Mu	B.1.621	Colombia	T95I, Y144S, Y145N, R346K, E484K, N501Y, D614G, P681H
40591-V08H41	Omicron	B.1.1.529	Botswana	A67V, Δ69-70, T95I, G142D/Δ143-145, Δ211/L212I, ins214EPE, G339D, S371L, S373P, S375F, K417N, N440K, G446S, S477N, T478K, E484A, Q493R, G496S, Q498R, N501Y, Y505H, T547K, D614G, H655Y, N679K, P681H

* Vendor part numbers (Sino Biologicals Inc.).

**Table 2:** Identified *N*-glycans across variants.

Variant Name	*N*-Glycan Compositions	*N*-Glycan Types F ^1^/S ^2^/SF ^3^/N ^4^/M ^5^	Isoforms	*N*-Glycosylation Sites ^6^
Alpha	88	27/7/46/8/0	289	N17, N61, N72, N120, N146, N162, N231, N279, N328, N340, N600, N613, N654
Beta	80	25/5/46/4/0	231	N17, N61, N74, N122, N149, N165, N234, N279, N328, N340, N60, N613, N654
Gamma	85	26/8/43/8/0	239	N17, N61, N74, N122, N149, N165, N234, N282, N331, N343, N603, N616, N657
Delta	102	29/10/53/9/1	299	N17, N61, N74, N122, N149, N163, N232, N280, N329, N341, N602, N614, N655
Epsilon	74	25/4/43/3/0	229	N17, N61, N74, N122, N149, N165, N234, N282, N331, N343, N603, N616, N657
Eta	74	25/8/39/2/0	143	N17, N61, N72, N120, N146, N162, N231, N279, N328, N340, N600, N613, N654
Iota	98	28/9/51/9/1	288	N17, N61, N74, N122, N149, N165, N234, N282, N331, N343, N603, N616, N657
Kappa	105	31/8/55/10/1	306	N17, N61, N74, N122, N149, N165, N234, N281, N331, N343, N603, N616, N657
Lambda	73	25/6/36/6/0	147	N17, N61, N74, N122, N146, N165, N229, N279, N326, N338, N598, N611, N652
Mu	76	26/7/38/5/0	136	N17, N61, N74, N122, N149, N165, N234, N282, N331, N343, N603, N616, N657
Omicron	91	27/9/48/6/1	337	N17, N61, N72, N120, N147, N163, N235, N283, N332, N344, N604, N617, N658

^1^ F = Fucose, ^2^ S = Sialylated, ^3^ SF = Sialofucosylated, ^4^ N = N-Acetylglucosamine, ^5^ M = Mannose, ^6^ Theoretical glycosylation sites adjusted according to the mutations described in Table 1, N = Asparagine.

## Data Availability

The mass spectrometry data generated during the study have been deposited in the GlycoPOST public database with accession number GPST000344.

## Supplementary Materials

The following supporting information can be downloaded at: https://www.mdpi.com/article/10.3390/biom13091421/s1, Figure S1: Protein percent coverage. SARS-CoV-2 Spike S1 protein variants; Figure S2: Total *N*-glycan identifications and their relative abundance. SARS-CoV-2 S1 protein variants; Figure S3: Bar plots of the total *N*-glycan abundance (peak area). SARS-CoV-2 S1 protein variants; Figure S4: PCA of the *N*-glycan relative abundance considering the observed isomeric structures; Figure S5. Bar plots of the relative abundance by *N*-glycan type. Figure S6: Bar plot comparison of the relative abundance of (a) mono-sialylated, (b) tri-sialylated, (c) di-sialylated, and (d) tetra-sialylated *N*-glycan types; Figure S7: Heat maps of the relative abundance of (a) mono-sialylated, (b) di-sialylated, (c) tri-sialylated, and (d) tetra-sialylated N-glycan types. SARS-CoV-2 S1 protein variants; Figure S8: Top ten *N*-glycans, relative abundance. SARS-CoV-2 S1 protein variants of interest; Figure S9: EICs showing the isomeric expression of the *N*-glycans (a) GlcNAc_4_, Hex_5_, Fuc, NeuAc; (b) GlcNAc_5_, Hex_6_, Fuc, NeuAc; and (c) GlcNAc_4_, Hex_5_, Fuc, NeuAc_2_ across the analyzed SARS-CoV-2 S1 protein variants; Table S1: Relative abundance of the identified *N*-glycans; Table S2: Relative standard deviation (%RSD) of the relative abundance of the identified *N*-glycans; Table S3: Relative abundance of the identified isomeric *N*-glycans.
