# Supplementary material for: N-Glycome Profile of the Spike Protein S1: Systemic and Comparative Analysis from Eleven Variants of SARS-CoV-2
Source: Biomolecules. 2023 Sep 20;13(9):1421. doi: 10.3390/biom13091421 (PMC10526240; doi:10.3390/biom13091421)
Supplement: Supplementary file 1 [file biomolecules-13-01421-s001.zip › biomolecules-2535530-supplementary.pdf]

[Supplementary data]

N-Glycome profile of the spike protein S1; systemic and comparative analysis from eleven variants of SARS-CoV-2

Cristian D. Gutierrez Reyes[1‡], Sherifdeen Onigbinde[1‡], Akeem Sanni[1], Andrew I. Bennett[1], Oluwatosin Daramola[1], Parisa Ahmadi[1], Mojibola Fowowe[1], Mojgan Atashi[1], Peilin Jiang[1], Vishal Sandilya[1], Md Abdul Hakim[1], and Yehia Mechref[1*]

1. Department of Chemistry and Biochemistry, Texas Tech University, Lubbock, TX, USA.

‡ These authors contribute equally

*Corresponding author
Department of Chemistry and Biochemistry
Texas Tech University
Lubbock, TX 79409-1061
Email: yehia.mechref@ttu.edu
Tel: 806-742-3059
Fax: 806-742-1289

Table of Contents:

Supplementary Figures:

Supplementary Figure S1. Protein percent coverage. SARS-CoV-2 Spike S1 protein variants: H12 Alpha, H15 Beta, H14 Gamma, H23 Delta, H17 Epsilon, H29 Eta, H28 Iota, H1B Kappa, H32 Lambda, H38 Mu, and H41 Omicron.

Supplementary Figure S2. Total *N*-glycan identifications and their relative abundance. SARS-CoV-2 S1 protein variants: H12 Alpha, H15 Beta, H14 Gamma, H23 Delta, H17 Epsilon, H29 Eta, H28 Iota, H1B Kappa, H32 Lambda, H38 Mu, and H41 Omicron.

Supplementary Figure S3. Bar plots of the total *N*-glycan abundance (peak area). SARS-CoV-2 S1 protein variants: H12 Alpha, H15 Beta, H14 Gamma, H23 Delta, H17 Epsilon, H29 Eta, H28 Iota, H1B Kappa, H32 Lambda, H38 Mu, and H41 Omicron.

Supplementary Figure S4. Principal Component Analysis (PCA) of the *N*-glycan relative abundance considering the observed isomeric structures. SARS-CoV-2 S1 protein variants: H12 Alpha, H15 Beta, H14 Gamma, H23 Delta, H17 Epsilon, H29 Eta, H28 Iota, H1B Kappa, H32 Lambda, H38 Mu, and H41 Omicron.

Supplementary Figure S5. Bar graphs of the relative abundance by type of *N*-glycans across the SARS-CoV-2 S1 protein variants: H12, H14, H15, H17, H23, H28, H29, H32, H38, H41, and H1B. a) fucosylated, b) sialylated, c) sialofucosylated, d) high mannose, and e) other *N*-glycan types.

Supplementary Figure S6. Bar plot comparison of the relative abundance of **a)** mono-sialylated, **b)** tri-sialylated, **c)** di-sialylated, and **d)** and tetra-sialylated *N*-glycan types. SARS-CoV-2 S1 protein variants: H12 Alpha, H15 Beta, H14 Gamma, H23 Delta, H17 Epsilon, H29 Eta, H28 Iota, H1B Kappa, H32 Lambda, H38 Mu, and H41 Omicron.

Supplementary Figure S7. Heat maps of the relative abundance of **a)** mono-sialylated, **b)** di-sialylated, **c)** tri-sialylated, and **d)** tetra-sialylated *N*-glycan types. SARS-CoV-2 S1 protein variants: H12 Alpha, H15 Beta, H14 Gamma, H23 Delta, H17 Epsilon, H29 Eta, H28 Iota, H1B Kappa, H32 Lambda, H38 Mu, and H41 Omicron. The glycan nomenclature is described in **Figure 1** of the main manuscript.

Supplementary Figure S8. Top ten *N*-glycans, relative abundance. SARS-CoV-2 S1 protein variants of interest: H17 Epsilon, H29 Eta, H28 Iota, H1B Kappa, H32 Lambda, and H38 Mu. The glycan nomenclature is described in **Figure 1** of the main manuscript.

Supplementary Figure S9. Extracted ion chromatograms (EICs) showing the isomeric expressions of the *N*-glycans **a)** $GlcNAc_4$, Hex_5, Fuc, NeuAc; **b)** $GlcNAc_5$, Hex_6, Fuc, NeuAc; and **c)** $GlcNAc_4$, Hex_5, Fuc, $Neu5Ac_2$ across the analyzed SARS-CoV2 S1 protein variants (H12 Alpha, H15 Beta, H14 Gamma, H23 Delta, H17 Epsilon, H29 Eta, H28 Iota, H1B Kappa, H32 Lambda, H38 Mu, and H41 Omicron). The stars (*) show the identified isoforms and the *N*-glycan nomenclature is described in **Figure 1** of the main manuscript.

Supplementary Tables:

Supplementary Table S1. Relative abundance of the identified *N*-glycans. A four-digit *N*-glycan nomenclature was used in the following order: *N*-acetylglucosamine, Hexose, Fucose, *N*-acetylneuraminic acid (GlcNAc,Hex,Fuc,NeuAc). ND = Not detected.

Supplementary Table S2. Relative standard deviation (%RSD) of the relative abundance of the identified *N*-glycans. A four-digit *N*-glycan nomenclature was used in the following order: *N*-acetylglucosamine, Hexose, Fucose, *N*-acetylneuraminic acid (GlcNAc,Hex,Fuc,NeuAc).

Supplementary Table S3. Relative abundance of the identified isomeric *N*-glycans. A four-digit *N*-glycan nomenclature was used in the following order: *N*-acetylglucosamine, Hexose, Fucose, *N*-acetylneuraminic acid (GlcNAc,Hex,Fuc,NeuAc), and I = isomer.

Supplementary Figure S1. Protein percent coverage. SARS-CoV-2 Spike S1 protein variants: H12 Alpha, H15 Beta, H14 Gamma, H23 Delta, H17 Epsilon, H29 Eta, H28 Iota, H1B Kappa, H32 Lambda, H38 Mu, and H41 Omicron.

```
        1                                                                                                       100
Alpha   MFVFLVLLPL VSSQCVNLTT RTQLPPAYTN SFTRGVYYPD KVFRSSVLHS TQDLFLPFFS NVTWFHAI-- SGTNGTKRFD NPVLPFNDGV YFASTEKSNI
Beta    MFVFLVLLPL VSSQCVNFTT RTQLPPAYTN SFTRGVYYPD KVFRSSVLHS TQDLFLPFFS NVTWFHAIHV SGTNGTKRFA NPVLPFNDGV YFASTEKSNI
Gamma   MFVFLVLLPL VSSQCVNFTN RTQLPSAYTN SFTRGVYYPD KVFRSSVLHS TQDLFLPFFS NVTWFHAIHV SGTNGTKRFD NPVLPFNDGV YFASTEKSNI
Delta   MFVFLVLLPL VSSQCVNLRT RTQLPPAYTN SFTRGVYYPD KVFRSSVLHS TQDLFLPFFS NVTWFHAIHV SGTNGTKRFD NPVLPFNDGV YFASTEKSNI
Epsilon MFVFLVLLPL VSSQCVNLTT RTQLPPAYTN SFTRGVYYPD KVFRSSVLHS TQDLFLPFFS NVTWFHAIHV SGTNGTKRFD NPVLPFNDGV YFASTEKSNI
Kappa   MFVFLVLLPL VSSQCVNLTT RTQLPPAYTN SFTRGVYYPD KVFRSSVLHS TQDLFLPFFS NVTWFHAIHV SGTNGTKRFD NPVLPFNDGV YFASIEKSNI
Iota    MFVFFVLLPL VSSQCVNLTT RTQLPPAYTN SFTRGVYYPD KVFRSSVLHS TQDLFLPFFS NVTWFHAIHV SGTNGTKRFD NPVLPFNDGV YFASIEKSNI
Eta     MFVFLVLLPL VSSQCVNLTT RTQLPPAYTN SFTRGVYYPD KVFRSSVLHS TRDLFLPFFS NVTWFHVI-- SGTNGTKRFD NPVLPFNDGV YFASTEKSNI
Lambda  MFVFLVLLPL VSSQCVNLTT RTQLPPAYTN SFTRGVYYPD KVFRSSVLHS TQDLFLPFFS NVTWFHAIHV SGTNVIKRFD NPVLPFNDGV YFASTEKSNI
Mu      MFVFLVLLPL VSSQCVNLTT RTQLPPAYTN SFTRGVYYPD KVFRSSVLHS TQDLFLPFFS NVTWFHAIHV SGTNGTKRFD NPVLPFNDGV YFASIEKSNI
Omicron MFVFLVLLPL VSSQCVNLTT RTQLPPAYTN SFTRGVYYPD KVFRSSVLHS TQDLFLPFFS NVTWFHVI-- SGTNGTKRFD NPVLPFNDGV YFASIEKSNI

        101                                                                                                     200
Alpha   IRGWIFGTTL GSKTQSLLIV NNATNVVIKV CEFQFCNDPF LGV-YHKNNK SWMESEFRVY SSANNCTFEY VSQPFLMDLE GKQGNFKNLR EFVFKNIDGY
Beta    IRGWIFGTTL GSKTQSLLIV NNATNVVIKV CEFQFCNDPF LGVYYHKNNK SWMESEFRVY SSANNCTFEY VSQPFLMDLE GKQGNFKNLR EFVFKNIDGY
Gamma   IRGWIFGTTL GSKTQSLLIV NNATNVVIKV CEFQFCNYPF LGVYYHKNNK SWMESEFRVY SSANNCTFEY VSQPFLMDLE GKQGNFKNLS EFVFKNIDGY
Delta   IRGWIFGTTL GSKTQSLLIV NNATNVVIKV CEFQFCNDPF LDVYYHKNNK SWMESG--VY SSANNCTFEY VSQPFLMDLE GKQGNFKNLR EFVFKNIDGY
Epsilon IRGWIFGTTL GSKTQSLLIV NNATNVVIKV CEFQFCNDPF LGVYYHKNNK SCMESEFRVY SSANNCTFEY VSQPFLMDLE GKQGNFKNLR EFVFKNIDGY
Kappa   IRGWIFGTTL DSKTQSLLIV NNATNVVIKV CEFQFCNDPF LDVYYHKNNK SWMKSEFRVY SSANNCTFEY VSQPFLMDLE GKQGNFKNLR EFVFKNIDGY
Iota    IRGWIFGTTL GSKTQSLLIV NNATNVVIKV CEFQFCNDPF LGVYYHKNNK SWMESEFRVY SSANNCTFEY VSQPFLMDLE GKQGNFKNLR EFVFKNIDGY
Eta     IRGWIFGTTL GSKTQSLLIV NNATNVVIKV CEFQFCNDPF LGV-YHKNNK SWMESEFRVY SSANNCTFEY VSQPFLMDLE GKQGNFKNLR EFVFKNIDGY
Lambda  IRGWIFGTTL GSKTQSLLIV NNATNVVIKV CEFQFCNDPF LGVYYHKNNK SWMESEFRVY SSANNCTFEY VSQPFLMDLE GKQGNFKNLR EFVFKNIDGY
Mu      IRGWIFGTTL GSKTQSLLIV NNATNVVIKV CEFQFCNDPF LGVSNHKNNK SWMESEFRVY SSANNCTFEY VSQPFLMDLE GKQGNFKNLR EFVFKNIDGY
Omicron IRGWIFGTTL GSKTQSLLIV NNATNVVIKV CEFQFCNDPF LDVYYHKNNK SWMESEFRVY SSANNCTFEY VSQPFLMDLE GKQGNFKNLR EFVFKNIDGY

        201                                                                                                     300
Alpha   FKIYSKHTPI NLVRDLPQGF SALEPLVDLP IGINITRFQT LLALHRSYLT PGDSSSGWTA GAAAYYVGYL QPRTFLLKYN ENGTITDAVD CALDPLSETK
Beta    FKIYSKHTPI NLVRGLPQGF SALEPLVDLP IGINITRFQT L---HISYLT PGDSSSGWTA GAAAYYVGYL QPRTFLLKYN ENGTITDAVD CALDPLSETK
Gamma   FKIYSKHTPI NLVRDLPQGF SALEPLVDLP IGINITRFQT LLALHRSYLT PGDSSSGWTA GAAAYYVGYL QPRTFLLKYN ENGTITDAVD CALDPLSETK
Delta   FKIYSKHTPI NLVRDLPQGF SALEPLVDLP IGINITRFQT LLALHRSYLT PGDSSSGWTA GAAAYYVGYL QPRTFLLKYN ENGTITDAVD CALDPLSETK
Epsilon FKIYSKHTPI NLVRDLPQGF SALEPLVDLP IGINITRFQT LLALHRSYLT PGDSSSGWTA GAAAYYVGYL QPRTFLLKYN ENGTITDAVD CALDPLSETK
Kappa   FKIYSKHTPI NLVRDLPQGF SALEPLVDLP IGINITRFQT LLALHRSYLT PGDSSSGWTA GAAAYYVGYL QPRTFLLKYN ENGTITDAVD CALDPLSETK
Iota    FKIYSKHTPI NLVRDLPQGF SALEPLVDLP IGINITRFQT LLALHRSYLT PGGSSSGWTA GAAAYYVGYL QPRTFLLKYN ENGTITDAVD CALDPLSETK
Eta     FKIYSKHTPI NLVRDLPQGF SALEPLVDLP IGINITRFQT LLALHRSYLT PGDSSSGWTA GAAAYYVGYL QPRTFLLKYN ENGTITDAVD CALDPLSETK
Lambda  FKIYSKHTPI NLVRDLPQGF SALEPLVDLP IGINITRFQT LLALHR---- --NSSSGWTA GAAAYYVGYL QPRTFLLKYN ENGTITDAVD CALDPLSETK
Mu      FKIYSKHTPI NLVRDLPQGF SALEPLVDLP IGINITRFQT LLALHRSYLT PGDSSSGWTA GAAAYYVGYL QPRTFLLKYN ENGTITDAVD CALDPLSETK
                      EPE
                       ↓
Omicron FKIYSKHTPI NIVRDLPQGF SALEPLVDLP IGINITRFQT LLALHRSYLT PGDSSSGWTA GAAAYYVGYL QPRTFLLKYN ENGTITDAVD CALDPLSETK

        301                                                                                                     400
Alpha   CTLKSFTVEK GIYQTSNFRV QPTESIVRFP NITNLCPFGE VFNATRFASV YAWNRKRISN CVADYSVLYN SASFSTFKCY GVSPTKLNDL CFTNVYADSF
Beta    CTLKSFTVEK GIYQTSNFRV QPTESIVRFP NITNLCPFGE VFNATRFASV YAWNRKRISN CVADYSVLYN SASFSTFKCY GVSPTKLNDL CFTNVYADSF
Gamma   CTLKSFTVEK GIYQTSNFRV QPTESIVRFP NITNLCPFGE VFNATRFASV YAWNRKRISN CVADYSVLYN SASFSTFKCY GVSPTKLNDL CFTNVYADSF
Delta   CTLKSFTVEK GIYQTSNFRV QPTESIVRFP NITNLCPFGE VFNATRFASV YAWNRKRISN CVADYSVLYN SASFSTFKCY GVSPTKLNDL CFTNVYADSF
Epsilon CTLKSFTVEK GIYQTSNFRV QPTESIVRFP NITNLCPFGE VFNATRFASV YAWNRKRISN CVADYSVLYN SASFSTFKCY GVSPTKLNDL CFTNVYADSF
Kappa   CTLKSFTVEK GIYQTSNFRV QPTESIVRFP NITNLCPFGE VFNATRFASV YAWNRKRISN CVADYSVLYN SASFSTFKCY GVSPTKLNDL CFTNVYADSF
Iota    CTLKSFTVEK GIYQTSNFRV QPTESIVRFP NITNLCPFGE VFNATRFASV YAWNRKRISN CVADYSVLYN SASFSTFKCY GVSPTKLNDL CFTNVYADSF
Eta     CTLKSFTVEK GIYQTSNFRV QPTESIVRFP NITNLCPFGE VFNATRFASV YAWNRKRISN CVADYSVLYN SASFSTFKCY GVSPTKLNDL CFTNVYADSF
Lambda  CTLKSFTVEK GIYQTSNFRV QPTESIVRFP NITNLCPFGE VFNATRFASV YAWNRKRISN CVADYSVLYN SASFSTFKCY GVSPTKLNDL CFTNVYADSF
Mu      CTLKSFTVEK GIYQTSNFRV QPTESIVRFP NITNLCPFGE VFNATKFASV YAWNRKRISN CVADYSVLYN SASFSTFKCY GVSPTKLNDL CFTNVYADSF
Omicron CTLKSFTVEK GIYQTSNFRV QPTESIVRFP NITNLCPFDE VFNATRFASV YAWNRKRISN CVADYSVLYN LAPFFTFKCY GVSPTKLNDL CFTNVYADSF

        401                                                                                                     500
Alpha   VIRGDEVRQI APGQTGKIAD YNYKLPDDFT GCVIAWNSNN LDSKVGGNYN YLYRLFRKSN LKPFERDIST EIYQAGSTPC NGVEGFNCYF PLQSYGFQPT
Beta    VIRGDEVRQI APGQTGNIAD YNYKLPDDFT GCVIAWNSNN LDSKVGGNYN YLYRLFRKSN LKPFERDIST EIYQAGSTPC NGVKGFNCYF PLQSYGFQPT
Gamma   VIRGDEVRQI APGQTGTIAD YNYKLPDDFT GCVIAWNSNN LDSKVGGNYN YLYRLFRKSN LKPFERDIST EIYQAGSTPC NGVKGFNCYF PLQSYGFQPT
Delta   VIRGDEVRQI APGQTGKIAD YNYKLPDDFT GCVIAWNSNN LDSKVGGNYN YRYRLFRKSN LKPFERDIST EIYQAGSKPC NGVEGFNCYF PLQSYGFQPT
Epsilon VIRGDEVRQI APGQTGKIAD YNYKLPDDFT GCVIAWNSNN LDSKVGGNYN YRYRLFRKSN LKPFERDIST EIYQAGSTPC NGVEGFNCYF PLQSYGFQPT
Kappa   VIRGDEVRQI APGQTGKIAD YNYKLPDDFT GCVIAWNSNN LDSKVGGNYN YRYRLFRKSN LKPFERDIST EIYQAGSTPC NGVQGFNCYF PLQSYGFQPT
Iota    VIRGDEVRQI APGQTGKIAD YNYKLPDDFT GCVIAWNSNN LDSKVGGNYN YLYRLFRKSN LKPFERDIST EIYQAGNTPC NGVKGFNCYF PLQSYGFQPT
Eta     VIRGDEVRQI APGQTGKIAD YNYKLPDDFT GCVIAWNSNN LDSKVGGNYN YLYRLFRKSN LKPFERDIST EIYQAGSTPC NGVKGFNCYF PLQSYGFQPT
Lambda  VIRGDEVRQI APGQTGKIAD YNYKLPDDFT GCVIAWNSNN LDSKVGGNYN YQYRLFRKSN LKPFERDIST EIYQAGSTPC NGVEGFNCYS PLQSYGFQPT
Mu      VIRGDEVRQI APGQTGKIAD YNYKLPDDFT GCVIAWNSNN LDSKVGGNYN YLYRLFRKSN LKPFERDIST EIYQAGSTPC NGVKGFNCYF PLQSYGFQPT
Omicron VIRGDEVRQI APGQTGNIAD YNYKLPDDFT GCVIAWNSNK LDSKVSGNYN YLYRLFRKSN LKPFERDIST EIYQAGNKPC NGVAGFNCYF PLRSYSFRPT

        501                                                                                                     600
Alpha   YGVGYQPYRV VVLSFELLHA PATVCGPKKS TNLVKNKCVN FNFNGLTGTG VLTESNKKFL PFQQFGRDID DTTDAVRDPQ TLEILDITPC SFGGVSVITP
Beta    YGVGYQPYRV VVLSFELLHA PATVCGPKKS TNLVKNKCVN FNFNGLTGTG VLTESNKKFL PFQQFGRDIA DTTDAVRDPQ TLEILDITPC SFGGVSVITP
Gamma   YGVGYQPYRV VVLSFELLHA PATVCGPKKS TNLVKNKCVN FNFNGLTGTG VLTESNKKFL PFQQFGRDIA DTTDAVRDPQ TLEILDITPC SFGGVSVITP
Delta   NGVGYQPYRV VVLSFELLHA PATVCGPKKS TNLVKNKCVN FNFNGLTGTG VLTESNKKFL PFQQFGRDIA DTTDAVRDPQ TLEILDITPC SFGGVSVITP
Epsilon NGVGYQPYRV VVLSFELLHA PATVCGPKKS TNLVKNKCVN FNFNGLTGTG VLTESNKKFL PFQQFGRDIA DTTDAVRDPQ TLEILDITPC SFGGVSVITP
Kappa   NGVGYQPYRV VVLSFELLHA PATVCGPKKS TNLVKNKCVN FNFNGLTGTG VLTESNKKFL PFQQFGRDIA DTTDAVRDPQ TLEILDITPC SFGGVSVITP
Iota    NGVGYQPYRV VVLSFELLHA PATVCGPKKS TNLVKNKCVN FNFNGLTGTG VLTESNKKFL PFQQFGRDIA DTTDAVRDPQ TLEILDITPC SFGGVSVITP
Eta     NGVGYQPYRV VVLSFELLHA PATVCGPKKS TNLVKNKCVN FNFNGLTGTG VLTESNKKFL PFQQFGRDIA DTTDAVRDPQ TLEILDITPC SFGGVSVITP
Lambda  NGVGYQPYRV VVLSFELLHA PATVCGPKKS TNLVKNKCVN FNFNGLTGTG VLTESNKKFL PFQQFGRDIA DTTDAVRDPQ TLEILDITPC SFGGVSVITP
Mu      YGVGYQPYRV VVLSFELLHA PATVCGPKKS TNLVKNKCVN FNFNGLTGTG VLTESNKKFL PFQQFGRDIA DTTDAVRDPQ TLEILDITPC SFGGVSVITP
Omicron YGVGHQPYRV VVLSFELLHA PATVCGPKKS TNLVKNKCVN FNFNGLKGTG VLTESNKKFL PFQQFGRDIA DTTDAVRDPQ TLEILDITPC SFGGVSVITP
```

```
        601                                                                                685
Alpha   GTNTSNQVAV  LYQGVNCTEV  PVAIHADQLT  PTWRVYSTGS  NVFQTRAGCL  IGAEHVNNSY  ECDIPIGAGI  CASYQTQTNS  HRRAR
Beta    GTNTSNQVAV  LYQGVNCTEV  PVAIHADQLT  PTWRVYSTGS  NVFQTRAGCL  IGAEHVNNSY  ECDIPIGAGI  CASYQTQTNS  PRRAR
Gamma   GTNTSNQVAV  LYQGVNCTEV  PVAIHADQLT  PTWRVYSTGS  NVFQTRAGCL  IGAEYVNNSY  ECDIPIGAGI  CASYQTQTNS  PRRAR
Delta   GTNTSNQVAV  LYQGVNCTEV  PVAIHADQLT  PTWRVYSTGS  NVFQTRAGCL  IGAEHVNNSY  ECDIPIGAGI  CASYQTQTNS  RRRAR
Epsilon GTNTSNQVAV  LYQGVNCTEV  PVAIHADQLT  PTWRVYSTGS  NVFQTRAGCL  IGAEHVNNSY  ECDIPIGAGI  CASYQTQTNS  PRRAR
Kappa   GTNTSNQVAV  LYQGVNCTEV  PVAIHADQLT  PTWRVYSTGS  NVFQTRAGCL  IGAEHVNNSY  ECDIPIGAGI  CASYQTQTNS  RRRAR
Iota    GTNTSNQVAV  LYQGVNCTEV  PVAIHADQLT  PTWRVYSTGS  NVFQTRAGCL  IGAEHVNNSY  ECDIPIGAGI  CASYQTQTNS  PRRAR
Eta     GTNTSNQVAV  LYQGVNCTEV  PVAIHADQLT  PTWRVYSTGS  NVFQTRAGCL  IGAEHVNNSY  ECDIPIGAGI  CASYQTHTNS  PRRAR
Lambda  GTNTSNQVAV  LYQGVNCTEV  PVAIHADQLT  PTWRVYSTGS  NVFQTRAGCL  IGAEHVNNSY  ECDIPIGAGI  CASYQTQTNS  PRRAR
Mu      GTNTSNQVAV  LYQGVNCTEV  PVAIHADQLT  PTWRVYSTGS  NVFQTRAGCL  IGAEHVNNSY  ECDIPIGAGI  CASYQTQTNS  HRRAR
Omicron GTNTSNQVAV  LYQGVNCTEV  PVAIHADQLT  PTWRVYSTGS  NVFQTRAGCL  IGAEYVNNSY  ECDIPIGAGI  CASYQTQTKS  HRRAR
```

The percentage coverage was determined by the Proteome Discoverer (Thermo Sci.) software, and the results were as follows: Alpha 87%; Beta 97%; Gamma 87%; Delta 89%; Epsilon 87%; Kappa 84%; Iota 86%; Eta 70%, Lambda 88%; Mu 85%; and Omicron 91%.

*Sections highlighted in yellow show the protein percent coverage
*Substituted amino acid residues are colored in red
* "–" Represents deletions
* "↓" Indicates point of insertion

Supplementary Figure S2. Total *N*-glycan identifications and their relative abundance. SARS-CoV-2 S1 protein variants: H12 Alpha, H15 Beta, H14 Gamma, H23 Delta, H17 Epsilon, H29 Eta, H28 Iota, H1B Kappa, H32 Lambda, H38 Mu, and H41 Omicron.

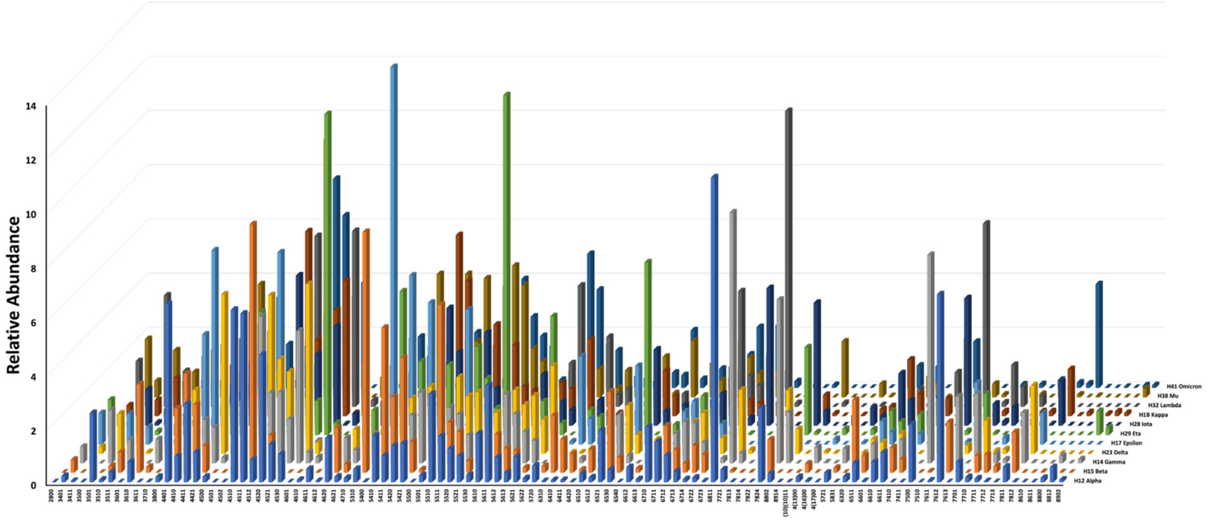

Supplementary Figure S3. Bar plots of the total *N*-glycan abundance (peak area). SARS-CoV-2 S1 protein variants: H12 Alpha, H15 Beta, H14 Gamma, H23 Delta, H17 Epsilon, H29 Eta, H28 Iota, H1B Kappa, H32 Lambda, H38 Mu, and H41 Omicron.

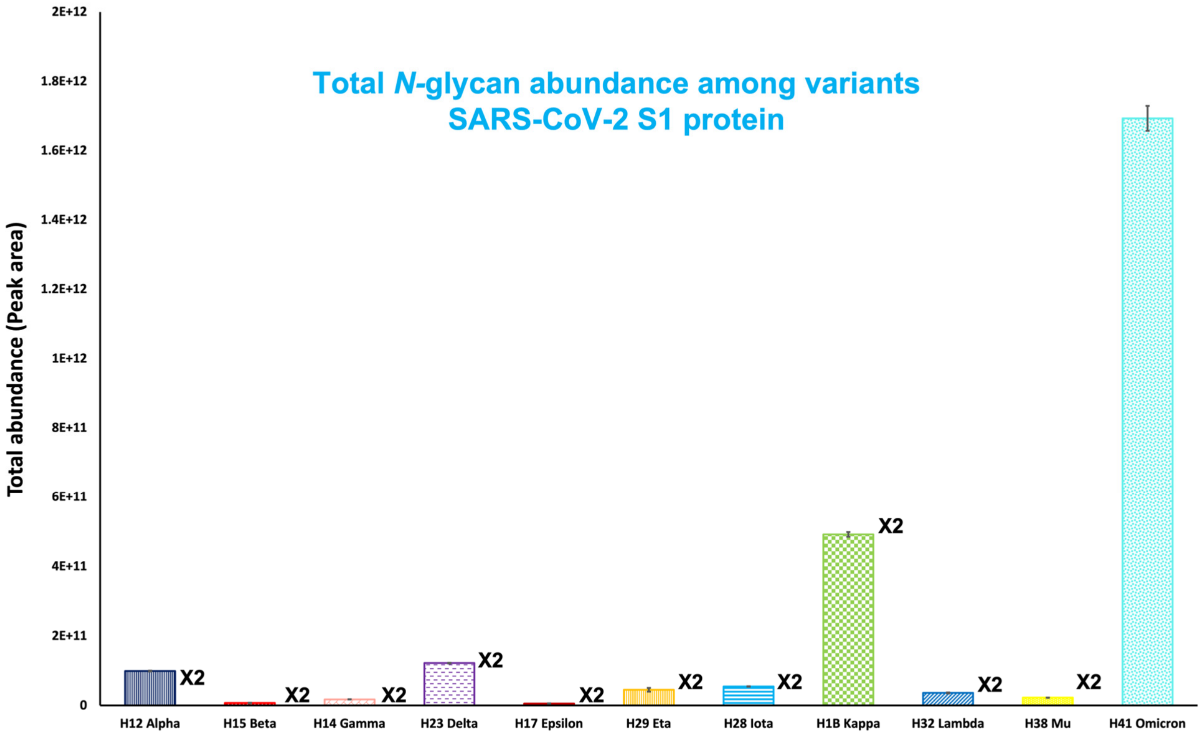

X2: These data were multiplied by two for better visualization.

Supplementary Figure S4. Principal Component Analysis (PCA) of the *N*-glycan relative abundance considering the observed isomeric structures. SARS-CoV-2 S1 protein variants: H12 Alpha, H15 Beta, H14 Gamma, H23 Delta, H17 Epsilon, H29 Eta, H28 Iota, H1B Kappa, H32 Lambda, H38 Mu, and H41 Omicron.

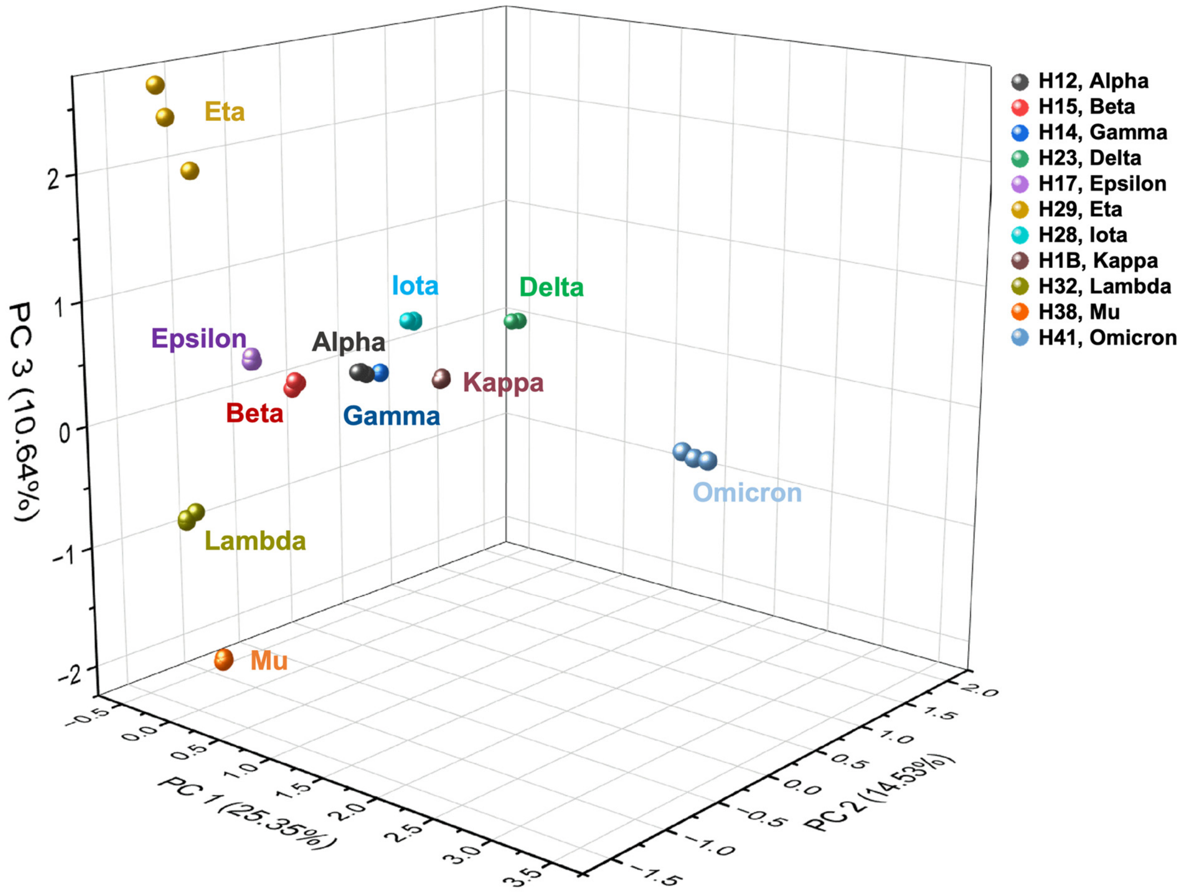

Supplementary Figure S5. Bar graphs of the relative abundance by type of *N*-glycans across the SARS-CoV-2 S1 protein variants: H12, H14, H15, H17, H23, H28, H29, H32, H38, H41, and H1B. **a)** Fucosylated, **b)** Sialylated, **c)** Sialofucosylated, **d)** High mannose, and **e)** Other *N*-glycan types.

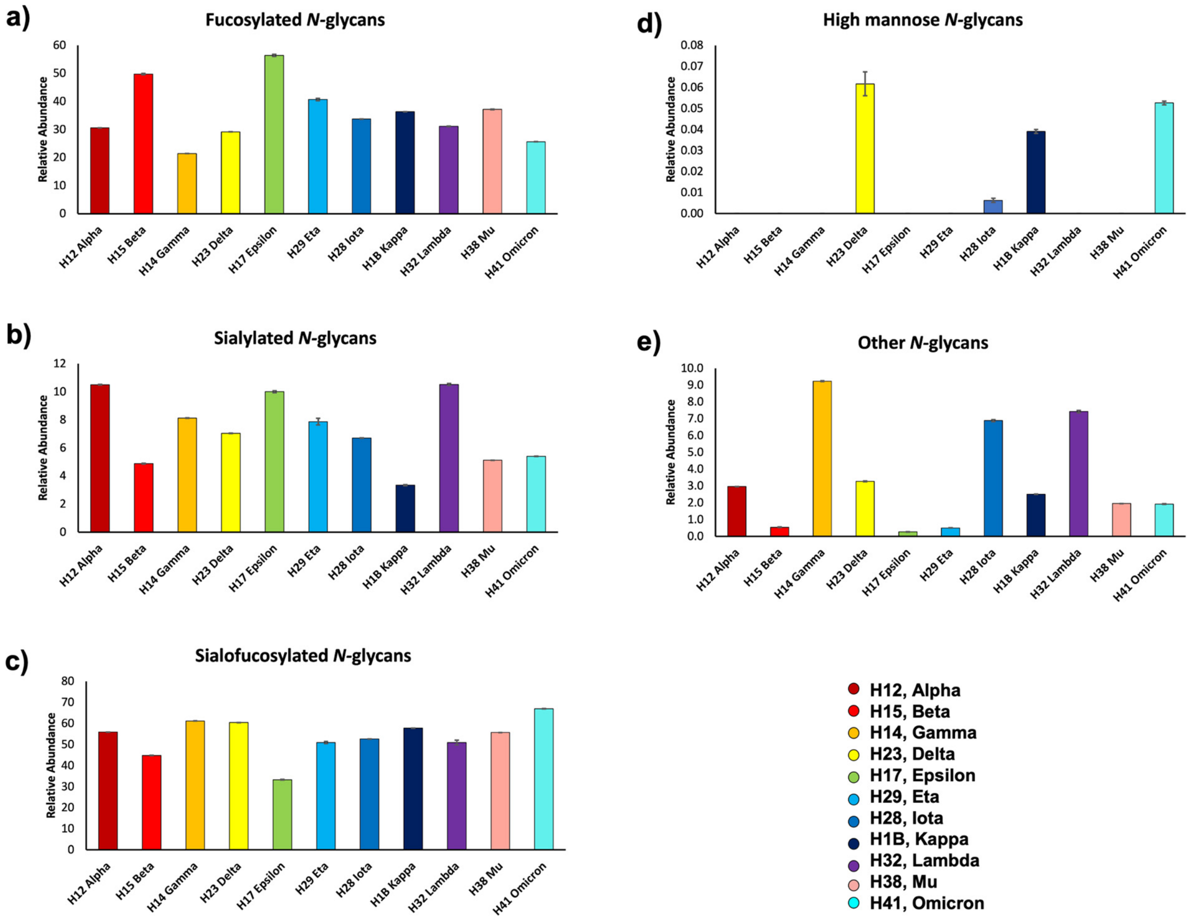

Supplementary Figure S6. Bar plot comparison of the relative abundance of **a)** mono-sialylated, **b)** tri-sialylated, **c)** di-sialylated, and **d)** tetra-sialylated *N*-glycan types. SARS-CoV-2 S1 protein variants: H12 Alpha, H15 Beta, H14 Gamma, H23 Delta, H17 Epsilon, H29 Eta, H28 Iota, H1B Kappa, H32 Lambda, H38 Mu, and H41 Omicron.

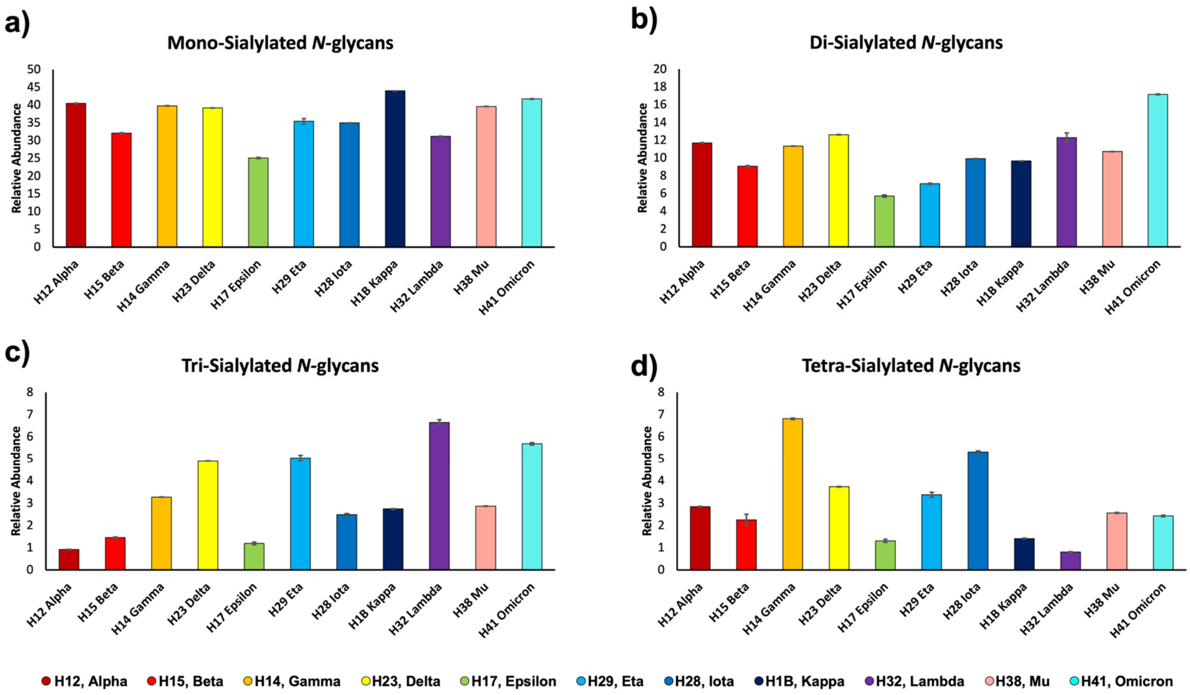

Supplementary Figure S7. Heat maps of the relative abundance of **a)** mono-sialylated, **b)** di-sialylated, **c)** tri-sialylated, and **d)** tetra-sialylated *N*-glycan types. SARS-CoV-2 S1 protein variants: H12 Alpha, H15 Beta, H14 Gamma, H23 Delta, H17 Epsilon, H29 Eta, H28 Iota, H1B Kappa, H32 Lambda, H38 Mu, and H41 Omicron. The glycan nomenclature is described in **Figure 1** of the main manuscript.

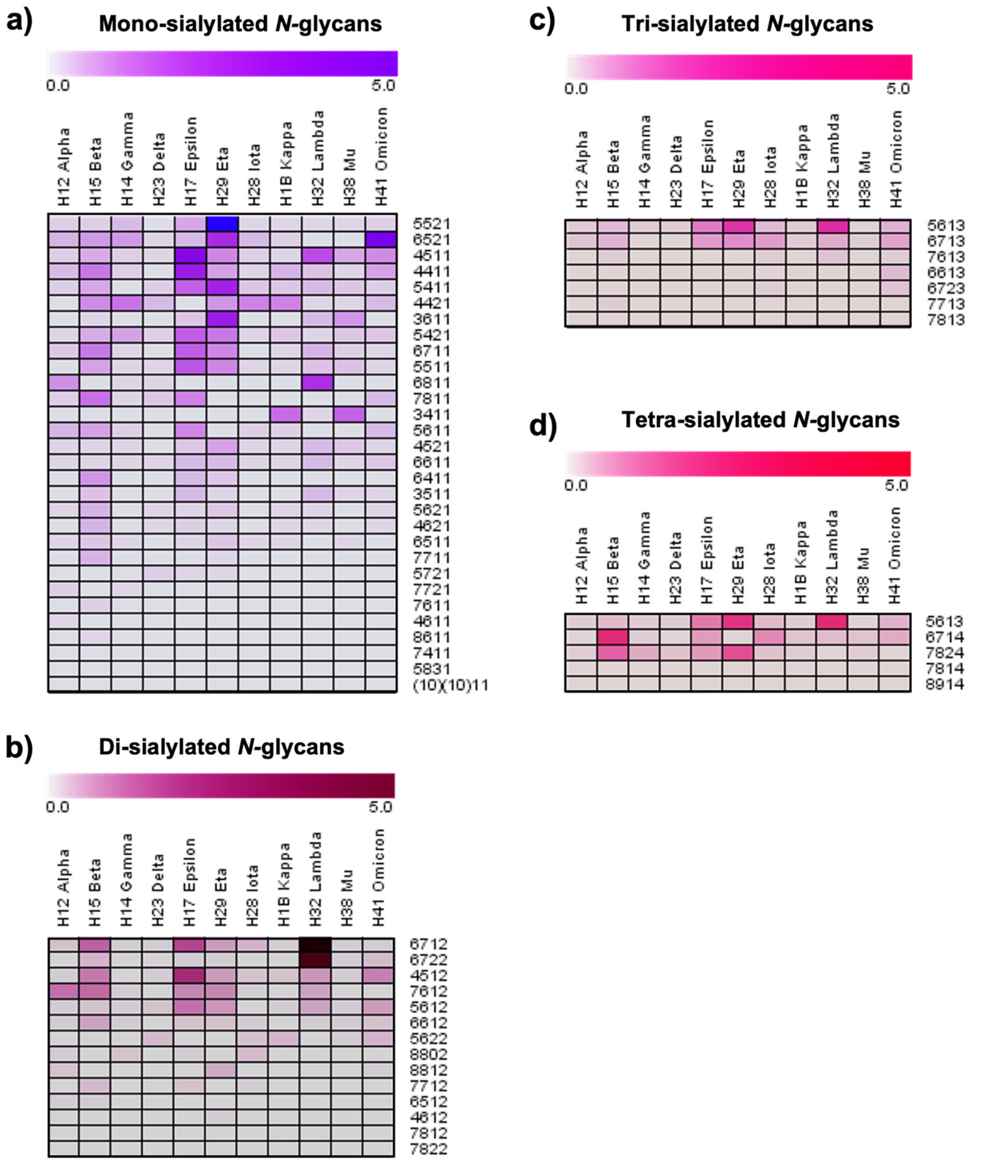

Supplementary Figure S8. Top ten *N*-glycans, relative abundance. SARS-CoV-2 S1 protein variants of interest: H17 Epsilon, H29 Eta, H28 Iota, H1B Kappa, H32 Lambda, and H38 Mu. The glycan nomenclature is described in **Figure 1** of the main manuscript.

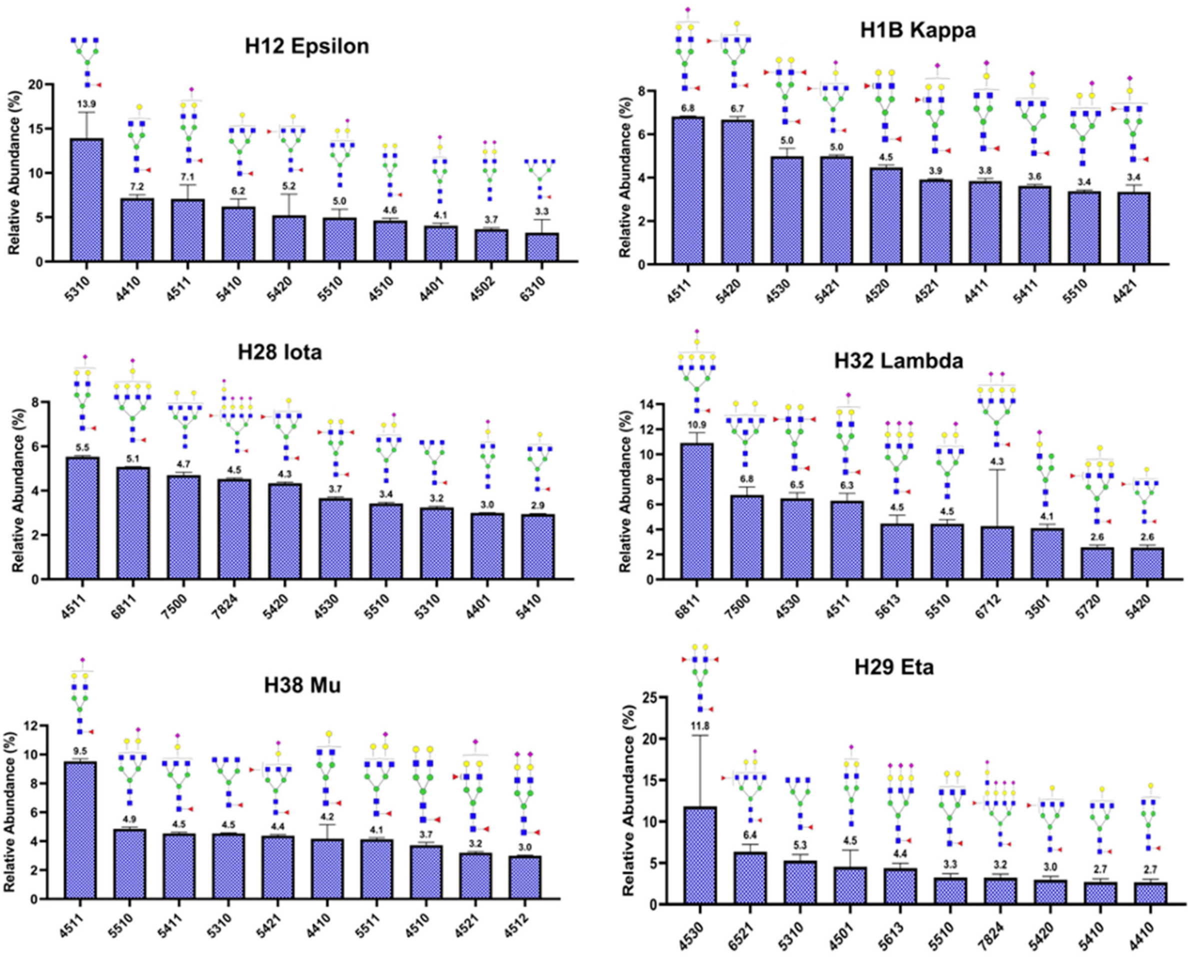

Supplementary Figure S9. Extracted ion chromatograms (EICs) showing the isomeric expressions of the *N*-glycans **a)** GlcNAc$_4$, Hex$_5$, Fuc, NeuAc; **b)** GlcNAc$_5$, Hex$_6$, Fuc, NeuAc; and **c)** GlcNAc$_4$, Hex$_5$, Fuc, Neu5Ac$_2$ across the analyzed SARS-CoV2 S1 protein variants (H12 Alpha, H15 Beta, H14 Gamma, H23 Delta, H17 Epsilon, H29 Eta, H28 Iota, H1B Kappa, H32 Lambda, H38 Mu, and H41 Omicron). The stars (*) show the identified isoforms and the *N*-glycan nomenclature is described in **Figure 1** of the main manuscript.

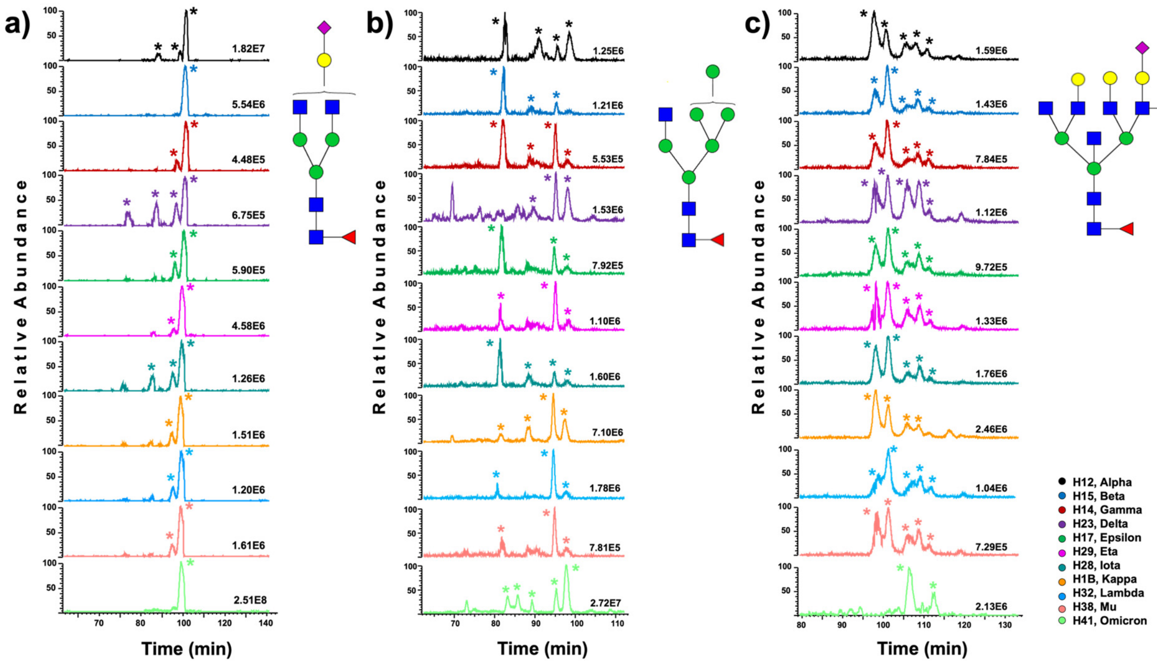

Supplementary Table S1. Relative abundance of the identified *N*-glycans. A four-digit *N*-glycan nomenclature was used in the following order: *N*-acetylglucosamine, Hexose, Fucose, *N*-acetylneuraminic acid (GlcNAc,Hex,Fuc,NeuAc). ND = Not detected.

Glycan	H12 Alpha	H15 Beta	H14 Gamma	H23 Delta	H17 Epsilon	H29 Eta	H28 Iota	H1B Kappa	H32 Lambda	H38 Mu	H41 Omicron
2-8-0-0	ND	ND	ND	0.0617	ND	ND	0.0063	0.0391	ND	ND	0.0526
3-4-0-1	0.2299	0.4788	0.6079	0.4841	1.1581	1.3016	0.4166	0.3977	1.6788	2.1475	0.1064
3-4-1-1	0.0477	ND	0.2616	0.2623	ND	ND	0.1221	0.4624	0.4736	0.6042	ND
3-5-0-0	0.0015	0.0406	ND	0.0363	ND	ND	0.0017	0.0061	0.0008	0.0061	0.0298
3-5-0-1	2.5531	ND	1.9559	1.4901	1.1344	0.7449	1.3393	0.5982	4.1059	1.7300	0.2440
3-5-1-0	0.0939	0.2564	0.1934	0.1569	0.4144	0.2596	0.1099	0.2442	0.5223	0.5281	0.0961
3-5-1-1	0.3666	0.7604	0.8614	0.7498	0.6423	0.1446	0.6340	1.3998	1.3121	0.9291	0.2730
3-6-0-1	ND	0.0072	ND	0.0667	ND	ND	0.0323	0.0866	0.4366	ND	0.0594
3-6-1-0	0.7473	3.2649	ND	0.3683	0.7213	0.3827	0.2680	0.7916	1.8581	1.7463	0.1440
3-6-1-1	ND	0.2199	0.8874	0.3543	0.1755	0.7962	0.2102	0.7287	1.0262	0.6380	ND
3-7-1-0	0.0077	ND	ND	0.0329	ND	ND	ND	0.0099	0.0409	ND	0.0086
3-8-0-0	0.1991	0.0635	0.2678	0.3190	0.0460	0.2828	0.2729	0.2993	0.4099	0.9186	ND
4-4-0-1	6.5741	2.3768	1.6423	2.3503	4.0557	0.1719	2.9947	1.8961	2.5116	0.4972	2.3519
4-4-1-0	0.9551	3.6607	0.4229	0.5189	7.1577	2.6718	1.4807	0.1955	1.7392	4.1703	0.9280
4-4-1-1	2.8835	2.5189	1.6028	0.4713	2.5605	1.1569	1.2283	3.8431	1.0816	2.4430	3.3495
4-4-2-1	1.1090	0.9992	1.3161	5.8819	ND	1.2653	2.2065	3.3516	0.4665	0.9321	1.6052
4-5-0-0	0.2058	ND	0.2086	0.3921	ND	ND	0.7009	0.3736	ND	ND	0.2924
4-5-0-1	ND	ND	ND	0.1353	ND	4.5445	0.0734	ND	ND	ND	1.5620
4-5-0-2	ND	0.2477	ND	0.5827	3.6608	0.3710	ND	ND	ND	0.2451	0.8502
4-5-1-0	6.3614	2.5607	1.3191	2.3319	4.6439	2.4542	2.0160	1.6817	2.4571	3.7224	2.7125
4-5-1-1	6.2205	9.1681	5.4206	5.8536	7.0832	1.6232	5.5383	6.8219	6.2980	9.5262	7.7136
4-5-1-2	0.8239	4.1081	2.5986	3.4961	1.1253	1.3849	2.2885	2.7976	2.2316	2.9952	6.3611
4-5-2-0	4.7067	1.3900	2.6201	3.0416	1.1884	1.3475	2.6252	4.4703	0.5070	1.9495	3.3856
4-5-2-1	1.3996	0.5315	1.6360	3.5830	0.5710	1.2603	1.3061	3.9145	1.4881	3.2060	3.8951
4-5-3-0	1.0300	ND	4.8875	6.2682	0.1318	11.8384	3.6630	4.9933	6.4852	0.9384	ND
4-6-0-1	ND	ND	0.4110	0.4170	ND	0.4479	0.0525	0.1134	1.6165	ND	0.0939
4-6-1-0	0.0844	ND	0.2343	0.1985	ND	ND	0.4345	0.4990	0.2447	0.7419	ND
4-6-1-1	0.4929	0.0090	0.0917	ND	ND	0.0469	ND	0.0703	0.1027	0.1264	0.1366
4-6-1-2	0.0516	ND	0.0986	0.0615	ND	0.1926	0.0580	0.0759	ND	ND	0.0732
4-6-2-0	1.6313	1.6592	0.9416	0.9077	1.1638	0.9144	0.9068	1.8812	ND	0.9099	1.8857
4-6-2-1	0.2073	0.3156	0.4972	0.6367	0.1516	0.2209	0.4440	0.8121	0.3954	0.2273	1.1764
4-7-1-0	0.1623	ND	0.0781	0.7451	ND	ND	0.0905	0.0328	ND	0.5302	ND
5-3-1-0	0.4925	8.8851	1.0789	0.3587	13.9232	5.3036	3.2410	0.0929	0.4980	4.5446	0.9509
5-4-0-0	0.0184	ND	0.1017	0.0549	0.0256	ND	0.0737	0.2825	0.1452	ND	0.1768
5-4-1-0	1.7173	5.3514	1.4752	1.6790	6.2321	2.7167	2.9411	2.2947	2.2700	ND	1.7633
5-4-1-1	0.9820	2.7945	1.2079	2.0561	1.8771	1.4191	2.2819	3.6266	1.1006	4.5482	2.0432
5-4-2-0	1.3428	4.2368	1.7786	2.0961	5.2275	2.9894	4.3372	6.6787	2.5502	2.0527	2.0000
5-4-2-1	1.4328	1.1543	2.6106	2.4825	0.9709	2.5928	2.6983	4.9893	0.4023	4.3875	1.5896
5-5-0-0	ND	0.1255	0.0639	0.1479	ND	ND	0.0915	0.4951	0.0614	ND	0.3723
5-5-0-1	0.2643	1.7665	0.5206	0.1011	ND	ND	1.0570	ND	ND	0.0521	ND
5-5-1-0	3.2554	6.1993	2.0553	2.8380	4.9791	3.2534	3.4223	3.3755	4.4536	4.8535	4.0122
5-5-1-1	1.6965	1.8607	1.8130	2.1293	1.6729	2.0861	1.3615	2.7487	2.2238	4.1354	2.6293
5-5-2-0	1.2347	1.5092	1.0399	2.2737	0.3439	0.9931	1.0827	2.6276	0.3792	1.8023	1.9090
5-5-2-1	0.9682	0.4376	1.0917	1.5171	0.5440	0.8806	0.7283	1.0956	0.2796	1.3234	1.5475
5-5-3-0	0.2760	0.0688	0.2206	1.5202	0.0995	ND	0.2372	0.9523	ND	0.5112	0.2957
5-6-1-0	1.7896	0.8443	0.4751	0.4966	2.0175	0.4982	0.3716	0.4964	0.5957	0.4408	0.6259
5-6-1-1	3.5747	1.4120	2.5426	2.3508	1.1296	ND	1.8801	2.1127	ND	ND	3.7673
5-6-1-2	0.9141	0.9068	1.8252	1.8196	0.8833	1.2450	1.3774	1.2253	1.6031	2.5308	4.9448
5-6-1-3	0.3453	0.8170	1.1705	2.1483	0.6702	4.3818	0.8692	1.0098	4.4683	0.7095	3.6221
5-6-2-1	0.9380	0.1968	0.8382	1.2689	0.1989	0.4396	0.5737	0.9169	0.4826	1.0383	1.6193
5-6-2-2	0.1510	ND	ND	3.2481	ND	ND	2.1382	2.8365	ND	1.3069	1.3835
5-7-2-0	0.6174	0.2487	0.3318	0.4740	0.1111	0.1183	0.2995	0.3474	2.5767	0.3473	0.4795
6-3-1-0	0.0423	2.1178	0.0176	0.0532	3.2515	0.9273	0.9405	0.1494	0.4444	0.9956	0.4333
6-4-1-0	0.0808	1.2441	0.1113	0.0502	0.9483	0.6777	0.5900	0.1121	0.6561	0.3165	0.3589
6-4-1-1	0.0807	0.6908	0.1835	0.1953	0.2908	0.2970	0.3914	0.3413	ND	0.2927	0.2502
6-4-2-0	0.0169	0.1159	ND	0.0479	0.1022	0.0831	0.1117	0.0710	0.1930	0.0720	0.0499
6-5-1-0	0.3207	0.8601	0.2025	0.2128	0.6714	0.2530	0.5702	0.2503	0.7264	1.4942	0.5595
6-5-1-2	0.1778	0.2024	ND	0.1838	0.2112	0.0677	0.2781	0.2025	0.1705	0.1491	0.4712
6-5-2-1	1.9089	2.9908	1.7853	1.7890	2.8949	6.3706	2.8052	1.6716	ND	ND	2.1226

Supplementary Table S1. Continued…

Glycan	H12 Alpha	H15 Beta	H14 Gamma	H23 Delta	H17 Epsilon	H29 Eta	H28 Iota	H1B Kappa	H32 Lambda	H38 Mu	H41 Omicron
6-5-3-0	0.4619	0.5656	0.2841	0.7095	0.6037	ND	0.5319	0.7359	0.4701	2.0806	0.3286
6-5-4-0	ND	0.0164	ND	ND	ND	0.3511	ND	0.1573	0.0866	ND	0.1316
6-6-1-2	0.5539	0.1647	0.3410	0.5305	0.1506	0.3810	0.7582	0.4387	0.3284	1.2529	0.7048
6-6-1-3	0.1121	ND	0.3866	0.4492	0.0810	0.1064	0.2153	0.3516	ND	0.6201	0.3623
6-7-1-0	2.0387	0.8253	0.5272	0.2769	1.2184	0.7097	0.6030	0.3067	ND	0.6870	0.6301
6-7-1-1	1.4994	1.7377	1.0941	0.5972	1.5921	1.4461	0.9671	0.5228	1.4728	2.0744	1.2331
6-7-1-2	0.9869	0.8472	1.5482	1.2122	0.8106	1.0261	1.1715	0.8924	4.2618	1.4572	2.2442
6-7-1-3	0.4054	0.3446	1.4870	1.5104	0.3369	0.4344	1.0170	0.8294	1.1479	0.8879	1.1462
6-7-1-4	0.1103	1.0133	0.7714	1.3468	0.7550	0.1415	0.7721	0.5966	0.7899	0.4929	2.3137
6-7-2-2	0.1681	0.6691	0.4126	0.5891	0.0691	0.2420	0.3014	0.4885	2.0110	0.9854	0.6412
6-7-2-3	0.0368	0.0486	0.1766	0.4960	0.0303	0.0484	0.1431	0.2781	0.1940	0.1629	0.2467
6-8-1-1	11.2475	ND	9.2569	2.3647	ND	ND	5.0740	0.4175	10.9139	ND	ND
7-7-2-1	0.4702	0.0788	0.3780	0.1504	0.0658	ND	0.0703	0.2060	0.1354	0.0691	0.1540
7-8-1-3	0.0101	0.0070	ND	0.0443	ND	ND	0.0210	0.0441	0.0363	ND	0.0074
7-8-1-4	ND	0.0107	ND	0.0306	ND	ND	0.0096	0.0224	0.0115	0.0112	0.1054
7-8-2-2	ND	0.0124	ND	0.0185	0.0058	ND	0.0383	0.0395	ND	ND	0.0240
7-8-2-4	2.7326	1.2243	6.0385	2.3468	0.5510	3.2359	4.5306	0.7826	ND	2.0567	ND
8-8-0-2	0.3195	ND	1.8908	0.9163	0.0361	0.0999	0.5356	0.1029	0.1368	ND	0.0029
8-9-1-4	ND	ND	ND	0.0112	ND	ND	ND	0.0080	ND	ND	0.0037
10-10-1-1	ND	ND	ND	0.0032	ND	ND	ND	0.0007	ND	ND	ND
4-13-0-0	0.1922	0.3180	0.5973	0.0857	0.1992	0.2241	0.3752	0.0372	ND	0.4934	ND
4-16-0-0	0.0573	ND	0.0338	0.0295	ND	ND	0.0192	0.0379	0.0569	0.0414	ND
4-17-0-0	ND	ND	ND	0.1828	ND	ND	0.6534	0.2592	ND	ND	1.0435
5-7-2-1	0.3486	0.1831	0.5455	0.4414	0.0664	0.2042	0.3433	0.3667	ND	ND	0.8199
5-8-3-1	ND	ND	ND	0.0430	ND	ND	0.0131	0.0432	ND	ND	0.0705
6-3-2-0	0.2139	2.6856	0.2594	0.5442	0.8670	0.8883	1.9262	2.0760	0.6539	0.7730	0.8007
6-5-1-1	0.7171	0.6445	0.7052	0.4299	0.4628	0.4964	0.9292	0.8026	0.1117	0.5359	ND
6-6-0-1	0.0396	ND	0.2801	0.1813	ND	ND	0.0520	0.0129	0.1756	ND	0.0325
6-6-1-0	0.7463	ND	0.5855	0.7484	ND	0.7678	0.5482	0.4669	0.2440	0.7520	0.7960
6-6-1-1	1.1002	0.9416	0.8566	0.8307	0.3881	0.7267	1.0929	0.6474	1.2682	2.1920	1.7005
7-4-1-0	ND	0.4968	0.0127	0.0094	0.1163	0.0302	0.0744	0.0252	0.0942	0.1060	0.0116
7-4-1-1	ND	0.1066	ND	0.0148	0.0730	ND	0.0534	0.0551	ND	ND	0.0149
7-5-0-0	2.1337	ND	7.6868	2.0242	ND	ND	4.7008	0.6340	6.7546	0.4969	0.0101
7-5-1-0	ND	0.0524	0.0447	0.0930	0.0173	0.1466	0.1007	0.0470	ND	0.0984	0.0479
7-6-1-1	0.0612	0.1234	0.0835	0.0311	0.1006	0.0748	0.1345	0.0831	0.1575	0.0459	0.1256
7-6-1-2	6.9347	1.8745	2.4661	0.3086	2.2789	1.5215	0.8091	0.1866	1.5409	ND	ND
7-6-1-3	ND	0.1743	ND	0.1365	0.0535	0.0743	0.1612	0.1380	0.7277	0.3820	0.1393
7-7-0-1	0.7484	ND	2.5590	1.2309	ND	0.0138	0.6863	0.1234	ND	0.1220	ND
7-7-1-0	0.1862	0.6364	0.2334	0.1164	0.2575	0.1014	0.2097	0.1011	0.3750	ND	0.2971
7-7-1-1	0.1411	0.6671	0.3452	0.1720	0.4401	0.2203	0.2423	0.1784	ND	0.3365	0.0182
7-7-1-2	0.0414	0.2496	0.1431	0.2176	0.1290	0.0492	0.1524	0.1797	ND	ND	0.1948
7-7-1-3	0.0071	0.0583	0.0496	0.1266	0.0173	ND	0.0535	0.0872	0.0723	0.0988	0.1599
7-8-1-1	0.5720	1.5039	1.8682	2.5233	1.1583	ND	1.6114	1.7344	ND	ND	3.8279
7-8-1-2	0.0189	0.0043	0.0205	0.0201	0.0053	ND	0.0168	0.0252	ND	0.0492	0.0199
8-6-1-0	ND	ND	ND	ND	ND	ND	ND	0.1478	ND	ND	ND
8-6-1-1	ND	0.0240	ND	0.0053	ND	ND	0.0093	0.0090	ND	ND	0.0357
8-8-0-0	0.1580	ND	0.2756	ND	ND	ND	ND	0.0891	ND	ND	ND
8-8-1-2	0.5504	0.0156	ND	ND	ND	0.8879	ND	0.1735	ND	ND	0.0896
8-9-0-2	0.0934	ND	0.1511	ND	ND	0.2732	ND	0.1199	ND	0.3249	0.0997

Supplementary Table S2. Relative standard deviation (%RSD) of the relative abundance of the identified *N*-glycans. A four-digit *N*-glycan nomenclature was used in the following order: *N*-acetylglucosamine, Hexose, Fucose, *N*-acetylneuraminic acid (GlcNAc,Hex,Fuc,NeuAc).

Glycan	H12 Alpha	H15 Beta	H14 Gamma	H23 Delta	H17 Epsilon	H29 Eta	H28 Iota	H1B Kappa	H32 Lambda	H38 Mu	H41 Omicron
2-8-0-0	ND	ND	ND	0.0057	ND	ND	0.0010	0.0009	ND	ND	0.0008
3-4-0-1	0.0052	0.0797	0.0101	0.0041	0.1296	0.1688	0.0077	0.3167	0.1305	0.0327	0.0166
3-4-1-1	0.0047	ND	0.0075	0.0045	ND	ND	0.0010	0.3998	0.0283	0.4488	ND
3-5-0-0	0.0003	0.0086	ND	0.0038	ND	ND	ND	0.0056	0.0001	0.0001	0.0013
3-5-0-1	0.0203	ND	0.0721	0.0042	0.1921	0.2988	0.0405	0.0604	0.3004	0.0183	0.0097
3-5-1-0	0.0045	0.0769	0.0038	0.0120	0.0486	0.0566	0.0019	0.0033	0.0612	0.1648	0.0114
3-5-1-1	0.0132	0.0977	0.0186	0.0024	0.1029	0.0370	0.0113	0.0112	0.1048	0.0293	0.0265
3-6-0-1	ND	0.0017	ND	0.0033	ND	ND	0.0039	0.0021	0.0382	ND	0.0047
3-6-1-0	0.0346	0.9744	ND	0.0128	0.0489	0.0524	0.0054	0.0214	0.1500	0.0283	0.0030
3-6-1-1	ND	0.0371	0.0153	0.0183	0.0779	1.0497	0.0068	0.0140	0.1015	0.2331	ND
3-7-1-0	0.0016	ND	ND	0.0022	ND	ND	ND	0.0015	0.0041	ND	0.0003
3-8-0-0	0.0239	0.0182	0.0375	0.0039	0.0038	0.0463	0.0201	0.0183	0.0404	0.0493	ND
4-4-0-1	0.1158	0.2404	0.0044	0.0513	0.2670	0.0292	0.0104	0.0207	0.2101	0.0085	0.0157
4-4-1-0	0.0132	0.2944	0.0080	0.0023	0.3761	0.3708	0.0400	0.0005	0.6028	0.9789	0.0194
4-4-1-1	0.1000	0.3365	0.0452	0.0078	1.0571	0.1772	0.0277	0.1222	0.0900	0.0318	0.1927
4-4-2-1	0.0037	0.2602	0.3700	0.0997	ND	0.2209	0.3041	0.3064	0.0347	0.0247	0.1065
4-5-0-0	0.0099	ND	0.0028	0.0048	ND	ND	0.0147	0.0067	ND	ND	0.0080
4-5-0-1	ND	ND	ND	0.0081	ND	1.9997	0.0247	ND	ND	ND	0.0537
4-5-0-2	ND	0.0194	ND	0.0227	0.1504	0.0325	ND	ND	ND	0.0058	0.0273
4-5-1-0	0.1667	0.2055	0.0360	0.0325	0.2528	0.3552	0.0230	0.0086	0.2935	0.1879	0.1944
4-5-1-1	0.0555	0.1398	0.0568	0.0323	1.5633	0.2774	0.0454	0.0210	0.5782	0.1659	0.2659
4-5-1-2	0.0365	0.2413	0.0029	0.0309	0.6505	0.1536	0.0506	0.0392	0.1614	0.0376	0.2260
4-5-2-0	0.0571	0.2388	0.0127	0.0301	0.1723	0.1498	0.0161	0.1123	0.0391	1.0675	0.2952
4-5-2-1	0.0246	0.0272	0.0246	0.0109	0.0670	0.1795	0.0208	0.0363	0.1164	0.0772	0.0321
4-5-3-0	0.0137	ND	0.2157	0.0920	0.0708	8.5662	0.0438	0.3584	0.4426	0.0205	ND
4-6-0-1	ND	ND	0.0077	0.0218	ND	0.0484	0.0081	0.0043	0.1297	ND	0.0045
4-6-1-0	0.0062	ND	0.0207	0.0058	ND	ND	0.0172	0.0072	0.0086	0.0240	ND
4-6-1-1	0.0385	0.0051	0.0004	ND	ND	0.0061	ND	0.0009	0.0069	0.0020	0.0034
4-6-1-2	0.0047	ND	0.0059	0.0055	ND	0.0242	0.0042	0.0008	ND	ND	0.0022
4-6-2-0	0.1205	0.1431	0.0397	0.0650	0.2957	0.1480	0.0127	0.0296	ND	0.0298	0.1332
4-6-2-1	0.0068	0.1201	0.0164	0.0378	0.0472	0.0291	0.0135	0.0235	0.0242	0.0033	0.0369
4-7-1-0	0.0067	ND	0.0065	0.0019	ND	ND	0.0026	0.0006	ND	0.0254	ND
5-3-1-0	0.0125	0.2943	0.0213	0.0033	2.9328	0.7148	0.0492	0.0009	0.0385	0.0453	0.0206
5-4-0-0	0.0028	ND	0.0062	0.0041	0.0142	ND	0.0020	0.0038	0.0122	ND	0.0052
5-4-1-0	0.0764	0.3024	0.0128	0.0122	0.8299	0.3866	0.0216	0.0437	0.1838	ND	0.1057
5-4-1-1	0.0401	0.1649	0.0074	0.0484	0.4636	0.9747	0.0632	0.0621	0.1042	0.0750	0.0415
5-4-2-0	0.0899	0.3468	0.2023	0.0619	2.3779	0.4047	0.0490	0.1389	0.2022	0.0308	0.0508
5-4-2-1	0.0349	0.1510	0.1908	0.0422	0.4846	0.3505	0.0217	0.0588	0.0289	0.0730	0.0688
5-5-0-0	ND	0.0218	0.0007	0.0023	ND	ND	0.0019	0.0153	0.0056	ND	0.0102
5-5-0-1	0.0295	0.1730	0.0560	0.0146	ND	ND	0.0848	ND	ND	0.0016	ND
5-5-1-0	0.0616	0.4931	0.0591	0.0183	0.9288	0.4413	0.0469	0.0519	0.3408	0.1192	0.0886
5-5-1-1	0.0175	0.1950	0.0298	0.0208	0.5236	0.2934	0.0338	0.0364	0.0970	0.0954	0.0327
5-5-2-0	0.0571	0.0741	0.0893	0.0335	0.1240	0.1552	0.0504	0.0454	0.0359	0.0512	0.0597
5-5-2-1	0.0556	0.0471	0.1119	0.0189	0.1746	19.7351	0.0341	0.0296	0.0242	0.0338	0.0585
5-5-3-0	0.0025	0.0008	0.0030	0.0111	0.0227	ND	0.0027	0.0182	ND	0.0071	0.0583
5-6-1-0	0.0667	0.1091	0.0046	0.0224	0.6012	0.0561	0.0052	0.0110	0.0366	0.0069	0.0134
5-6-1-1	0.1331	0.1891	0.0487	0.0190	0.2893	ND	0.0464	0.0587	ND	ND	0.1017
5-6-1-2	0.0316	0.0458	0.0367	0.0487	0.2822	0.1616	0.0072	0.0317	0.1358	0.0223	0.1445
5-6-1-3	0.0285	0.0736	0.0239	0.0315	0.2419	0.5905	0.0715	0.0259	0.6601	0.0096	0.0948
5-6-2-1	0.0323	0.1184	0.0086	0.0233	0.0275	0.0604	0.0105	0.0288	0.0263	0.0069	0.0298
5-6-2-2	0.0124	ND	ND	0.0725	ND	ND	0.0425	0.0829	ND	0.0244	0.0892
5-7-2-0	0.0175	0.0653	0.0081	0.0272	0.0329	0.0164	0.0117	0.0106	0.1744	0.0093	0.0252
6-3-1-0	0.0015	0.5163	0.0006	0.0021	1.4886	0.1018	0.0267	0.0030	0.0426	0.0220	0.0091
6-4-1-0	0.0042	0.4480	0.0033	0.0029	0.1751	0.0949	0.0101	0.0012	0.0554	0.0037	0.0143
6-4-1-1	0.0073	0.2262	0.0045	0.0061	0.1054	0.0719	0.0024	0.0155	ND	0.0047	0.0121
6-4-2-0	0.0025	0.0277	ND	0.0039	0.0185	0.0113	0.0030	0.0056	0.2559	0.0010	0.0034
6-5-1-0	0.0095	0.0283	0.0032	0.0074	0.0808	0.1165	0.0178	0.0049	0.0558	0.0774	0.0080
6-5-1-2	0.0237	0.0280	ND	0.0038	0.0166	0.0091	0.0065	0.0052	0.0129	0.0024	0.0164
6-5-2-1	0.1320	0.1992	0.2156	0.0371	0.1071	0.8790	0.1171	0.0446	ND	ND	1.8678

Supplementary Table S2. Continued…

Glycan	H12 Alpha	H15 Beta	H14 Gamma	H23 Delta	H17 Epsilon	H29 Eta	H28 Iota	H1B Kappa	H32 Lambda	H38 Mu	H41 Omicron
6-5-3-0	0.0131	0.0809	0.0158	0.0473	0.0222	ND	0.0172	0.0242	0.0356	0.0389	0.0924
6-5-4-0	ND	0.0081	ND	ND	ND	0.0535	ND	0.0033	0.0080	ND	0.0133
6-6-1-2	0.0270	0.1279	0.0286	0.0064	0.0425	0.0471	0.0306	0.0119	0.0270	0.0337	0.0555
6-6-1-3	0.0141	ND	0.0056	0.0037	0.0132	0.0144	0.0187	0.0087	ND	0.0185	0.0652
6-7-1-0	0.0672	0.5561	0.0081	0.0043	0.7849	0.0961	0.0074	0.0045	ND	0.0120	0.1242
6-7-1-1	0.0665	0.3514	0.0216	0.0049	0.4707	0.2830	0.0158	0.0371	0.1187	0.0505	0.0230
6-7-1-2	0.0574	0.3376	0.0379	0.0388	0.4891	0.1537	0.0941	0.0347	4.5226	0.0244	0.0196
6-7-1-3	0.0486	0.0844	0.0041	0.0138	0.1443	0.1798	0.1539	0.0350	0.0995	0.0200	0.1321
6-7-1-4	0.0085	0.6744	0.0298	0.0150	0.1428	0.0192	0.2021	0.0412	0.0700	0.0545	0.1152
6-7-2-2	0.0104	0.0851	0.0099	0.0093	0.0282	0.0322	0.0169	0.0164	2.4807	0.0216	0.0668
6-7-2-3	0.0039	0.0026	0.0053	0.0115	0.0053	0.0065	0.0244	0.0127	0.0152	0.0043	0.0522
6-8-1-1	0.2404	ND	0.0319	0.0300	ND	ND	0.0092	0.0098	0.8062	ND	ND
7-7-2-1	0.0503	0.0102	0.0298	0.0024	0.0129	ND	0.0071	0.0158	0.0099	0.0016	0.0062
7-8-1-3	0.0016	0.0011	ND	0.0015	ND	ND	0.0094	0.0029	0.0018	ND	0.0004
7-8-1-4	ND	0.0013	ND	0.0004	ND	ND	0.0036	0.0010	0.0008	0.0005	0.0117
7-8-2-2	ND	0.0032	ND	0.0021	0.0010	ND	0.0020	0.0029	ND	ND	0.0026
7-8-2-4	0.0322	0.3391	0.1118	0.0527	0.1528	0.4297	0.0408	0.0206	ND	0.0296	ND
8-8-0-2	0.0253	ND	0.0490	0.0111	0.0295	0.0135	0.0681	0.0049	0.0110	ND	0.0005
8-9-1-4	ND	ND	ND	0.0003	ND	ND	ND	0.0028	ND	ND	0.0013
10-10-1-1	ND	ND	ND	0.0003	ND	ND	ND	0.0002	ND	ND	ND
4-13-0-0	0.0119	0.1075	0.0133	0.0054	0.0842	0.0213	0.0200	0.0022	ND	0.0073	ND
4-16-0-0	0.0066	ND	0.0058	0.0006	ND	ND	0.0061	0.0034	0.0116	0.0125	ND
4-17-0-0	ND	ND	ND	0.1718	ND	ND	0.3777	0.0265	ND	ND	0.1514
5-7-2-1	0.0120	0.0071	0.0116	0.0454	0.0328	0.0217	0.0085	0.0107	ND	ND	0.0125
5-8-3-1	ND	ND	ND	0.0046	ND	ND	0.0009	0.0006	ND	ND	0.0070
6-3-2-0	0.0204	0.9226	0.0047	0.0164	0.2728	0.1833	0.0573	0.0354	0.0516	0.0168	0.0321
6-5-1-1	0.0245	0.0604	0.0242	0.0090	0.0332	0.0588	0.0337	0.0266	0.0087	0.0232	ND
6-6-0-1	0.0034	ND	0.0068	0.0075	ND	ND	0.0008	0.0002	0.0082	ND	0.0027
6-6-1-0	0.0196	ND	0.0122	0.0044	ND	0.1015	0.0080	0.0038	0.0234	0.0147	0.0229
6-6-1-1	0.0391	0.0319	0.0197	0.0219	0.1055	0.1033	0.0213	0.0214	0.1016	0.0323	0.0769
7-4-1-0	ND	0.5382	0.0003	0.0004	0.0383	0.0042	0.0022	0.0004	0.0076	0.0019	0.0009
7-4-1-1	ND	0.0015	ND	0.0020	0.0131	ND	0.0010	0.0009	ND	ND	0.0024
7-5-0-0	0.0287	ND	0.1730	0.0596	ND	ND	0.1276	0.0220	0.6309	0.0039	0.0023
7-5-1-0	ND	0.0248	0.0045	0.0046	0.0014	0.0193	0.0006	0.0017	ND	0.0017	0.0024
7-6-1-1	0.0040	0.0441	0.0012	0.0011	0.0141	0.0104	0.0034	0.0024	0.0140	0.0012	0.0108
7-6-1-2	0.2754	0.3000	0.0348	0.0163	0.2091	0.1971	0.0201	0.0049	0.1291	ND	ND
7-6-1-3	ND	0.0328	ND	0.0063	0.0079	0.0101	0.0324	0.0150	0.0533	0.0140	0.0320
7-7-0-1	0.0661	ND	0.0232	0.0323	ND	0.0021	0.0128	0.0049	ND	0.0016	ND
7-7-1-0	0.0212	0.2248	0.0113	0.0209	0.1270	0.0132	0.0121	0.0017	0.0291	ND	0.0498
7-7-1-1	0.0137	0.1284	0.0140	0.0182	0.0194	0.0286	0.0130	0.0016	ND	0.0134	0.0008
7-7-1-2	0.0046	0.0708	0.0046	0.0041	0.0535	0.0092	0.0201	0.0105	ND	ND	0.0139
7-7-1-3	0.0006	0.0271	0.0016	0.0019	0.0060	ND	0.0120	0.0050	0.0052	0.0020	0.0126
7-8-1-1	0.0485	0.3781	0.0224	0.0720	0.3016	ND	0.0151	0.0168	ND	ND	0.1154
7-8-1-2	0.0020	0.0012	0.0016	0.0004	0.0028	ND	0.0056	0.0012	ND	0.0013	0.0018
8-6-1-0	ND	ND	ND	ND	ND	ND	ND	0.0515	ND	ND	ND
8-6-1-1	ND	0.0199	ND	0.0009	ND	ND	0.0012	0.0002	ND	ND	0.0021
8-8-0-0	0.0094	ND	0.0136	ND	ND	ND	ND	0.0016	ND	ND	ND
8-8-1-2	0.0495	0.0070	ND	ND	ND	0.1133	ND	0.0133	ND	ND	0.0285
8-9-0-2	0.0094	ND	0.0190	ND	ND	0.0342	ND	0.0053	ND	0.0128	0.0302

Supplementary Table S3. Relative abundance of the identified isomeric *N*-glycans. A four-digit *N*-glycan nomenclature was used in the following order: *N*-acetylglucosamine, Hexose, Fucose, *N*-acetylneuraminic acid (GlcNAc,Hex,Fuc,NeuAc), and I = isomer.

Glycan	H12 Alpha	H15 Beta	H14 Gamma	H23 Delta	H17 Epsilon	H29 Eta	H28 Iota	H1B Kappa	H32 Lambda	H38 Mu	H41 Omicron
2-8-0-0, I-1	ND	ND	ND	ND	ND	ND	ND	0.0211	ND	ND	0.0015
2-8-0-0, I-2	ND	ND	ND	ND	ND	ND	ND	0.0101	ND	ND	0.0512
2-8-0-0, I-3	ND	ND	ND	0.0617	ND	ND	0.0063	0.0080	ND	ND	ND
3-4-0-1, I-1	ND	ND	ND	ND	ND	ND	ND	ND	ND	1.1054	ND
3-4-0-1, I-2	0.1979	0.2824	0.4202	0.3243	0.6072	0.3703	0.2617	0.3195	ND	1.0421	0.1064
3-4-0-1, I-3	0.0320	0.1964	0.1877	0.1598	0.5510	0.5475	0.1549	0.0783	1.0122	ND	ND
3-4-0-1, I-4	ND	ND	ND	ND	ND	0.3838	ND	ND	0.6667	ND	ND
3-4-1-1	0.0477	ND	0.2616	0.2623	ND	ND	0.1221	0.4624	0.4736	0.6042	ND
3-5-0-0, I-1	ND	0.0191	ND	ND	ND	ND	ND	ND	ND	0.0061	ND
3-5-0-0, I-2	0.0015	0.0215	ND	0.0253	ND	ND	0.0015	0.0061	0.0008	ND	0.0248
3-5-0-0, I-3	ND	ND	ND	0.0110	ND	ND	0.0002	ND	ND	ND	0.0050
3-5-0-1, I-1	ND	ND	ND	ND	0.8208	ND	ND	ND	0.7763	ND	ND
3-5-0-1, I-2	ND	ND	ND	ND	ND	ND	ND	ND	ND	ND	0.0342
3-5-0-1, I-3	ND	ND	ND	ND	0.2199	ND	ND	ND	0.2834	ND	0.0465
3-5-0-1, I-4	0.3570	ND	0.8169	0.1685	0.0937	0.2979	0.5263	0.0750	ND	ND	0.1633
3-5-0-1, I-5	1.3702	ND	ND	0.2453	ND	ND	0.3117	0.0951	1.1360	1.7300	ND
3-5-0-1, I-6	0.1617	ND	ND	0.1563	ND	ND	0.0451	0.4280	ND	ND	ND
3-5-0-1, I-7	0.6642	ND	0.7484	0.3578	ND	0.4470	0.2243	ND	1.4023	ND	ND
3-5-0-1, I-8	ND	ND	0.3905	0.5622	ND	ND	0.2317	ND	0.5079	ND	ND
3-5-1-0, I-1	ND	ND	ND	ND	ND	ND	ND	ND	ND	0.3935	ND
3-5-1-0, I-2	0.0869	0.1793	0.1428	0.1082	0.2110	ND	0.0832	0.1930	0.3439	0.1346	0.0486
3-5-1-0, I-3	0.0070	0.0771	0.0506	0.0487	0.2034	0.1576	0.0267	0.0512	0.1784	ND	0.0475
3-5-1-0, I-4	ND	ND	ND	ND	ND	0.1020	ND	ND	ND	ND	ND
3-5-1-1, I-1	ND	ND	ND	ND	ND	ND	ND	ND	ND	ND	0.0501
3-5-1-1, I-2	0.1292	0.3722	0.3869	0.4248	0.1621	0.1446	0.3750	0.7316	0.5401	0.4626	0.2229
3-5-1-1, I-3	0.2373	0.3882	0.4745	0.3251	0.4802	ND	0.2591	0.6682	0.7720	0.4665	ND
3-6-0-1, I-1	ND	0.0072	ND	0.0667	ND	ND	0.0323	0.0866	ND	ND	0.0594
3-6-0-1, I-2	ND	ND	ND	ND	ND	ND	ND	ND	0.1285	ND	ND
3-6-0-1, I-3	ND	ND	ND	ND	ND	ND	ND	ND	0.3081	ND	ND
3-6-1-0, I-1	ND	0.3689	ND	ND	ND	ND	ND	ND	0.3894	ND	ND
3-6-1-0, I-2	ND	2.2033	ND	ND	ND	ND	ND	ND	ND	ND	ND
3-6-1-0, I-3	0.5566	0.2328	ND	ND	ND	ND	ND	0.1996	ND	ND	0.0595
3-6-1-0, I-4	0.0691	ND	ND	ND	ND	ND	ND	0.1555	ND	1.7463	0.0846
3-6-1-0, I-5	0.1217	0.4598	ND	0.3683	0.7213	0.3827	0.2680	0.4365	1.4687	ND	ND
3-6-1-1, I-1	ND	0.0120	0.4121	0.1501	0.0342	ND	0.0860	0.3582	0.5078	0.4518	ND
3-6-1-1, I-2	ND	0.2079	0.4753	0.2043	0.1413	0.7962	0.1242	0.3705	0.5184	0.1862	ND
3-7-1-0, I-1	ND	ND	ND	0.0130	ND	ND	ND	0.0031	ND	ND	0.0086
3-7-1-0, I-2	0.0077	ND	ND	0.0200	ND	ND	ND	0.0068	0.0161	ND	ND
3-7-1-0, I-3	ND	ND	ND	ND	ND	ND	ND	ND	0.0248	ND	ND
3-8-0-0	0.1991	0.0635	0.2678	0.3190	0.0460	0.2828	0.2729	0.2993	0.4099	0.9186	ND
4-4-0-1, I-1	0.3890	ND	ND	0.2912	ND	ND	0.9158	0.0445	2.5116	ND	2.0946
4-4-0-1, I-2	5.7141	2.3768	1.2587	1.7082	4.0557	ND	1.4984	1.4086	ND	0.4972	0.2060
4-4-0-1, I-3	0.4066	ND	ND	0.2580	ND	ND	0.4163	ND	ND	ND	ND
4-4-0-1, I-4	ND	ND	0.0662	ND	ND	ND	ND	ND	ND	ND	ND
4-4-0-1, I-5	0.0411	ND	0.1017	0.0177	ND	ND	0.0474	0.0566	ND	ND	0.0513
4-4-0-1, I-6	0.0232	ND	0.2156	0.0751	ND	0.1719	0.1168	0.3864	ND	ND	ND
4-4-1-0, I-1	ND	ND	ND	0.0440	ND	ND	0.0405	0.0009	ND	ND	0.0129
4-4-1-0, I-2	ND	ND	ND	0.0090	ND	ND	0.0328	0.1076	ND	ND	ND
4-4-1-0, I-3	0.9470	ND	ND	ND	ND	ND	ND	0.0323	0.5651	2.0833	0.8794
4-4-1-0, I-4	0.0081	3.5341	0.4229	0.4658	6.7162	2.6718	1.4073	0.0546	ND	ND	0.0268
4-4-1-0, I-5	ND	ND	ND	ND	0.1827	ND	ND	ND	1.1741	ND	0.0089
4-4-1-0, I-6	ND	0.1266	ND	ND	0.2589	ND	ND	ND	ND	0.3428	ND
4-4-1-0, I-7	ND	ND	ND	ND	ND	ND	ND	ND	ND	1.3329	ND
4-4-1-0, I-8	ND	ND	ND	ND	ND	ND	ND	ND	ND	0.4113	ND
4-4-1-1, I-1	ND	ND	ND	ND	ND	ND	ND	ND	0.2117	ND	ND
4-4-1-1, I-2	ND	ND	ND	ND	ND	ND	ND	ND	0.0915	ND	0.4307
4-4-1-1, I-3	0.1682	0.4107	0.3865	0.0777	1.6795	ND	0.2021	0.7824	0.7784	1.6171	2.9188
4-4-1-1, I-4	2.7153	2.1082	1.2163	0.3936	0.8810	1.1569	1.0262	3.0608	ND	0.8259	ND
4-4-2-1, I-1	ND	ND	ND	ND	ND	ND	ND	ND	ND	0.0915	ND

Supplementary Table S3. Continued…

Glycan	H12 Alpha	H15 Beta	H14 Gamma	H23 Delta	H17 Epsilon	H29 Eta	H28 Iota	H1B Kappa	H32 Lambda	H38 Mu	H41 Omicron
4-4-2-1, I-2	0.0290	0.2848	ND	0.2494	ND	ND	0.2315	0.0617	0.2154	ND	0.5218
4-4-2-1, I-3	0.0222	0.2958	ND	0.7501	ND	0.2323	0.3031	0.0883	ND	ND	ND
4-4-2-1, I-4	ND	ND	ND	0.1200	ND	0.3298	ND	ND	ND	ND	0.3615
4-4-2-1, I-5	0.0249	ND	ND	0.3314	ND	ND	ND	0.0864	0.2511	0.2237	0.4533
4-4-2-1, I-6	0.1366	0.1657	0.1560	0.6832	ND	ND	0.2218	0.1547	ND	ND	0.2686
4-4-2-1, I-7	0.7103	0.0358	0.5966	1.7362	ND	ND	0.5730	1.9275	ND	ND	ND
4-4-2-1, I-8	0.0654	0.2170	0.3013	1.2989	ND	0.7033	0.5438	0.7440	ND	0.6169	ND
4-4-2-1, I-9	0.1206	ND	0.2621	0.7129	ND	ND	0.3334	0.2889	ND	ND	ND
4-5-0-0, I-1	0.1064	ND	ND	0.1600	ND	ND	0.1163	0.0054	ND	ND	0.2585
4-5-0-0, I-2	0.0325	ND	0.1204	ND	ND	ND	ND	0.0753	ND	ND	ND
4-5-0-0, I-3	ND	ND	ND	0.0974	ND	ND	0.2712	ND	ND	ND	0.0339
4-5-0-0, I-4	0.0273	ND	ND	0.0542	ND	ND	0.2051	0.0447	ND	ND	ND
4-5-0-0, I-5	0.0396	ND	0.0882	0.0188	ND	ND	0.0350	0.2483	ND	ND	ND
4-5-0-0, I-6	ND	ND	ND	0.0617	ND	ND	0.0732	ND	ND	ND	ND
4-5-0-1, I-1	ND	ND	ND	ND	ND	ND	ND	ND	ND	ND	0.0793
4-5-0-1, I-2	ND	ND	ND	ND	ND	ND	ND	ND	ND	ND	0.0848
4-5-0-1, I-3	ND	ND	ND	ND	ND	ND	ND	ND	ND	ND	0.0639
4-5-0-1, I-4	ND	ND	ND	ND	ND	ND	ND	ND	ND	ND	0.0360
4-5-0-1, I-5	ND	ND	ND	ND	ND	ND	ND	ND	ND	ND	0.4382
4-5-0-1, I-6	ND	ND	ND	ND	ND	4.5445	ND	ND	ND	ND	0.7428
4-5-0-1, I-7	ND	ND	ND	ND	ND	ND	ND	ND	ND	ND	0.0767
4-5-0-1, I-8	ND	ND	ND	0.1353	ND	ND	0.0734	ND	ND	ND	0.0401
4-5-0-2, I-1	ND	ND	ND	ND	ND	ND	ND	ND	ND	ND	0.1145
4-5-0-2, I-2	ND	0.2477	ND	ND	ND	ND	ND	ND	ND	ND	0.0928
4-5-0-2, I-3	ND	ND	ND	ND	ND	ND	ND	ND	ND	ND	0.0681
4-5-0-2, I-4	ND	ND	ND	ND	ND	ND	ND	ND	ND	ND	0.2228
4-5-0-2, I-5	ND	ND	ND	0.5827	3.6608	0.3710	ND	ND	ND	0.2451	0.3520
4-5-1-0, I-1	0.0608	ND	ND	0.3647	0.0925	ND	0.2420	0.0436	ND	ND	0.0875
4-5-1-0, I-2	5.9230	ND	ND	1.7818	ND	ND	1.5283	1.5729	ND	3.1226	2.2667
4-5-1-0, I-3	0.3776	2.5607	1.3191	0.1855	4.2826	ND	0.2457	0.0653	ND	ND	0.3037
4-5-1-0, I-4	ND	ND	ND	ND	ND	2.4542	ND	ND	2.4571	ND	ND
4-5-1-0, I-5	ND	ND	ND	ND	0.1613	ND	ND	ND	ND	0.5998	0.0546
4-5-1-0, I-6	ND	ND	ND	ND	0.1074	ND	ND	ND	ND	ND	ND
4-5-1-1, I-1	ND	ND	ND	ND	ND	ND	ND	0.1493	ND	ND	3.0228
4-5-1-1, I-2	2.6568	4.0340	2.4291	2.1906	1.9123	ND	2.4947	3.5537	2.6921	4.1878	4.4920
4-5-1-1, I-3	0.4237	5.1342	0.3859	0.4401	5.1709	ND	0.3204	ND	ND	5.3384	0.0258
4-5-1-1, I-4	3.1399	ND	2.6055	2.9864	ND	0.6766	2.7232	3.1190	3.6058	ND	ND
4-5-1-1, I-5	ND	ND	ND	ND	ND	0.9466	ND	ND	ND	ND	ND
4-5-1-1, I-6	ND	ND	ND	0.2365	ND	ND	ND	ND	ND	ND	0.1730
4-5-1-2, I-1	ND	ND	ND	ND	ND	ND	ND	ND	0.2242	0.2003	ND
4-5-1-2, I-2	ND	ND	ND	ND	ND	ND	ND	ND	0.2371	ND	ND
4-5-1-2, I-3	ND	ND	ND	ND	ND	ND	ND	ND	ND	0.3534	ND
4-5-1-2, I-4	ND	ND	ND	ND	ND	ND	ND	ND	ND	0.9255	ND
4-5-1-2, I-5	ND	ND	ND	ND	ND	ND	ND	ND	ND	ND	0.1405
4-5-1-2, I-6	0.0312	ND	0.0918	0.1091	ND	ND	0.2108	0.1311	ND	ND	2.1375
4-5-1-2, I-7	0.3700	2.2260	1.2009	1.4896	0.5189	ND	1.0969	1.6038	1.0161	0.8444	3.7853
4-5-1-2, I-8	0.4227	1.8821	1.3059	1.8974	0.6064	0.7392	0.9809	1.0626	0.7542	0.6716	0.0154
4-5-1-2, I-9	ND	ND	ND	ND	ND	0.6457	ND	ND	ND	ND	0.0920
4-5-1-2, I-10	ND	ND	ND	ND	ND	ND	ND	ND	ND	ND	0.1905
4-5-2-0, I-1	ND	ND	ND	ND	ND	ND	ND	ND	ND	ND	0.0691
4-5-2-0, I-2	ND	ND	ND	ND	ND	ND	ND	ND	ND	ND	0.0823
4-5-2-0, I-3	0.5634	0.4603	0.2310	ND	0.3236	ND	ND	0.9708	ND	1.9495	3.2342
4-5-2-0, I-4	3.8086	0.9297	1.0833	0.2426	0.8648	ND	0.4091	3.4995	ND	ND	ND
4-5-2-0, I-5	0.3348	ND	0.9687	1.7378	ND	ND	0.9953	ND	ND	ND	ND
4-5-2-0, I-6	ND	ND	0.1186	0.7696	ND	1.3475	0.8987	ND	ND	ND	ND
4-5-2-0, I-7	ND	ND	0.2186	0.2915	ND	ND	0.3220	ND	ND	ND	ND
4-5-2-0, I-8	ND	ND	ND	ND	ND	ND	ND	ND	0.5070	ND	ND
4-5-2-1, I-1	0.1163	ND	0.5272	ND	ND	ND	ND	1.7067	ND	ND	0.0162
4-5-2-1, I-2	ND	ND	ND	ND	ND	ND	ND	ND	ND	ND	0.1024
4-5-2-1, I-3	ND	ND	ND	ND	ND	ND	ND	ND	ND	ND	0.1010
4-5-2-1, I-4	ND	ND	ND	ND	ND	ND	ND	ND	ND	1.2208	1.4319
4-5-2-1, I-5	ND	0.1315	ND	ND	0.1013	ND	ND	ND	ND	1.9853	1.9826

Supplementary Table S3. Continued…

Glycan	H12 Alpha	H15 Beta	H14 Gamma	H23 Delta	H17 Epsilon	H29 Eta	H28 Iota	H1B Kappa	H32 Lambda	H38 Mu	H41 Omicron
4-5-2-1, I-6	0.4579	0.3579	1.0268	1.1689	0.4697	0.4715	0.4670	2.0425	0.5114	ND	0.1307
4-5-2-1, I-7	0.7766	0.0421	0.0819	2.1980	ND	0.7888	0.7722	0.0903	0.9767	ND	ND
4-5-2-1, I-8	ND	ND	ND	0.1477	ND	ND	0.0669	ND	ND	ND	ND
4-5-2-1, I-9	ND	ND	ND	ND	ND	ND	ND	ND	ND	ND	0.0584
4-5-2-1, I-10	ND	ND	ND	ND	ND	ND	ND	ND	ND	ND	0.0718
4-5-2-1, I-11	0.0238	ND	ND	ND	ND	ND	ND	0.0290	ND	ND	ND
4-5-2-1, I-12	0.0250	ND	ND	0.0683	ND	ND	ND	0.0460	ND	ND	ND
4-5-3-0, I-1	ND	ND	1.0583	0.3443	ND	7.3049	0.1150	0.6164	2.8884	ND	ND
4-5-3-0, I-2	ND	ND	1.5472	0.7242	ND	1.1321	0.8058	0.6049	3.5969	ND	ND
4-5-3-0, I-3	ND	ND	0.2679	1.0834	0.0453	ND	1.0107	ND	ND	ND	ND
4-5-3-0, I-4	ND	ND	0.2777	0.1097	0.0866	ND	0.1122	ND	ND	ND	ND
4-5-3-0, I-5	1.0300	ND	0.6696	2.0534	ND	ND	0.6422	2.5108	ND	0.9384	ND
4-5-3-0, I-6	ND	ND	0.6555	1.2519	ND	3.4014	0.6218	0.9359	ND	ND	ND
4-5-3-0, I-7	ND	ND	0.4113	0.7014	ND	ND	0.3553	0.3253	ND	ND	ND
4-6-0-1, I-1	ND	ND	ND	ND	ND	ND	ND	ND	0.9033	ND	ND
4-6-0-1, I-2	ND	ND	ND	ND	ND	0.4479	ND	ND	0.5045	ND	ND
4-6-0-1, I-3	ND	ND	0.4110	0.4170	ND	ND	0.0525	0.1134	0.2087	ND	0.0939
4-6-1-0, I-1	ND	ND	0.0770	0.0616	ND	ND	0.3674	0.1396	ND	0.3240	ND
4-6-1-0, I-2	0.0844	ND	0.1115	0.0844	ND	ND	0.0327	0.3042	ND	0.4179	ND
4-6-1-0, I-3	ND	ND	0.0459	0.0525	ND	ND	0.0343	0.0552	0.2447	ND	ND
4-6-1-1, I-1	ND	ND	ND	ND	ND	ND	ND	ND	ND	ND	0.0387
4-6-1-1, I-2	ND	ND	ND	ND	ND	ND	ND	ND	ND	ND	0.0557
4-6-1-1, I-3	0.0432	ND	0.0461	ND	ND	ND	ND	0.0470	0.0657	ND	0.0355
4-6-1-1, I-4	0.0626	0.0090	0.0457	ND	ND	0.0469	ND	0.0233	0.0370	ND	0.0067
4-6-1-1, I-5	0.1243	ND	ND	ND	ND	ND	ND	ND	ND	0.0871	ND
4-6-1-1, I-6	0.1030	ND	ND	ND	ND	ND	ND	ND	ND	ND	ND
4-6-1-1, I-7	0.0390	ND	ND	ND	ND	ND	ND	ND	ND	0.0393	ND
4-6-1-1, I-8	0.0536	ND	ND	ND	ND	ND	ND	ND	ND	ND	ND
4-6-1-1, I-9	0.0673	ND	ND	ND	ND	ND	ND	ND	ND	ND	ND
4-6-1-2, I-1	ND	ND	0.0466	0.0284	ND	ND	0.0378	0.0416	ND	ND	ND
4-6-1-2, I-2	0.0516	ND	0.0520	0.0331	ND	0.1926	0.0202	0.0344	ND	ND	0.0621
4-6-1-2, I-3	ND	ND	ND	ND	ND	ND	ND	ND	ND	ND	0.0111
4-6-2-0, I-1	ND	ND	ND	ND	ND	ND	ND	ND	ND	ND	0.8529
4-6-2-0, I-2	0.7926	1.1264	0.5055	0.4590	0.3104	0.9144	0.5182	1.0558	ND	0.9099	1.0329
4-6-2-0, I-3	0.8387	0.5328	0.4361	0.4487	0.8534	ND	0.3886	0.8254	ND	ND	ND
4-6-2-1, I-1	ND	ND	ND	ND	ND	ND	ND	ND	ND	ND	0.0458
4-6-2-1, I-2	ND	ND	0.0265	ND	ND	ND	0.0629	0.0507	ND	ND	0.6244
4-6-2-1, I-3	0.1407	0.2776	0.3430	0.4274	0.1007	ND	0.2887	0.5836	0.3013	0.1640	0.4890
4-6-2-1, I-4	0.0665	0.0380	0.1277	0.2094	0.0509	0.1623	0.0924	0.1778	0.0941	0.0633	0.0173
4-6-2-1, I-5	ND	ND	ND	ND	ND	0.0586	ND	ND	ND	ND	ND
4-7-1-0	0.1623	ND	0.0781	0.7451	ND	ND	0.0905	0.0328	ND	0.5302	ND
5-3-1-0, I-1	0.0100	0.0699	ND	ND	0.1924	0.2133	0.1276	0.0929	ND	ND	0.0290
5-3-1-0, I-2	ND	0.2536	ND	ND	0.7035	0.2929	0.1420	ND	ND	1.0557	0.0885
5-3-1-0, I-3	0.1181	2.1808	0.2366	0.0607	5.4914	1.4821	0.7209	ND	0.4980	ND	0.3763
5-3-1-0, I-4	0.3644	6.3807	0.8423	0.2980	7.5359	3.3152	2.2504	ND	ND	3.4889	0.4570
5-4-0-0, I-1	ND	ND	ND	ND	ND	ND	ND	0.0172	ND	ND	0.0147
5-4-0-0, I-2	ND	ND	ND	0.0155	ND	ND	ND	ND	ND	ND	0.0471
5-4-0-0, I-3	0.0184	ND	0.1017	0.0394	0.0256	ND	0.0737	0.2653	0.1452	ND	0.1151
5-4-1-0, I-1	0.3467	0.4219	0.2209	0.2061	0.4456	ND	0.7550	0.7482	0.2853	ND	0.4097
5-4-1-0, I-2	ND	0.4651	0.1572	0.1120	1.4146	0.7307	0.3315	ND	0.4138	ND	0.3177
5-4-1-0, I-3	0.2504	1.0082	0.1584	0.0354	0.7712	ND	ND	0.1659	1.5709	ND	0.1564
5-4-1-0, I-4	0.2056	ND	0.9386	0.1647	3.6006	ND	0.4774	1.3062	ND	ND	0.7381
5-4-1-0, I-5	0.9145	3.4561	ND	1.1608	ND	1.9860	1.3772	0.0744	ND	ND	0.1413
5-4-1-1, I-1	ND	ND	ND	ND	ND	ND	ND	ND	ND	ND	0.0935
5-4-1-1, I-2	ND	ND	ND	ND	ND	ND	ND	ND	ND	ND	0.2460
5-4-1-1, I-3	0.0847	1.0937	0.2038	0.1320	0.3326	ND	0.5354	0.6326	ND	ND	0.3185
5-4-1-1, I-4	0.1938	0.7893	0.2475	0.2185	0.0724	0.2520	0.4624	0.6079	0.2946	ND	0.2351
5-4-1-1, I-5	ND	0.1121	0.0785	0.0937	0.1517	ND	0.1670	0.1976	0.3831	3.1603	0.0603
5-4-1-1, I-6	ND	0.1485	0.0247	ND	0.1473	ND	0.1140	ND	ND	ND	ND
5-4-1-1, I-7	0.1110	0.1099	0.0371	0.0452	ND	0.6545	ND	ND	ND	ND	ND
5-4-1-1, I-8	0.1103	ND	0.1296	0.1255	0.4272	ND	0.2873	ND	ND	ND	ND
5-4-1-1, I-9	ND	ND	0.0245	ND	ND	0.3564	0.0673	ND	0.1142	0.3945	0.0741

Supplementary Table S3. Continued…

Glycan	H12 Alpha	H15 Beta	H14 Gamma	H23 Delta	H17 Epsilon	H29 Eta	H28 Iota	H1B Kappa	H32 Lambda	H38 Mu	H41 Omicron
5-4-1-1, I-10	0.0460	0.2727	0.1411	0.1911	0.1692	ND	0.1894	0.2928	ND	ND	1.0157
5-4-1-1, I-11	0.4361	0.2684	0.3212	1.2501	0.5768	0.1563	0.4591	1.8957	0.3086	0.9934	ND
5-4-2-0, I-1	ND	ND	ND	ND	ND	ND	ND	ND	ND	ND	2.0000
5-4-2-0, I-2	1.1240	3.3122	1.3323	1.2564	4.6155	ND	3.9434	5.5582	2.5502	1.8137	ND
5-4-2-0, I-3	ND	ND	ND	ND	ND	2.5679	ND	ND	ND	ND	ND
5-4-2-0, I-4	ND	0.4843	0.2458	ND	0.3408	ND	ND	ND	ND	ND	ND
5-4-2-0, I-5	0.2188	0.2665	0.2005	0.8397	0.2713	0.4215	0.3938	1.1206	ND	0.2390	ND
5-4-2-0, I-6	ND	0.1737	ND	ND	ND	ND	ND	ND	ND	ND	ND
5-4-2-1, I-1	ND	ND	ND	ND	ND	ND	ND	ND	ND	0.4938	0.1241
5-4-2-1, I-2	ND	0.0383	ND	0.1875	0.0501	ND	0.1967	0.4449	ND	ND	1.4655
5-4-2-1, I-3	0.4792	1.1160	1.5624	2.2950	0.9208	0.2342	2.5016	4.5444	ND	2.5556	ND
5-4-2-1, I-4	ND	ND	ND	ND	ND	1.6136	ND	ND	ND	ND	ND
5-4-2-1, I-5	0.2666	ND	0.5676	ND	ND	ND	ND	ND	0.4023	1.3382	ND
5-4-2-1, I-6	0.1974	ND	0.3357	ND	ND	0.7450	ND	ND	ND	ND	ND
5-4-2-1, I-7	0.2267	ND	0.1449	ND	ND	ND	ND	ND	ND	ND	ND
5-4-2-1, I-8	0.2630	ND	ND	ND	ND	ND	ND	ND	ND	ND	ND
5-5-0-0, I-1	ND	ND	ND	ND	ND	ND	ND	ND	ND	ND	0.0293
5-5-0-0, I-2	ND	ND	ND	ND	ND	ND	ND	ND	ND	ND	0.3430
5-5-0-0, I-3	ND	0.1255	0.0639	0.1479	ND	ND	0.0915	0.4951	0.0614	ND	ND
5-5-0-1, I-1	0.2643	ND	0.5206	0.1011	ND	ND	1.0570	ND	ND	0.0521	ND
5-5-0-1, I-2	ND	ND	ND	ND	ND	ND	ND	ND	ND	ND	ND
5-5-0-1, I-3	ND	1.7665	ND	ND	ND	ND	ND	ND	ND	ND	ND
5-5-0-1, I-4	ND	ND	ND	ND	ND	ND	ND	ND	ND	ND	ND
5-5-1-0, I-1	ND	ND	ND	ND	ND	ND	ND	ND	ND	ND	0.3768
5-5-1-0, I-2	ND	ND	ND	ND	ND	ND	ND	ND	ND	ND	0.2445
5-5-1-0, I-3	0.2047	ND	ND	0.3119	0.5860	0.6959	0.9010	3.2010	0.2868	ND	ND
5-5-1-0, I-4	ND	ND	ND	ND	ND	ND	ND	ND	ND	ND	3.3137
5-5-1-0, I-5	3.0507	ND	ND	0.0879	ND	ND	0.1640	ND	4.1668	4.8535	ND
5-5-1-0, I-6	ND	6.1993	2.0553	2.4382	4.3932	2.5575	2.3573	0.1746	ND	ND	0.0772
5-5-1-1, I-1	ND	ND	ND	ND	ND	ND	ND	ND	ND	ND	0.1946
5-5-1-1, I-2	0.1536	0.0443	0.1625	ND	ND	0.1784	ND	0.1303	2.2238	0.5712	0.1370
5-5-1-1, I-3	ND	ND	ND	ND	0.2104	ND	ND	ND	ND	ND	ND
5-5-1-1, I-4	ND	ND	ND	ND	0.1963	0.3567	ND	ND	ND	3.5642	2.2977
5-5-1-1, I-5	1.5429	1.8165	1.6505	2.1293	1.2662	1.5510	1.3615	2.6184	ND	ND	ND
5-5-2-0, I-1	ND	0.0137	0.0599	ND	ND	0.9931	0.0958	0.0525	ND	1.1757	1.4527
5-5-2-0, I-2	0.8937	1.1976	ND	1.8570	0.3439	ND	0.7172	2.2101	ND	ND	0.4563
5-5-2-0, I-3	0.3409	0.2884	0.6766	0.4167	ND	ND	0.2697	0.2910	ND	ND	ND
5-5-2-0, I-4	ND	0.0095	0.3034	ND	ND	ND	ND	0.0740	0.3792	0.6266	ND
5-5-2-1, I-1	ND	ND	ND	ND	ND	ND	ND	ND	ND	ND	0.0581
5-5-2-1, I-2	0.1173	0.0594	ND	ND	0.1136	0.1906	ND	0.0717	ND	ND	0.0862
5-5-2-1, I-3	ND	ND	ND	ND	ND	11.8703	ND	ND	ND	ND	0.2415
5-5-2-1, I-4	0.2880	0.1845	0.3330	0.2783	0.0822	ND	0.2369	0.2154	ND	ND	0.4403
5-5-2-1, I-5	0.3967	0.1515	0.5113	0.4256	0.3250	0.4774	0.3251	0.2168	ND	0.6294	ND
5-5-2-1, I-6	ND	ND	ND	ND	ND	ND	ND	ND	0.2796	0.6940	0.7213
5-5-2-1, I-7	0.1662	0.0423	0.2474	0.8131	0.0233	ND	0.1663	0.5916	ND	ND	ND
5-5-3-0, I-1	ND	ND	ND	ND	ND	ND	ND	ND	ND	ND	0.2957
5-5-3-0, I-2	0.2760	0.0688	0.2206	1.5202	0.0995	ND	0.2372	0.9523	ND	0.5112	ND
5-6-1-0, I-1	0.1963	ND	0.0610	0.0316	0.1761	ND	0.0948	0.0464	ND	ND	0.1215
5-6-1-0, I-2	0.0389	0.8333	0.0161	ND	ND	ND	ND	0.3837	ND	0.4408	0.4439
5-6-1-0, I-3	ND	ND	0.3523	0.4007	0.7598	0.4982	0.2585	ND	ND	ND	0.0211
5-6-1-0, I-4	ND	ND	0.0311	ND	ND	ND	ND	ND	0.5957	ND	ND
5-6-1-0, I-5	1.4352	ND	ND	ND	0.2267	ND	ND	0.0107	ND	ND	0.0393
5-6-1-0, I-6	0.0932	0.0110	ND	0.0247	0.2555	ND	ND	0.0363	ND	ND	ND
5-6-1-0, I-7	0.0259	ND	0.0146	0.0396	0.5994	ND	0.0183	0.0193	ND	ND	ND
5-6-1-1, I-1	ND	ND	ND	ND	ND	ND	ND	ND	ND	ND	0.1694
5-6-1-1, I-2	0.1427	0.1524	0.1486	0.0815	0.0480	ND	0.1602	0.0941	ND	ND	0.5391
5-6-1-1, I-3	0.4001	0.1578	0.3038	0.0816	0.2267	ND	0.2986	0.1434	ND	ND	1.0043
5-6-1-1, I-4	0.9648	0.6801	0.8007	0.1659	0.2555	ND	0.6174	0.9234	ND	ND	1.7765
5-6-1-1, I-5	1.6602	0.4217	1.2167	0.7720	0.5994	ND	0.8039	0.8604	ND	ND	0.0936
5-6-1-1, I-6	0.1133	ND	0.0728	1.1523	ND	ND	ND	0.0914	ND	ND	ND
5-6-1-1, I-7	0.1479	ND	ND	ND	ND	ND	ND	ND	ND	ND	0.0871
5-6-1-1, I-8	0.1459	ND	ND	0.0974	ND	ND	ND	ND	ND	ND	0.0973

Supplementary Table S3. Continued…

Glycan	H12 Alpha	H15 Beta	H14 Gamma	H23 Delta	H17 Epsilon	H29 Eta	H28 Iota	H1B Kappa	H32 Lambda	H38 Mu	H41 Omicron
5-6-1-2, I-1	ND	ND	ND	ND	ND	0.2233	ND	ND	ND	ND	0.6493
5-6-1-2, I-2	0.1167	0.1610	0.2307	0.1452	0.0403	ND	0.2384	0.1659	ND	ND	1.4369
5-6-1-2, I-3	0.2741	0.4628	0.4024	0.3426	0.3529	0.1853	0.4178	0.2663	0.5256	0.7881	0.5211
5-6-1-2, I-4	0.1084	0.0841	0.2230	0.2525	0.0684	ND	0.1574	0.2099	ND	ND	1.6015
5-6-1-2, I-5	0.1811	0.0723	0.4463	0.5655	0.0262	0.3991	0.3145	0.3676	1.0775	1.7427	0.0977
5-6-1-2, I-6	0.2338	0.1266	0.5228	0.5139	0.3423	ND	0.2494	0.2156	ND	ND	0.0485
5-6-1-2, I-7	ND	ND	ND	ND	0.0531	0.4373	ND	ND	ND	ND	0.5897
5-6-1-3, I-1	ND	ND	ND	ND	ND	0.3607	ND	ND	0.1428	ND	ND
5-6-1-3, I-2	ND	ND	ND	ND	ND	0.7931	ND	ND	0.3154	ND	ND
5-6-1-3, I-3	ND	ND	ND	ND	ND	2.4515	ND	ND	2.1584	ND	ND
5-6-1-3, I-4	ND	ND	ND	ND	ND	ND	ND	ND	0.1761	ND	ND
5-6-1-3, I-5	ND	ND	ND	ND	ND	0.7764	ND	ND	0.7738	ND	ND
5-6-1-3, I-6	0.0543	0.0752	0.1526	0.2214	0.0350	ND	ND	0.1400	ND	0.1161	0.9821
5-6-1-3, I-7	0.0680	0.2620	0.2514	0.3634	0.0862	ND	0.1656	0.1662	0.1236	0.1434	0.3092
5-6-1-3, I-8	0.0695	0.1192	0.3155	0.5516	0.0374	ND	0.2082	0.3352	ND	0.1925	2.0816
5-6-1-3, I-9	0.1163	0.3518	0.4510	0.8798	0.5043	ND	0.2274	0.3055	0.3862	0.2575	0.0480
5-6-1-3, I-10	0.0260	0.0073	ND	0.0858	0.0073	ND	0.2680	0.0443	0.3920	ND	ND
5-6-1-3, I-11	ND	ND	ND	ND	ND	ND	ND	ND	ND	ND	0.2011
5-6-1-3, I-12	0.0112	0.0015	ND	0.0462	ND	ND	ND	0.0186	ND	ND	ND
5-6-2-1, I-1	0.1636	0.0303	ND	0.1768	0.0173	0.0849	0.1169	0.1144	0.4826	ND	0.4541
5-6-2-1, I-2	0.1385	0.0326	0.1341	0.1895	0.0345	ND	0.1352	0.1562	ND	ND	0.3018
5-6-2-1, I-3	0.1459	0.0280	0.1769	0.0787	0.0435	ND	0.0674	0.0439	ND	ND	0.8634
5-6-2-1, I-4	0.0845	0.0204	0.0498	0.3434	0.0257	ND	0.1056	0.2672	ND	1.0383	ND
5-6-2-1, I-5	0.4056	0.0855	0.4774	0.4804	0.0780	0.3547	0.1485	0.3352	ND	ND	ND
5-6-2-2, I-1	ND	ND	ND	0.1356	ND	ND	0.2155	0.2732	ND	ND	ND
5-6-2-2, I-2	ND	ND	ND	1.3754	ND	ND	0.7240	2.3018	ND	1.3069	ND
5-6-2-2, I-3	ND	ND	ND	0.6069	ND	ND	0.7826	0.2614	ND	ND	ND
5-6-2-2, I-4	ND	ND	ND	0.1729	ND	ND	0.1311	ND	ND	ND	ND
5-6-2-2, I-5	ND	ND	ND	0.2492	ND	ND	0.2850	ND	ND	ND	ND
5-6-2-2, I-6	0.0377	ND	ND	ND	ND	ND	ND	ND	ND	ND	0.0455
5-6-2-2, I-7	ND	ND	ND	ND	ND	ND	ND	ND	ND	ND	0.0392
5-6-2-2, I-8	ND	ND	ND	ND	ND	ND	ND	ND	ND	ND	0.2528
5-6-2-2, I-9	ND	ND	ND	ND	ND	ND	ND	ND	ND	ND	0.5518
5-6-2-2, I-10	0.0613	ND	ND	0.2710	ND	ND	ND	ND	ND	ND	0.4778
5-6-2-2, I-11	0.0519	ND	ND	0.4371	ND	ND	ND	ND	ND	ND	0.0163
5-7-2-0, I-1	ND	ND	ND	ND	ND	ND	ND	ND	ND	0.1131	ND
5-7-2-0, I-2	ND	ND	ND	ND	ND	ND	ND	ND	ND	0.0478	ND
5-7-2-0, I-3	ND	ND	ND	ND	ND	ND	ND	ND	ND	ND	0.0277
5-7-2-0, I-4	0.0547	0.0208	ND	0.0787	ND	ND	ND	0.0202	ND	ND	0.0677
5-7-2-0, I-5	0.0790	0.0461	ND	0.0327	0.0126	ND	0.0384	0.0231	ND	ND	0.1626
5-7-2-0, I-6	ND	ND	ND	0.0327	ND	ND	0.0475	ND	ND	ND	ND
5-7-2-0, I-7	0.2194	0.1569	0.1637	0.1658	0.0703	ND	0.1164	0.1873	ND	0.1439	0.1743
5-7-2-0, I-8	0.2643	0.0250	0.1681	0.1641	0.0282	0.1183	0.0972	0.1169	2.3825	0.0425	0.0156
5-7-2-0, I-9	ND	ND	ND	ND	ND	ND	ND	ND	0.1942	ND	0.0135
5-7-2-0, I-10	ND	ND	ND	ND	ND	ND	ND	ND	ND	ND	0.0180
6-3-1-0, I-1	0.0076	ND	0.0098	0.0137	ND	ND	0.1069	0.0956	ND	ND	0.0473
6-3-1-0, I-2	ND	0.0198	ND	ND	0.0551	0.0459	ND	ND	ND	ND	ND
6-3-1-0, I-3	ND	0.0186	ND	ND	0.1028	ND	ND	ND	ND	0.0334	0.0153
6-3-1-0, I-4	0.0346	1.6030	ND	0.0121	2.2270	ND	0.0393	ND	0.2627	0.2265	0.2703
6-3-1-0, I-5	ND	0.0649	0.0078	0.0275	0.0456	0.6463	0.4791	0.0539	ND	0.7357	0.0142
6-3-1-0, I-6	ND	0.2897	ND	ND	ND	ND	0.0569	ND	ND	ND	ND
6-3-1-0, I-7	ND	ND	ND	ND	0.5926	0.2351	0.1583	ND	0.0777	ND	0.0436
6-3-1-0, I-8	ND	0.1217	ND	ND	0.2284	ND	0.1000	ND	0.1039	ND	0.0427
6-4-1-0, I-1	0.0614	0.0403	ND	0.0184	0.7115	0.0347	0.3861	0.0205	0.4611	ND	0.2154
6-4-1-0, I-2	0.0194	0.5818	0.1113	0.0318	0.0290	0.6430	0.0649	0.0916	ND	ND	0.0731
6-4-1-0, I-3	ND	ND	ND	ND	ND	ND	ND	ND	0.0891	0.1516	0.0325
6-4-1-0, I-4	ND	0.0403	ND	ND	0.0775	ND	0.0898	ND	ND	ND	ND
6-4-1-0, I-5	ND	0.5818	ND	ND	0.1303	ND	0.0492	ND	0.1058	0.1648	0.0379
6-4-1-1, I-1	ND	ND	ND	ND	ND	ND	ND	ND	ND	ND	0.0615
6-4-1-1, I-2	0.0177	0.0977	0.0265	0.0104	0.0595	0.0875	0.1131	0.0410	ND	0.0504	ND
6-4-1-1, I-3	ND	ND	ND	ND	ND	ND	ND	ND	ND	ND	0.1282
6-4-1-1, I-4	0.0630	0.5932	0.1570	0.1542	0.2313	0.2096	0.2783	0.3004	ND	0.2423	0.0251

Supplementary Table S3. Continued…

Glycan	H12 Alpha	H15 Beta	H14 Gamma	H23 Delta	H17 Epsilon	H29 Eta	H28 Iota	H1B Kappa	H32 Lambda	H38 Mu	H41 Omicron
6-4-1-1, I-5	ND	ND	ND	0.0180	ND	ND	ND	ND	ND	ND	0.0240
6-4-1-1, I-6	ND	ND	ND	0.0127	ND	ND	ND	ND	ND	ND	0.0114
6-4-2-0, I-1	ND	ND	ND	ND	ND	ND	0.0320	ND	ND	ND	0.0107
6-4-2-0, I-2	ND	ND	ND	ND	0.0575	ND	ND	ND	ND	ND	0.0392
6-4-2-0, I-3	0.0073	0.0611	ND	ND	0.0235	0.0446	0.0293	0.0049	ND	ND	ND
6-4-2-0, I-4	ND	0.0548	ND	0.0479	0.0212	ND	0.0504	0.0661	0.1930	0.0720	ND
6-4-2-0, I-5	0.0096	ND	ND	ND	ND	0.0384	ND	ND	ND	ND	ND
6-5-1-0, I-1	ND	ND	ND	ND	ND	ND	ND	ND	ND	ND	0.0238
6-5-1-0, I-2	ND	ND	ND	ND	ND	ND	ND	ND	ND	ND	0.1034
6-5-1-0, I-3	0.0878	0.4772	0.0875	ND	0.2046	ND	ND	0.0938	0.1110	0.1638	0.0490
6-5-1-0, I-4	0.0404	0.2082	ND	0.0484	0.0788	0.1220	0.1281	ND	0.3421	0.5690	0.0842
6-5-1-0, I-5	0.0432	ND	ND	ND	0.1690	ND	0.2155	ND	ND	ND	0.0092
6-5-1-0, I-6	0.0444	0.0610	ND	ND	0.0471	ND	ND	ND	ND	0.3025	0.0707
6-5-1-0, I-7	0.0267	0.0224	ND	0.0253	ND	ND	0.0895	0.0271	ND	0.1536	0.0722
6-5-1-0, I-8	0.0783	0.0913	0.1150	0.1391	0.0260	ND	0.0357	0.1294	0.2733	0.3052	0.1471
6-5-1-0, I-9	ND	ND	ND	ND	0.1460	0.1310	0.1015	ND	ND	ND	ND
6-5-1-2, I-1	ND	ND	ND	ND	ND	ND	ND	ND	ND	ND	0.0488
6-5-1-2, I-2	ND	0.0362	ND	0.0275	0.0232	ND	0.0752	0.0317	0.0599	ND	0.0587
6-5-1-2, I-3	ND	0.0208	ND	0.0299	0.0286	ND	0.0626	0.0255	ND	ND	0.0737
6-5-1-2, I-4	0.0615	0.0323	ND	0.0737	0.0224	ND	0.0891	0.0697	ND	ND	0.0618
6-5-1-2, I-5	0.0571	0.0367	ND	0.0527	0.0135	ND	0.0512	0.0392	ND	ND	0.0731
6-5-1-2, I-6	0.0592	ND	ND	ND	0.0335	0.0677	ND	0.0364	0.1106	0.1491	0.0998
6-5-1-2, I-7	ND	0.0328	ND	ND	0.0389	ND	ND	ND	ND	ND	ND
6-5-1-2, I-8	ND	0.0436	ND	ND	0.0511	ND	ND	ND	ND	ND	0.0553
6-5-2-1, I-1	ND	0.0690	ND	ND	0.1083	ND	ND	ND	ND	ND	0.0161
6-5-2-1, I-2	ND	ND	0.0982	ND	0.2339	0.2707	0.1661	0.0465	ND	ND	0.2327
6-5-2-1, I-3	ND	0.3458	ND	ND	0.4218	ND	ND	ND	ND	ND	ND
6-5-2-1, I-4	ND	ND	0.1530	0.1433	0.0662	1.3449	0.3066	0.2329	ND	ND	0.0120
6-5-2-1, I-5	0.0999	ND	ND	ND	ND	ND	0.4076	ND	ND	ND	ND
6-5-2-1, I-6	0.2665	ND	ND	ND	ND	ND	ND	ND	ND	ND	ND
6-5-2-1, I-7	0.7789	1.1638	0.7576	0.3822	1.2037	2.6360	1.1642	0.6059	ND	ND	1.3829
6-5-2-1, I-8	0.4871	0.7164	0.7764	0.3478	0.5835	ND	0.5205	0.2392	ND	ND	0.4789
6-5-2-1, I-9	0.2765	0.6153	ND	0.4155	0.1622	ND	0.2402	ND	ND	ND	ND
6-5-2-1, I-10	ND	ND	ND	0.2219	ND	1.5092	ND	0.1677	ND	ND	ND
6-5-2-1, I-11	ND	0.0433	ND	0.2783	0.0752	0.6097	ND	0.0754	ND	ND	ND
6-5-2-1, I-12	ND	0.0227	ND	ND	ND	ND	ND	0.1800	ND	ND	ND
6-5-2-1, I-13	ND	0.0144	ND	ND	0.0402	ND	ND	0.0706	ND	ND	ND
6-5-2-1, I-14	ND	ND	ND	ND	ND	ND	ND	0.0533	ND	ND	ND
6-5-3-0, I-1	ND	ND	ND	ND	ND	ND	ND	ND	ND	1.1475	ND
6-5-3-0, I-2	ND	ND	ND	ND	ND	ND	ND	ND	0.0866	ND	0.1870
6-5-3-0, I-3	ND	ND	0.2841	ND	ND	ND	ND	0.1823	0.1844	0.9330	ND
6-5-3-0, I-4	ND	0.1424	ND	ND	0.1433	ND	ND	ND	0.1991	ND	0.0075
6-5-3-0, I-5	0.2558	ND	ND	ND	ND	ND	ND	ND	ND	ND	0.1341
6-5-3-0, I-6	0.2060	ND	ND	0.2766	ND	ND	ND	0.5535	ND	ND	ND
6-5-3-0, I-7	ND	0.3277	ND	0.2864	0.2857	ND	0.2774	ND	ND	ND	ND
6-5-3-0, I-8	ND	0.0336	ND	ND	0.0294	ND	0.2544	ND	ND	ND	ND
6-5-3-0, I-9	ND	0.0398	ND	ND	0.0434	ND	ND	ND	ND	ND	ND
6-5-3-0, I-10	ND	0.0221	ND	0.1464	0.1019	ND	ND	ND	ND	ND	ND
6-5-4-0, I-1	ND	ND	ND	ND	ND	ND	ND	ND	0.0866	ND	ND
6-5-4-0, I-2	ND	ND	ND	ND	ND	ND	ND	ND	ND	ND	0.0640
6-5-4-0, I-3	ND	ND	ND	ND	ND	0.3511	ND	0.0755	ND	ND	ND
6-5-4-0, I-4	ND	0.0164	ND	ND	ND	ND	ND	0.0819	ND	ND	ND
6-5-4-0, I-5	ND	ND	ND	ND	ND	ND	ND	ND	ND	ND	0.0675
6-6-1-2, I-1	0.0714	ND	ND	ND	ND	ND	0.0695	ND	0.1097	ND	0.1014
6-6-1-2, I-2	0.1266	ND	ND	ND	0.0256	ND	0.1004	0.3745	0.1441	0.2171	0.1118
6-6-1-2, I-3	ND	ND	ND	ND	ND	ND	ND	ND	ND	ND	ND
6-6-1-2, I-4	ND	ND	ND	ND	ND	ND	ND	ND	ND	ND	ND
6-6-1-2, I-5	0.1734	0.1519	0.2848	ND	0.0612	ND	0.1678	ND	ND	0.5599	0.3298
6-6-1-2, I-6	0.1301	ND	ND	0.2499	0.0540	0.1771	0.2477	ND	ND	0.4759	0.1618
6-6-1-2, I-7	0.0523	ND	ND	0.1523	ND	0.2038	0.1327	ND	ND	ND	ND
6-6-1-2, I-8	ND	0.0128	0.0561	0.1283	0.0098	ND	0.0401	0.0642	0.0745	ND	ND
6-6-1-3, I-1	0.1121	ND	0.0636	ND	0.0152	ND	ND	0.3516	ND	ND	0.0338

Supplementary Table S3. Continued…

Glycan	H12 Alpha	H15 Beta	H14 Gamma	H23 Delta	H17 Epsilon	H29 Eta	H28 Iota	H1B Kappa	H32 Lambda	H38 Mu	H41 Omicron
6-6-1-3, I-2	ND	ND	ND	ND	0.0164	ND	ND	ND	ND	ND	0.0745
6-6-1-3, I-3	ND	ND	0.1348	0.2077	0.0085	0.1064	0.1118	ND	ND	0.6201	0.2457
6-6-1-3, I-4	ND	ND	ND	ND	0.0204	ND	ND	ND	ND	ND	ND
6-6-1-3, I-5	ND	ND	0.1882	0.2415	0.0206	ND	0.1035	ND	ND	ND	0.0083
6-7-1-0, I-1	ND	ND	ND	ND	ND	ND	ND	ND	ND	ND	0.5881
6-7-1-0, I-2	1.5964	0.8146	0.5272	0.2439	1.1811	ND	0.5737	0.2724	ND	0.6870	ND
6-7-1-0, I-3	0.3022	ND	ND	ND	ND	0.7097	ND	ND	ND	ND	0.0420
6-7-1-0, I-4	0.1402	0.0107	ND	0.0330	0.0373	ND	0.0293	0.0343	ND	ND	ND
6-7-1-1, I-1	ND	0.0226	ND	ND	0.0254	0.2253	ND	ND	ND	ND	0.2008
6-7-1-1, I-2	0.2035	0.3528	0.0741	ND	0.0562	0.2497	ND	0.0634	ND	ND	0.5302
6-7-1-1, I-3	ND	ND	0.1969	ND	1.0280	ND	ND	ND	0.3030	1.0091	0.3914
6-7-1-1, I-4	0.5426	0.8343	0.4277	0.1111	ND	ND	0.2100	0.1456	0.5423	0.7233	ND
6-7-1-1, I-5	0.4293	0.5121	0.2949	0.2216	0.4655	0.1482	0.4062	0.1609	0.3201	0.3421	0.0690
6-7-1-1, I-6	0.2413	0.0159	0.1006	0.1570	0.0170	0.3954	0.2696	0.0958	0.1551	ND	0.0157
6-7-1-1, I-7	ND	ND	ND	ND	ND	0.2989	ND	ND	ND	ND	ND
6-7-1-1, I-8	0.0826	ND	ND	0.1075	ND	0.1285	0.0812	0.0571	0.1524	ND	0.0259
6-7-1-2, I-1	ND	ND	ND	ND	ND	ND	ND	ND	0.6570	ND	0.6654
6-7-1-2, I-2	0.1663	0.2118	0.5305	ND	0.0466	ND	ND	0.3700	ND	1.4572	1.4357
6-7-1-2, I-3	0.0940	ND	ND	ND	ND	ND	ND	ND	3.6048	ND	ND
6-7-1-2, I-4	0.5540	0.6354	1.0178	0.3987	0.0542	0.2870	0.4819	0.4342	ND	ND	0.0814
6-7-1-2, I-5	0.0784	ND	ND	0.6257	0.7098	0.5880	0.6895	ND	ND	ND	0.0469
6-7-1-2, I-6	0.0942	ND	ND	0.0891	ND	ND	ND	0.0559	ND	ND	0.0149
6-7-1-2, I-7	ND	ND	ND	0.0987	ND	0.1511	ND	0.0322	ND	ND	ND
6-7-1-3, I-1	0.0752	ND	ND	0.4872	ND	ND	ND	0.3438	ND	0.3648	0.1869
6-7-1-3, I-2	0.0469	0.0747	0.4996	0.6577	0.0285	ND	0.4289	ND	ND	ND	0.6781
6-7-1-3, I-3	ND	ND	ND	ND	ND	ND	ND	ND	ND	ND	0.1562
6-7-1-3, I-4	0.1989	ND	0.8798	0.1173	0.0358	0.2276	0.4961	0.3686	0.4604	0.5231	0.0138
6-7-1-3, I-5	ND	ND	ND	ND	ND	0.2068	ND	ND	0.6136	ND	0.0321
6-7-1-3, I-6	0.0323	0.2523	0.0215	0.0680	0.2576	ND	0.0406	0.0418	ND	ND	0.0438
6-7-1-3, I-7	0.0236	0.0092	0.0466	0.0947	0.0085	ND	0.0261	0.0454	ND	ND	0.0298
6-7-1-3, I-8	0.0286	0.0085	0.0396	0.0854	0.0066	ND	0.0252	0.0297	0.0396	ND	0.0054
6-7-1-3, I-9	ND	ND	ND	ND	ND	ND	ND	ND	0.0343	ND	ND
6-7-1-4, I-1	ND	ND	ND	ND	ND	ND	ND	ND	ND	ND	0.4361
6-7-1-4, I-2	0.0261	0.1939	0.2119	0.3924	0.0656	ND	0.2607	0.2226	0.2848	0.1423	ND
6-7-1-4, I-3	ND	ND	ND	ND	ND	ND	ND	ND	ND	ND	1.0811
6-7-1-4, I-4	ND	ND	ND	ND	ND	ND	ND	ND	ND	ND	0.4733
6-7-1-4, I-5	ND	ND	ND	ND	ND	ND	ND	ND	ND	ND	0.1095
6-7-1-4, I-6	0.0707	0.8107	0.5164	0.8194	0.6806	ND	0.4734	0.3121	0.4637	0.3506	0.0145
6-7-1-4, I-7	ND	ND	ND	ND	ND	0.1415	ND	ND	ND	ND	0.1639
6-7-1-4, I-8	0.0134	0.0087	0.0431	0.1350	0.0088	ND	0.0381	0.0618	0.0415	ND	0.0353
6-7-2-2, I-1	ND	ND	ND	ND	ND	ND	ND	ND	ND	ND	0.0356
6-7-2-2, I-2	ND	0.6356	ND	ND	0.0146	ND	ND	ND	ND	ND	0.2813
6-7-2-2, I-3	ND	ND	ND	ND	ND	ND	ND	ND	2.0110	0.9854	0.0342
6-7-2-2, I-4	0.1681	0.0335	0.4126	0.5891	0.0545	ND	0.3014	0.4885	ND	ND	0.0013
6-7-2-2, I-5	ND	ND	ND	ND	ND	ND	ND	ND	ND	ND	0.0288
6-7-2-2, I-6	ND	ND	ND	ND	ND	0.2420	ND	ND	ND	ND	0.0690
6-7-2-2, I-7	ND	ND	ND	ND	ND	ND	ND	ND	ND	ND	0.1553
6-7-2-2, I-8	ND	ND	ND	ND	ND	ND	ND	ND	ND	ND	0.0357
6-7-2-3, I-1	0.0243	0.0152	0.1098	0.2801	0.0109	ND	0.0950	0.1708	ND	0.1102	0.1943
6-7-2-3, I-2	0.0124	0.0327	0.0667	0.1796	0.0194	ND	0.0480	0.0864	ND	0.0527	ND
6-7-2-3, I-3	ND	ND	ND	ND	ND	0.0484	ND	ND	0.1242	ND	ND
6-7-2-3, I-4	ND	ND	ND	ND	ND	ND	ND	ND	0.0698	ND	ND
6-7-2-3, I-5	ND	0.0007	ND	0.0362	ND	ND	ND	0.0209	ND	ND	0.0524
6-8-1-1, I-1	0.4030	ND	0.1635	0.0697	ND	ND	0.4455	0.4175	ND	ND	ND
6-8-1-1, I-2	ND	ND	0.2040	ND	ND	ND	0.1656	ND	ND	ND	ND
6-8-1-1, I-3	8.4384	ND	7.1350	1.2055	ND	ND	3.5171	ND	7.9258	ND	ND
6-8-1-1, I-4	ND	ND	0.1367	1.0895	ND	ND	0.9458	ND	ND	ND	ND
6-8-1-1, I-5	2.4061	ND	1.6177	ND	ND	ND	ND	ND	2.9881	ND	ND
7-7-2-1, I-1	ND	ND	ND	ND	ND	ND	ND	ND	ND	ND	0.0143
7-7-2-1, I-2	0.0405	0.0110	ND	ND	0.0068	ND	ND	0.0198	ND	0.0691	0.0166
7-7-2-1, I-3	0.0422	0.0200	ND	ND	0.0261	ND	ND	0.0250	ND	ND	0.0036
7-7-2-1, I-4	0.1144	ND	0.1551	ND	ND	ND	ND	ND	ND	ND	ND

Supplementary Table S3. Continued…

Glycan	H12 Alpha	H15 Beta	H14 Gamma	H23 Delta	H17 Epsilon	H29 Eta	H28 Iota	H1B Kappa	H32 Lambda	H38 Mu	H41 Omicron
7-7-2-1, I-5	0.0951	ND	ND	ND	ND	ND	ND	ND	ND	ND	ND
7-7-2-1, I-6	ND	ND	0.0743	ND	ND	ND	ND	ND	0.1354	ND	0.0306
7-7-2-1, I-7	0.0998	ND	0.0766	ND	0.0131	ND	ND	ND	ND	ND	0.0109
7-7-2-1, I-8	0.0494	0.0154	0.0721	ND	ND	ND	ND	0.0359	ND	ND	0.0283
7-7-2-1, I-9	ND	ND	ND	0.0431	ND	ND	0.0297	0.0272	ND	ND	0.0346
7-7-2-1, I-10	ND	0.0152	ND	0.0455	0.0109	ND	0.0177	0.0435	ND	ND	ND
7-7-2-1, I-11	0.0288	0.0170	ND	0.0537	0.0089	ND	0.0229	0.0465	ND	ND	0.0030
7-7-2-1, I-12	ND	ND	ND	0.0081	ND	ND	ND	0.0081	ND	ND	0.0122
7-8-1-3	0.0101	0.0070	ND	0.0443	ND	ND	0.0210	0.0441	0.0363	ND	0.0074
7-8-1-4, I-1	ND	0.0024	ND	ND	ND	ND	ND	0.0048	ND	0.0112	0.0173
7-8-1-4, I-2	ND	0.0050	ND	0.0050	ND	ND	0.0018	0.0104	0.0115	ND	0.0405
7-8-1-4, I-3	ND	0.0033	ND	0.0148	ND	ND	0.0054	0.0055	ND	ND	0.0365
7-8-1-4, I-4	ND	ND	ND	0.0091	ND	ND	0.0024	0.0008	ND	ND	0.0040
7-8-1-4, I-5	ND	ND	ND	0.0018	ND	ND	ND	0.0010	ND	ND	0.0071
7-8-2-2, I-1	ND	ND	ND	ND	ND	ND	ND	ND	ND	ND	0.0129
7-8-2-2, I-2	ND	ND	ND	ND	ND	ND	ND	ND	ND	ND	ND
7-8-2-2, I-3	ND	ND	ND	ND	ND	ND	ND	ND	ND	ND	0.0025
7-8-2-2, I-4	ND	ND	ND	ND	ND	ND	ND	ND	ND	ND	0.0062
7-8-2-2, I-5	ND	0.0066	ND	ND	0.0028	ND	ND	0.0243	ND	ND	0.0024
7-8-2-2, I-6	ND	0.0058	ND	0.0185	0.0031	ND	0.0383	0.0152	ND	ND	ND
7-8-2-4, I-1	1.0973	1.2243	1.6236	0.6288	0.5510	ND	1.4823	0.3135	ND	2.0567	ND
7-8-2-4, I-2	0.5535	ND	2.4515	0.4426	ND	1.9773	1.6397	0.1778	ND	ND	ND
7-8-2-4, I-3	0.4181	ND	0.3469	ND	ND	ND	ND	0.1077	ND	ND	ND
7-8-2-4, I-4	0.3360	ND	0.3342	0.5984	ND	0.8251	0.5770	0.0871	ND	ND	ND
7-8-2-4, I-5	ND	ND	0.6989	0.4244	ND	ND	0.5685	ND	ND	ND	ND
7-8-2-4, I-6	0.2020	ND	0.4768	0.1211	ND	0.4335	ND	0.0337	ND	ND	ND
7-8-2-4, I-7	0.0618	ND	ND	0.0478	ND	ND	0.1955	0.0481	ND	ND	ND
7-8-2-4, I-8	0.0641	ND	0.1066	0.0838	ND	ND	0.0677	0.0146	ND	ND	ND
8-8-0-2, I-1	ND	ND	ND	ND	ND	ND	ND	ND	0.1368	ND	ND
8-8-0-2, I-2	0.1373	ND	0.8544	0.3014	ND	0.0058	ND	0.0462	ND	ND	0.0013
8-8-0-2, I-3	0.0873	ND	0.5846	0.1875	ND	ND	0.2541	0.0270	ND	ND	0.0006
8-8-0-2, I-4	0.0429	ND	0.2323	0.2391	ND	ND	0.1573	0.0154	ND	ND	0.0004
8-8-0-2, I-5	0.0343	ND	0.1677	0.1597	0.0361	ND	0.0783	0.0102	ND	ND	0.0004
8-8-0-2, I-6	0.0176	ND	0.0519	0.0286	ND	0.0941	0.0458	0.0041	ND	ND	0.0001
8-9-1-4	ND	ND	ND	0.0112	ND	ND	ND	0.0080	ND	ND	0.0037
10-10-1-1	ND	ND	ND	0.0032	ND	ND	ND	0.0007	ND	ND	ND
4-13-0-0, I-1	ND	ND	0.1546	ND	0.0552	ND	ND	0.0216	ND	0.1000	ND
4-13-0-0, I-2	0.1161	0.2853	0.3528	0.0457	0.1127	ND	0.2990	ND	ND	0.2120	ND
4-13-0-0, I-3	ND	ND	ND	ND	ND	0.2241	ND	ND	ND	0.1154	ND
4-13-0-0, I-4	ND	ND	ND	ND	0.0174	ND	ND	ND	ND	0.0659	ND
4-13-0-0, I-5	0.0760	0.0327	0.0898	0.0400	0.0138	ND	0.0762	0.0156	ND	ND	ND
4-16-0-0, I-1	0.0166	ND	0.0338	ND	ND	ND	ND	ND	ND	ND	ND
4-16-0-0, I-2	ND	ND	ND	ND	ND	ND	ND	ND	0.0569	0.0243	ND
4-16-0-0, I-3	0.0407	ND	ND	0.0295	ND	ND	0.0192	0.0379	ND	0.0171	ND
4-17-0-0, I-1	ND	ND	ND	ND	ND	ND	ND	0.1013	ND	ND	0.2782
4-17-0-0, I-2	ND	ND	ND	0.1828	ND	ND	0.6534	0.1578	ND	ND	0.6027
4-17-0-0, I-3	ND	ND	ND	ND	ND	ND	ND	ND	ND	ND	0.1626
5-7-2-1, I-1	ND	ND	ND	ND	ND	0.2042	ND	ND	ND	ND	ND
5-7-2-1, I-2	0.1023	ND	ND	0.0942	ND	ND	0.0797	ND	ND	ND	0.1397
5-7-2-1, I-3	0.0873	0.0339	0.1171	0.0356	ND	ND	0.0596	0.0599	ND	ND	0.1986
5-7-2-1, I-4	0.0676	0.0453	0.0884	0.0377	ND	ND	0.0503	0.0518	ND	ND	0.1575
5-7-2-1, I-5	ND	0.0221	0.0701	0.0495	ND	ND	0.0310	0.0759	ND	ND	0.3064
5-7-2-1, I-6	ND	0.0293	0.1260	0.0960	ND	ND	0.0532	0.1028	ND	ND	ND
5-7-2-1, I-7	0.0915	0.0525	0.1439	0.1284	0.0664	ND	0.0695	0.0764	ND	ND	0.0176
5-8-3-1, I-1	ND	ND	ND	ND	ND	ND	ND	0.0074	ND	ND	0.0258
5-8-3-1, I-2	ND	ND	ND	0.0091	ND	ND	ND	0.0083	ND	ND	ND
5-8-3-1, I-3	ND	ND	ND	0.0128	ND	ND	0.0064	0.0158	ND	ND	0.0447
5-8-3-1, I-4	ND	ND	ND	0.0211	ND	ND	0.0067	0.0116	ND	ND	ND
6-3-2-0, I-1	0.1115	2.6473	0.2594	0.4963	0.5678	ND	1.6565	1.7754	0.5857	0.7730	0.8007
6-3-2-0, I-2	ND	ND	ND	ND	ND	0.6749	ND	ND	ND	ND	ND
6-3-2-0, I-3	0.0359	ND	ND	ND	0.1121	0.1174	0.0773	0.1806	ND	ND	ND
6-3-2-0, I-4	0.0548	0.0383	ND	0.0479	0.0550	ND	0.1129	0.0906	0.0682	ND	ND

Supplementary Table S3. Continued…

Glycan	H12 Alpha	H15 Beta	H14 Gamma	H23 Delta	H17 Epsilon	H29 Eta	H28 Iota	H1B Kappa	H32 Lambda	H38 Mu	H41 Omicron
6-3-2-0, I-5	0.0117	ND	ND	ND	0.1321	0.0960	0.0795	0.0294	ND	ND	ND
6-5-1-1, I-1	0.1360	0.0783	0.0689	ND	0.0128	0.0751	ND	0.0701	0.0549	ND	ND
6-5-1-1, I-2	0.1184	0.0605	0.0669	ND	0.1113	ND	0.1174	0.1489	0.0471	ND	ND
6-5-1-1, I-3	0.0944	0.0456	0.1655	ND	0.0303	ND	0.3784	0.1096	ND	0.5359	ND
6-5-1-1, I-4	ND	0.2478	ND	ND	0.1385	ND	ND	ND	0.0098	ND	ND
6-5-1-1, I-5	0.0458	0.0273	0.1828	ND	ND	0.3009	0.0748	0.0308	ND	ND	ND
6-5-1-1, I-6	0.0793	0.0180	ND	ND	0.0542	ND	0.0579	0.0398	ND	ND	ND
6-5-1-1, I-7	0.1567	0.1128	0.1258	0.1568	0.0710	0.1204	0.1931	0.2080	ND	ND	ND
6-5-1-1, I-8	0.0865	0.0542	0.0953	0.2731	0.0448	ND	0.1077	0.1953	ND	ND	ND
6-6-0-1, I-1	ND	ND	ND	ND	ND	ND	ND	ND	ND	ND	0.0141
6-6-0-1, I-2	ND	ND	0.2385	ND	ND	ND	ND	0.0071	ND	ND	0.0184
6-6-0-1, I-3	0.0396	ND	0.0417	0.1007	ND	ND	0.0520	0.0058	0.1756	ND	ND
6-6-0-1, I-4	ND	ND	ND	0.0806	ND	ND	ND	ND	ND	ND	ND
6-6-1-0, I-1	ND	ND	ND	ND	ND	0.0528	ND	ND	ND	ND	ND
6-6-1-0, I-2	ND	ND	ND	ND	ND	ND	ND	ND	ND	ND	0.0849
6-6-1-0, I-3	ND	ND	ND	ND	ND	ND	ND	ND	ND	ND	0.2249
6-6-1-0, I-4	0.2774	ND	0.1741	ND	ND	0.3345	ND	0.1426	ND	0.2808	ND
6-6-1-0, I-5	0.0259	ND	0.0467	0.0708	ND	ND	0.3036	0.0295	0.2440	ND	0.4862
6-6-1-0, I-6	0.4194	ND	0.3353	0.4229	ND	0.0425	0.2178	0.2948	ND	0.4712	ND
6-6-1-0, I-7	ND	ND	ND	ND	ND	0.3380	ND	ND	ND	ND	ND
6-6-1-0, I-8	0.0237	ND	0.0294	0.0211	ND	ND	ND	ND	ND	ND	ND
6-6-1-0, I-9	ND	ND	ND	0.2336	ND	ND	0.0267	ND	ND	ND	ND
6-6-1-1, I-1	ND	ND	ND	ND	ND	ND	ND	ND	ND	ND	0.1280
6-6-1-1, I-2	0.1531	ND	ND	0.0606	ND	ND	0.2823	0.1337	0.3596	ND	0.3108
6-6-1-1, I-3	ND	ND	ND	ND	ND	ND	ND	ND	0.3248	1.6286	0.7408
6-6-1-1, I-4	0.4611	0.0596	0.3195	0.1704	0.0541	ND	0.2680	ND	ND	ND	ND
6-6-1-1, I-5	0.2479	0.7624	0.3148	0.2412	0.2681	0.2072	0.4026	0.2764	0.4132	ND	ND
6-6-1-1, I-6	ND	ND	ND	ND	ND	ND	ND	ND	ND	0.3597	0.1194
6-6-1-1, I-7	0.1749	0.0197	0.1000	0.1707	0.0128	0.4361	0.0682	0.1128	0.1706	0.2037	0.3637
6-6-1-1, I-8	0.0633	0.0999	0.1224	0.1879	0.0531	ND	0.0717	0.1245	ND	ND	ND
6-6-1-1, I-9	ND	ND	ND	ND	ND	0.0835	ND	ND	ND	ND	0.0378
7-4-1-0, I-1	ND	0.0052	ND	ND	ND	ND	ND	ND	ND	ND	ND
7-4-1-0, I-2	ND	0.3851	0.0127	0.0094	0.0213	0.0302	0.0491	0.0252	ND	ND	ND
7-4-1-0, I-3	ND	0.0133	ND	ND	0.0215	ND	0.0253	ND	ND	ND	ND
7-4-1-0, I-4	ND	0.0619	ND	ND	0.0445	ND	ND	ND	0.0942	0.1060	0.0116
7-4-1-0, I-5	ND	0.0313	ND	ND	0.0290	ND	ND	ND	ND	ND	ND
7-4-1-1, I-1	ND	0.0210	ND	0.0073	ND	ND	0.0283	0.0290	ND	ND	ND
7-4-1-1, I-2	ND	ND	ND	0.0075	ND	ND	0.0251	0.0260	ND	ND	0.0069
7-4-1-1, I-3	ND	0.0100	ND	ND	0.0151	ND	ND	ND	ND	ND	ND
7-4-1-1, I-4	ND	0.0117	ND	ND	0.0105	ND	ND	ND	ND	ND	0.0080
7-4-1-1, I-5	ND	0.0379	ND	ND	0.0305	ND	ND	ND	ND	ND	ND
7-4-1-1, I-6	ND	0.0260	ND	ND	0.0170	ND	ND	ND	ND	ND	ND
7-5-0-0, I-1	ND	ND	ND	ND	ND	ND	ND	ND	ND	ND	0.0020
7-5-0-0, I-2	ND	ND	ND	ND	ND	ND	ND	ND	ND	ND	0.0081
7-5-0-0, I-3	0.0802	ND	0.1687	0.0561	ND	ND	ND	0.0333	ND	0.4969	ND
7-5-0-0, I-4	1.4415	ND	5.7667	1.0512	ND	ND	0.1946	0.4382	4.8038	ND	ND
7-5-0-0, I-5	0.5743	ND	1.7513	0.8883	ND	ND	3.3560	0.1626	1.9508	ND	ND
7-5-0-0, I-6	0.0378	ND	ND	0.0285	ND	ND	1.1502	ND	ND	ND	ND
7-5-1-0, I-1	ND	0.0524	0.0220	0.0533	0.0173	ND	0.0369	0.0470	ND	0.0984	0.0479
7-5-1-0, I-2	ND	ND	ND	ND	ND	0.0506	ND	ND	ND	ND	ND
7-5-1-0, I-3	ND	ND	0.0227	0.0397	ND	0.0960	0.0638	ND	ND	ND	ND
7-6-1-1, I-1	ND	ND	ND	ND	ND	0.0748	ND	ND	ND	ND	ND
7-6-1-1, I-2	ND	ND	ND	ND	ND	ND	ND	ND	ND	ND	0.0145
7-6-1-1, I-3	ND	0.0170	ND	ND	0.0149	ND	ND	0.0122	ND	ND	0.0427
7-6-1-1, I-4	0.0100	0.0096	0.0276	0.0117	0.0138	ND	0.0546	0.0245	ND	ND	0.0684
7-6-1-1, I-5	0.0512	0.0968	0.0559	0.0194	0.0720	ND	0.0799	0.0464	ND	0.0459	ND
7-6-1-1, I-6	ND	ND	ND	ND	ND	ND	ND	ND	0.0643	ND	ND
7-6-1-1, I-7	ND	ND	ND	ND	ND	ND	ND	ND	0.0932	ND	ND
7-6-1-2, I-1	1.3307	0.7057	0.9784	0.1045	0.6289	ND	0.3377	0.0858	0.5432	ND	ND
7-6-1-2, I-2	2.2109	1.1687	1.4876	0.2042	1.6499	0.6765	0.4713	0.1008	0.9977	ND	ND
7-6-1-2, I-3	ND	ND	ND	ND	ND	0.8449	ND	ND	ND	ND	ND
7-6-1-2, I-4	1.2138	ND	ND	ND	ND	ND	ND	ND	ND	ND	ND

Supplementary Table S3. Continued…

Glycan	H12 Alpha	H15 Beta	H14 Gamma	H23 Delta	H17 Epsilon	H29 Eta	H28 Iota	H1B Kappa	H32 Lambda	H38 Mu	H41 Omicron
7-6-1-2, I-5	0.3191	ND	ND	ND	ND	ND	ND	ND	ND	ND	ND
7-6-1-2, I-6	1.5496	ND	ND	ND	ND	ND	ND	ND	ND	ND	ND
7-6-1-2, I-7	0.1109	ND	ND	ND	ND	ND	ND	ND	ND	ND	ND
7-6-1-2, I-8	0.1996	ND	ND	ND	ND	ND	ND	ND	ND	ND	ND
7-6-1-3, I-1	ND	ND	ND	ND	ND	ND	ND	ND	ND	ND	0.0587
7-6-1-3, I-2	ND	0.0509	ND	0.0363	0.0086	ND	0.0463	0.0522	0.2350	ND	0.0178
7-6-1-3, I-3	ND	ND	ND	ND	ND	ND	ND	ND	ND	ND	0.0413
7-6-1-3, I-4	ND	0.0884	ND	0.0456	0.0138	ND	0.0510	0.0441	0.4927	0.3820	0.0126
7-6-1-3, I-5	ND	0.0350	ND	0.0546	0.0311	0.0743	0.0639	0.0418	ND	ND	0.0089
7-7-0-1, I-1	0.0646	ND	0.2186	ND	ND	0.0138	ND	0.0143	ND	0.1220	ND
7-7-0-1, I-2	0.2011	ND	0.6599	0.0796	ND	ND	0.0890	0.0321	ND	ND	ND
7-7-0-1, I-3	0.0505	ND	0.2002	0.2033	ND	ND	0.2101	ND	ND	ND	ND
7-7-0-1, I-4	0.2331	ND	0.9095	0.4107	ND	ND	0.2187	0.0527	ND	ND	ND
7-7-0-1, I-5	0.0691	ND	0.1804	0.1699	ND	ND	0.0652	ND	ND	ND	ND
7-7-0-1, I-6	0.0583	ND	0.1669	0.1264	ND	ND	0.0501	0.0242	ND	ND	ND
7-7-0-1, I-7	0.0717	ND	0.2234	0.2411	ND	ND	0.0532	ND	ND	ND	ND
7-7-1-0, I-1	ND	ND	ND	ND	ND	ND	ND	ND	ND	ND	0.2755
7-7-1-0, I-2	0.1862	0.6364	0.2334	0.1164	0.2575	0.1014	0.2097	0.1011	0.3750	ND	ND
7-7-1-0, I-3	ND	ND	ND	ND	ND	ND	ND	ND	ND	ND	0.0216
7-7-1-1, I-1	0.1411	0.6006	0.3452	0.1385	0.3513	ND	0.1891	0.1475	ND	0.3365	ND
7-7-1-1, I-2	ND	0.0666	ND	0.0336	0.0888	0.2203	0.0531	0.0310	ND	ND	ND
7-7-1-1, I-3	ND	ND	ND	ND	ND	ND	ND	ND	ND	ND	0.0036
7-7-1-1, I-4	ND	ND	ND	ND	ND	ND	ND	ND	ND	ND	0.0146
7-7-1-2, I-1	ND	ND	ND	ND	ND	ND	ND	ND	ND	ND	0.0360
7-7-1-2, I-2	0.0140	0.0334	0.0419	0.0541	0.0248	ND	0.0421	0.0443	ND	ND	0.0548
7-7-1-2, I-3	0.0121	0.0295	0.0334	0.0645	0.0228	0.0492	0.0413	0.0627	ND	ND	0.1040
7-7-1-2, I-4	0.0153	0.1868	0.0678	0.0991	0.0814	ND	0.0690	0.0726	ND	ND	ND
7-7-1-3, I-1	ND	ND	ND	ND	ND	ND	ND	ND	ND	ND	0.0087
7-7-1-3, I-2	ND	ND	ND	ND	ND	ND	ND	ND	ND	ND	0.0607
7-7-1-3, I-3	ND	ND	ND	ND	ND	ND	ND	ND	ND	ND	0.0262
7-7-1-3, I-4	0.0031	0.0305	0.0247	0.0516	0.0110	ND	0.0206	0.0390	0.0242	0.0513	0.0504
7-7-1-3, I-5	0.0040	0.0278	0.0249	0.0749	0.0063	ND	0.0329	0.0482	0.0481	0.0475	0.0018
7-7-1-3, I-6	ND	ND	ND	ND	ND	ND	ND	ND	ND	ND	0.0121
7-8-1-1, I-1	ND	ND	ND	ND	ND	ND	ND	ND	ND	ND	0.0839
7-8-1-1, I-2	0.0188	0.0166	0.0698	0.0574	0.0378	ND	0.1282	0.0775	ND	ND	1.1035
7-8-1-1, I-3	0.2064	0.5721	0.7821	0.9413	0.3828	ND	0.7080	0.8701	ND	ND	2.4585
7-8-1-1, I-4	0.2940	0.9151	1.0163	1.4167	0.7377	ND	0.7752	0.7104	ND	ND	ND
7-8-1-1, I-5	ND	ND	ND	ND	ND	ND	ND	ND	ND	ND	0.0665
7-8-1-1, I-6	0.0196	ND	ND	0.0309	ND	ND	ND	0.0351	ND	ND	0.1154
7-8-1-1, I-7	0.0332	ND	ND	0.0770	ND	ND	ND	0.0413	ND	ND	ND
7-8-1-2, I-1	ND	ND	ND	ND	ND	ND	ND	ND	ND	ND	0.0053
7-8-1-2, I-2	ND	ND	ND	ND	ND	ND	ND	ND	ND	ND	0.0073
7-8-1-2, I-3	0.0189	0.0043	0.0205	0.0201	0.0053	ND	0.0168	0.0252	ND	0.0492	0.0063
7-8-1-2, I-4	ND	ND	ND	ND	ND	ND	ND	ND	ND	ND	0.0010
8-6-1-0	ND	ND	ND	ND	ND	ND	ND	0.1478	ND	ND	ND
8-6-1-1, I-1	ND	ND	ND	ND	ND	ND	ND	ND	ND	ND	0.0091
8-6-1-1, I-2	ND	ND	ND	ND	ND	ND	ND	ND	ND	ND	0.0222
8-6-1-1, I-3	ND	0.0240	ND	0.0053	ND	ND	0.0093	0.0090	ND	ND	0.0044
8-8-0-0, I-1	0.0575	ND	0.0846	ND	ND	ND	ND	0.0374	ND	ND	ND
8-8-0-0, I-2	0.0566	ND	0.1078	ND	ND	ND	ND	ND	ND	ND	ND
8-8-0-0, I-3	0.0166	ND	0.0401	ND	ND	ND	ND	ND	ND	ND	ND
8-8-0-0, I-4	0.0172	ND	0.0261	ND	ND	ND	ND	0.0261	ND	ND	ND
8-8-0-0, I-5	0.0102	ND	0.0170	ND	ND	ND	ND	0.0256	ND	ND	ND
8-8-1-2, I-1	0.0057	0.0156	ND	ND	ND	ND	ND	0.0150	ND	ND	0.0310
8-8-1-2, I-2	0.0768	ND	ND	ND	ND	ND	ND	0.0469	ND	ND	0.0586
8-8-1-2, I-3	0.2387	ND	ND	ND	ND	ND	ND	0.0428	ND	ND	ND
8-8-1-2, I-4	0.0735	ND	ND	ND	ND	0.2159	ND	0.0244	ND	ND	ND
8-8-1-2, I-5	0.1305	ND	ND	ND	ND	0.5582	ND	0.0224	ND	ND	ND
8-8-1-2, I-6	0.0254	ND	ND	ND	ND	0.1138	ND	0.0221	ND	ND	ND
8-9-0-2, I-1	0.0934	ND	0.1511	ND	ND	ND	ND	0.1199	ND	0.3249	0.0997
8-9-0-2, I-2	ND	ND	ND	ND	ND	0.2732	ND	ND	ND	ND	ND
